# Pipeline for specific subtype amplification and drug resistance detection in hepatitis C virus

**DOI:** 10.1186/s12879-018-3356-6

**Published:** 2018-09-03

**Authors:** María Eugenia Soria, Josep Gregori, Qian Chen, Damir García-Cehic, Meritxell Llorens, Ana I. de Ávila, Nathan M. Beach, Esteban Domingo, Francisco Rodríguez-Frías, María Buti, Rafael Esteban, Juan Ignacio Esteban, Josep Quer, Celia Perales

**Affiliations:** 1 Liver Unit, Internal Medicine Hospital Universitari Vall d’Hebron, Vall d’Hebron Institut de Recerca (VHIR), Barcelona, Spain; 2 Centro de Investigación Biomédica en Red de Enfermedades Hepáticas y Digestivas (CIBERehd) del Instituto de Salud Carlos III, Madrid, Spain; 3 Roche Diagnostics, S.L, Sant Cugat del Vallés, Barcelona, Spain; 4 Centro de Biología Molecular “Severo Ochoa” (CSIC-UAM), Consejo Superior de Investigaciones Científicas (CSIC), Campus de Cantoblanco, Madrid, Spain; 5 Liver Pathology Unit, Department of Biochemistry and Microbiology, Hospital Universitari Vall d’Hebron, Barcelona, Spain; 6 Universitat Autónoma de Barcelona, Barcelona, Spain

**Keywords:** Next-generation sequencing, Viral quasispecies, Antiviral agents, Viral diagnostics, Treatment planning

## Abstract

**Background:**

Despite the high sustained virological response rates achieved with current directly-acting antiviral agents (DAAs) against hepatitis C virus (HCV), around 5–10% of treated patients do not respond to current antiviral therapies, and basal resistance to DAAs is increasingly detected among treatment-naïve infected individuals. Identification of amino acid substitutions (including those in minority variants) associated with treatment failure requires analytical designs that take into account the high diversification of HCV in more than 86 subtypes according to the ICTV website (June 2017).

**Methods:**

The methodology has involved five sequential steps: (i) to design 280 oligonucleotide primers (some including a maximum of three degenerate positions), and of which 120 were tested to amplify NS3, NS5A-, and NS5B-coding regions in a subtype-specific manner, (ii) to define a reference sequence for each subtype, (iii) to perform experimental controls to define a cut-off value for detection of minority amino acids, (iv) to establish bioinformatics’ tools to quantify amino acid replacements, and (v) to validate the procedure with patient samples.

**Results:**

A robust ultra-deep sequencing procedure to analyze HCV circulating in serum samples from patients infected with virus that belongs to the ten most prevalent subtypes worldwide: 1a, 1b, 2a, 2b, 2c, 2j, 3a, 4d, 4e, 4f has been developed. Oligonucleotide primers are subtype-specific. A cut-off value of 1% mutant frequency has been established for individual mutations and haplotypes.

**Conclusion:**

The methodological pipeline described here is adequate to characterize in-depth mutant spectra of HCV populations, and it provides a tool to understand HCV diversification and treatment failures. The pipeline can be periodically extended in the event of HCV diversification into new genotypes or subtypes, and provides a framework applicable to other RNA viral pathogens, with potential to couple detection of drug-resistant mutations with treatment planning.

**Electronic supplementary material:**

The online version of this article (10.1186/s12879-018-3356-6) contains supplementary material, which is available to authorized users.

## Background

Hepatitis C virus (HCV) circulates in nature as complex distributions of mutants known as viral quasispecies [1, 2], and currently chronically infects around 71 million people worldwide [3]. HCV is divided into seven major genotypes, and 86 confirmed subtypes (according to ICTV website https://talk.ictvonline.org/ictv_wikis/flaviviridae/w/sg_flavi/56/hcv-classification, June 2017), being genotype 1 the most prevalent worldwide, followed by genotype 3 [4, 5]. Although direct-acting antiviral agents (DAAs) against NS3-4A, NS5A, and NS5B have significantly increased the sustained virological response (SVR) rates, few treatment regimens are effective against all major genotypes [6, 7]. A recent global epidemiological survey of HCV subtypes evidenced the complexity of accurate subtype determination, and variations in regional subtype prevalence [8]. In addition to the viral genotype and subtype, other factors, including the degree of fibrosis, baseline viral load and mutational spectrum of the resident virus influence the efficacy of anti-DAA combinations. An additional issue, which is inherent to quasispecies dynamics, is the selection of DAA-resistant mutants, a significant problem in the control of HCV infections [9–14]. DAA-resistance mutations are detected in the HCV of an increasing number of treatment-naïve patients [15–17], and current evidence suggests that they can be selected upon treatment with DAAs (reviewed in [18, 19]).

Despite DAAs offering a high genetic barrier to resistance, especially NS5B nucleos(t)ide inhibitors, there is an increasing list of mutations which are related to resistance to DAAs in clinical use, including sofosbuvir [9, 10, 14, 20–24]. Current recommendations of baseline HCV resistance testing, according to AASLD/IDSA and EASL guidelines 2016, are mainly aimed at treatment optimization to determine dosage regimen, treatment duration and the need to include ribavirin. NS5A-resistance analyses are recommended prior to the initial treatment with elbasvir/grazoprevir in G1a HCV-infected patients [25], and detection of Q80K polymorphism is advised before sofosbuvir/simeprevir therapy in infections with G1a HCV. In the re-treatment of patients who failed previous anti-HCV therapies, baseline resistance is recommended prior to combinations that include sofosbuvir/velpatasvir for G3, sofosbuvir/daclatasvir for Gs 1a and 3, sofosbuvir/ledipasvir for G1a, and elbasvir/grazoprevir and sofosbuvir/simeprevir for G1a and G1b [6]. These recommendations can be summarized as the need to determine HCV mutational spectra in patients requiring salvage treatments [26].

Several tools to analyze drug resistance in preclinical and clinical studies are available [27]. The potential of deep-sequencing to routinely analyze minority variants containing resistance-associated substitutions (RAS) has several drawbacks. First, HCV intrapopulation dynamics, and rates of evolution reaching 10^− 2^ to 10^− 3^ substitutions per site per year may decrease the efficacy of some oligonucleotide primers [2]. Second, deep sequencing requires prior control experiments to ensure experimental conditions and bioinformatic processing that minimize the scoring of sequencing errors inherent to the methodology, as well as recombination during PCR amplification [28–30]. An analysis of HCV resistance should ideally include standardized protocols that encompass the RNA amplification steps and the bioinformatics pipeline, to enable the determination of a reliable cut-off value for mutation frequency. Here we describe the design of subtype-specific oligonucleotides, followed by the amplification of HCV RNA from infected patients, and ultra-deep sequencing analysis with controls for reliable detection of amino acid subtitutions and RAS. The rationale to design subtype-specific primers is to avoid bias in the PCR amplification, because the genomic regions of interest whose proteins are targeted by the antiviral agents display an important degree of variation among subtypes. We further describe bioinformatic procedures to filter variants that result from technical errors, and the comparison with a reference sequence defined for each subtype. Finally, tests are validated with HCV samples from infected patients, and mutations that have been previously classified as RAS are reported. The proposed methodological pipeline can be adapted to new HCV genotypes or subtypes, and extended to other rapidly evolving RNA viral pathogens.

## Methods

### HCV database and primer design

The subtype-specific oligonucleotides were designed based on HCV sequence alignments from Los Alamos HCV database (https://hcv.lanl.gov/content/sequence/HCV/ToolsOutline.html). The sequences were retrieved with the inclusion criteria of belonging to full genome sequences (confirmed non-recombinant genomes), being devoid of large insertions/deletions, and corresponding to Gs 1a, 1b, 2a, 2b, 2c, 2j, 3a, 4a, 4d, and 4 f. Subtypes 1a and 1b were chosen due to their worldwide prevalence [5], and the other subtypes were included based on the availability of samples in our patient’s cohort. The accession numbers of the collected sequences are provided as suplementary material (Additional file 1: Table S1).

### Production of a triple HCV mutant

The cDNA that expresses wild-type HCV [plasmid Jc1FLAG2(p7-nsGluc2A)] [31] was used as the genomic backbone for the construction of the infectious clone encoding NS5A with replacements N248H, E269K and A346V by overlap extension PCRs. Three overlapping PCR fragments (B1, B2, and B3) were assembled. Oligonucleotide primers used to synthesize PCR fragment B1 were: Jc1-NS5A-F2 and Bch-0-3; for PCR fragment B2 the primers were: Bch-0-4, and Bch-0-21; for PCR fragment B3 the primers were: Bch-0-6, and Jc1-NS5B-R1 (primer sequence and location are given in Additional file 2: Table S2). The fusion PCR products were then cloned into full-length HCV plasmid using SanD1 and BsrGI (New England Biolabs), and sequenced to confirm that no unwanted mutations had been introduced. Since primer Jc1NS5AM13d7131 excluded mutation A346V from the amplification product, mutations N248H and E269K remained as marker for recombination. The RNA transcript was produced as previously described [32], using T7 RiboMAX Express Large Scale RNA Production system Catalog (Promega). Quantitative real-time PCR (qRT-PCR) of HCV RNA was carried out in triplicate using a light Cycler RNA Master SYBR green I kit (Roche), as described previously [33]. The 5′ untranslated region (5’-UTR) of the HCV genome was amplified with oligonucleotides HCV-5UTR-F2, and HCV-5UTR-R2 as primers (Additional file 2: Table S2). Quantification was relative to a standard curve obtained with known amounts of HCV RNA synthesized by in vitro transcription of HCV cDNA (plasmid GNN) [31]. The specificity of the reaction was monitored by determining the denaturation curve of the amplified DNAs. Negative controls were run in parallel with each amplification reaction to ascertain the absence of contamination with undesired templates.

### Control of basal nucleotide sequencing error

To determine the basal error of the process that leads to mutant spectrum characterization, 100,000 DNA molecules of the infectious clone encoding NS5A with replacements N248H, E269K and A346V were used to perform external, internal and Multiplex IDentifier (MID) PCRs (the latter when applied). The first external PCR was carried out using Transcriptor One Step RT-PCR kit (Roche Applied Science). To perform the external PCR, 100,000 DNA molecules were mixed with 10 μl of 5× buffer [including Tris, MgCl_2_, sodium salts of dNTPs (1.5 mM each) and additives for hot start PCR], and 0.4 μM of Fw and Rv primers. The reaction parameters were an initial denaturing step at 94 °C for 7 min, followed by 35 cycles of a denaturing step at 94 °C for 10 s, an anneling step at 55 °C for 30 s, an extension step at 68 °C for 40 s, and then a final extension at 68 °C for 7 min. The external PCR was performed with the oligonucleotides Jc1NS5Au6521 and Jc1NS5Ad7211 to generate an amplicon size of 691 bp (Additional file 2: Table S2). Since DNA was the starting material, no RT step was included. The products of the first round PCR were then subjected to an internal PCR using FastStart Taq DNA polymerase (Roche Applied Science). To perform the internal PCR, 5 μl DNA from the first round PCR was mixed with 5 μl of 10× buffer, 0.8 mM of dNTPs, and 0.4 μM of Fw and Rv PCR primers. The reaction parameters were an initial denaturing step at 94 °C for 4 min, followed by 30 cycles of a denaturing step at 94 °C for 30 s, an anneling step at 55 °C for 30 s, an extension step at 72 °C for 40 s, and then a final extension at 72 °C for 7 min. The internal PCR was performed with the oligonucleotides Jc1NS5AM13u6693 and Jc1NS5AM13d7131 to generate an amplicon size of 439 bp (Additional file 2: Table S2).

PCR products were then subjected to a MID PCR using FastStart Taq DNA polymerase (Roche Applied Science) only when the 454 GS-Junior platform was used. The oligonucleotides were composed of a complementary universal M13 primer (either upstream or downstream) and a Roche’s Validated MID, with oligonucleotide A or B at the 5′ or 3′ end of the upstream or downstream primer, respectively. To perform the MID PCR, 5 μl from the internal PCR solution were mixed with 5 μl of 10× buffer, 0.8 mM of dNTPs, and 0.4 μM of Fw and Rv PCR primers. The reaction parameters were an initial denaturing step at 94 °C for 4 min, followed by 15 cycles of a denaturing step at 94 °C for 30 s, an anneling step at 60 °C for 30 s, an extension step at 72 °C for 40 s, and a final extension at 72 °C for 7 min. The final MID amplification yielded 549 bp fragments.

For the theoretical approach, sampling molecules of N types independently corresponds to sampling a multinomial distribution with N categories Multi(N; π_1_, π_2_,. .., π_i_,. .., π_N_). Each category individually behaves as a binomial with *p* = π_i_, conditional on the others. To find the minimum coverage to reliably detect haplotype mutants at 1% abundance, with a noise level at 0.5%, we based the computations on two binomial distributions, Binom(N,p), with p_1_ at 1% and p_2_ at 0.5% and found the confidence interval at different levels of confidence (95%, 99%, 99.5% and 99.9%) for different coverages (N) in the range 500–10,000 reads.

### Control of PCR recombination

To determine the frequency of PCR recombination in the course of the amplification steps, 100,000 total DNA molecules comprised of wt and mutant (infectious clone encoding NS5A with replacements N248H, E269K and A346V) clones were mixed at a 90:10 ratio. This mixture was subjected to four amplification protocols. Protocol 1 consisted in three PCRs (external PCR, internal PCR, and MID PCR), as previously described for determination of the basal error. Protocol 2 included also three PCRs but the oligonucleotide concentration and the elongation time were increased from 0.4 to 1 μM and from 40 to 60 s, respectively, and the number of cycles was decreased from 80 to 60 (30 cycles for external PCR, 20 cycles for internal PCR, and 10 cycles for MID PCR). Protocols 3 and 4 consisted in two PCRs (external PCR, and MID PCR); the internal PCR was eliminated. For the external PCR the oligonucleotides that included M13 were Jc1NS5AM13u6693 and Jc1NS5AM13d7131, previously described (Additional file 2: Table S2). In terms of oligonucleotide concentration, elongation time and number of cycles of protocols 3 and 4 were similar to protocols 1 and 2, respectively. The experiments with the four protocols were performed in duplicate.

### Deep-sequencing amplification (NGS)

Amplification products were analyzed by 2% agarose gel electrophoresis using GeneRuler 1 kb Plus DNA Ladder (Thermo Scientific) as the molar mass standard, and purified using QIAquick gel extraction kit (Qiagen). Negative controls (amplifications in the absence of RNA) were included in parallel to ascertain absence of contamination by template nucleic acids. DNA quantifications were performed using the PicoGreen assay (Invitrogen), or the Qubit dsDNA Assay kit (ThermoFisher Scientific). Amplicon quality was analyzed using a BioAnalyzer DNA 1000 LabChip (Agilent) prior to sequencing using 454 GS-Junior or Illumina MiSeq platforms. A description of the viral samples sequenced by both platforms is recopilated in Additional file 3: Table S3.

Sequencing using 454 GS-Junior has been previously described [34]. For the sequencing using MiSeq Illumina, amplification products of the NS3, NS5A and NS5B DNAs were adjusted to the same concentration and pooled. The amplicon pools were purified using Ampure Beads Agentcourt AMPure XP (Beckman Coulter Inc., Danvers, MA, USA), or Kapa Pure Beads (Kapabiosystems, Roche) to remove primers, nucleotides, salts and enzymes. The purified product was quantified using Qubit as previously described, before generating sDNA pools of 100–250 ng. Purified pools were processed following the DNA library preparation kit Kapa Hyper Prep kit (Roche), during which each pool was indexed using SeqCap Adapter Kit A/B (Nimblegen) (24 Index). Each DNA pool was ajusted to 4 nM concentration and appropriate volumes of each pool were added to the final library, which was quantified by LightCycler 480 (Kapa Library Quantification kit), and sequenced using MiSeq sequencing platform with MiSeq Reagent kit v3 (2 × 300 bp mode with the 600 cycle kit) (Illumina, San Diego, CA). Each run may include as a maximum 24 pools, being the total number of amplicons approximately 96.

### Amplification of HCV RNA from patient samples

Viral RNAs from four HCV-infected patients at DAA treatment failure, corresponding to G1a, G1b, G3a, and G4d were selected. HCV genotype assignment had been previously performed with the high resolution subtyping using UDPS [34]. Only samples previously subtyped by ultra-deep pyrosequencing were used. HCV RNA was extracted from 140 μl of plasma/serum of patient samples by manual RNA extraction, using the Qiagen Total RNA extraction kit (Qiagen, Hilden, Germany), as specified by the manufacturer. The measures to prevent contamination suggested by Kwok and Higuchi were strictly applied [35].

Amplifications of NS3-, NS5A-, and NS5B-coding regions were performed using a RT-PCR, followed by an internal PCR with specific primers covering the three regions of interest (NS3, NS5A and NS5B) (Additional files 4: Figure S1, Additional file 5: Figure S2 and Additional file 6: Figure S3, and primer sequence and location are given in Additional file 7: Table S4, Additional file 8: Table S5 and Additional file 9: Table S6). Each target region was amplified from 5 to 10 μl of the purified RNA solution by an RT-PCR for each target region using Transcriptor One Step RT-PCR kit (Roche Applied Science). To perform the RT-PCR, 5–10 μl RNA was mixed with 10 μl of 5× buffer, 0.4 μM of Fw and Rv PCR primers (0.6 μM if degenerate primers were used). The reaction parameters were 50 °C for 30 min for the reverse transcription, an initial denaturing step at 94 °C for 7 min, followed by 35 cycles of a denaturing step at 94 °C for 10 s, an anneling step at 50–55 °C for 30 s, an extension step at 68 °C for 40 s, and then a final extension at 68 °C for 7 min.

The products of the first round PCR were then subjected to an internal PCR using FastStart Taq DNA polymerase (Roche Applied Science). To perform the nested PCR, 5–10 μl of the solution from the first round PCR was mixed with 5 μl of 10× buffer, 0.8 mM of dNTPs, 0.4 μM of Fw and Rv PCR primers (0.6 μM if degenerate primers were used). The reaction parameters were an initial denaturing step at 94 °C for 4 min, followed by 30 cycles of a denaturing step at 94 °C for 30 s, an anneling step at 45–55 °C for 30 s, an extension step at 72 °C for 40 s, and then a final extension at 72 °C for 7 min.

### Comparative analysis using inter- and intra- sequencing platforms

Although it is often assumed that the major source of variability is a feature of the amplicon preparation steps, erroneous variations might also originate from the DNA sequencing procedure itself. To establish the reproducibility of the entire process, amplicons were divided in two aliquots and processed in parallel either on different platforms (454 GS-Junior and Illumina MiSeq) (inter-platform comparison), or on different runs in the same platform (Illumina MiSeq) (intra-platform comparison). Inter- and intra-platforms reproducibility was assessed for four independent amplicons of two patient samples. Percentages of variation were highly reproducible in parallel experiments, as evidenced by the scatter plots (Pearson correlation ≥0.90) (Additional file 10: Figure S4).

### Nucleotide sequence accession numbers

Ultra deep sequencing data from HCV-infected patient samples have been deposited in GenBank under accession numbers SAMN08741670 to SAMN08741677 (SRA accession: SRP136087 and Bioproject ID: PRJNA439187).

## Results

### Validation of subtype-specific oligonucleotide primers

**Fig. 1:**
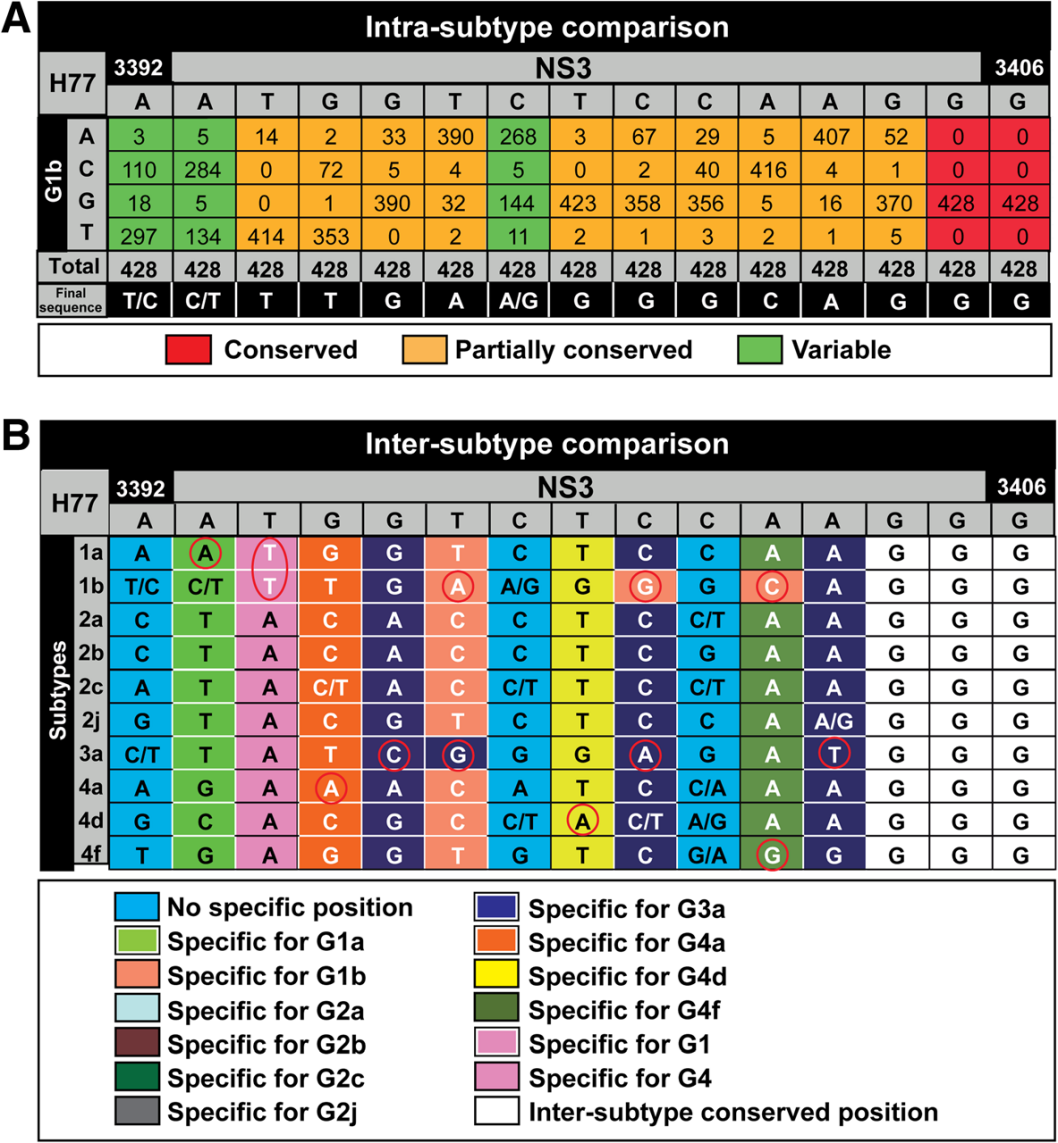
Design of subtype-specific oligonucleotides. A total of 1,936,116 nucleotides at 1,638 positions along the HCV genome belonging to the ten HCV subtypes under study were analyzed. **a** Example of an intra-subtype comparison from position 3392 to position 3406 within the NS2-coding region (sequence in gray at the top) for HCV subtype 1b. The nucleotide numbering is based on the sequence of HCV strain H77 (GenBank accession AF009606). Numbers in boxes represent the number of sequences that for each position have the nucleotides written on the left grey column. Positions have been defined as conserved (in red) when they contain a single nucleotide type, partially conserved (in orange) when they contain at least two nucleotides (but only one of them with a frequency above 20% of the total number of sequences), and variable (in green) when two nucleotide types have a frequency above 20% of the total number of sequences. Only in variable positions a mixture of two nucleotides is considered to define the column (see the bottom raw termed “Final sequence”). **b** Example of an inter-subtype comparison from position 3392 to position 3406 within the NS2-coding region (sequence in gray at the top). Final sequences —defined as described in (A) for the HCV subtypes 1a, 1b, 2a, 2b, 2c, 2j, 3a, 4a, 4d, and 4f— were compared to define positions that are discriminatory for a specific subtype and/or genotype (color codes given in the bottom box). Red circles highlight the nucleotides specific for a defined subtype and/or genotype. Excel file including all comparisons will be made available upon request

To design subtype-specific oligonucleotide primers spanning HCV NS3-, NS5A- and NS5B-coding regions, a total of 1,182 sequences from Los Alamos HCV database (553, 427, 33, 79, 7, 5, 49, 18, 5, and 6 sequences from genotypes 1a, 1b, 2a, 2b, 2c, 2j, 3a, 4a, 4d, and 4f, respectively) entered the study. They were analyzed phylogenetically to confirm that they belong to the assigned subtypes. Nucleotide composition was analyzed from position 3368 to 3530 and 3910 to 4010 to design forward (Fw) and reverse (Rv) oligonucleotide primers, respectively, to amplify the NS3-coding region (residue numbering according to the reference strain AF009606). Likewise, position 6150 to 6330; 6710 to 6740, and 6770 to 6970 were examined to design Fw and Rv oligonucleotides to amplify the NS5A-coding region, and positions 7550 to 7650; 7940 to 8080; 8130 to 8250; 8360 to 8400; 8494 to 8514; 8550 to 8660; 8750 to 8880; 9000 to 9199 and 9327 to 9420 were examined to design Fw and Rv primers to amplify the NS5B-coding region. Fasta files with the sequence alignment for each subtype were used to determine the frequency of each nucleotide at each position (Excel files including all comparisons are available upon request). Comparison of the sequences that belong to the same subtype (intra-subtype comparison) (Fig. 1a) assigned each nucleotide position to one of three categories: 1) “conserved” (in red), when all sequences contain the same nucleotide at that position; 2) “partially conserved” (in orange), when a position can have more than one nucleotide type but only one of them at a frequency above 20% of the total number of sequences, and 3) “variable” (in green), when two nucleotide types have a frequency above 20% of the total number of sequences. To design oligonucleotide primers, “partially conserved” positions were represented by the most frequent nucleotide, and “variable” positions were degenerated. With these criteria, a total of 1,936,116 nucleotides from sequences at 1,638 positions belonging to the ten HCV subtypes under study were analyzed.

**Fig. 2:**
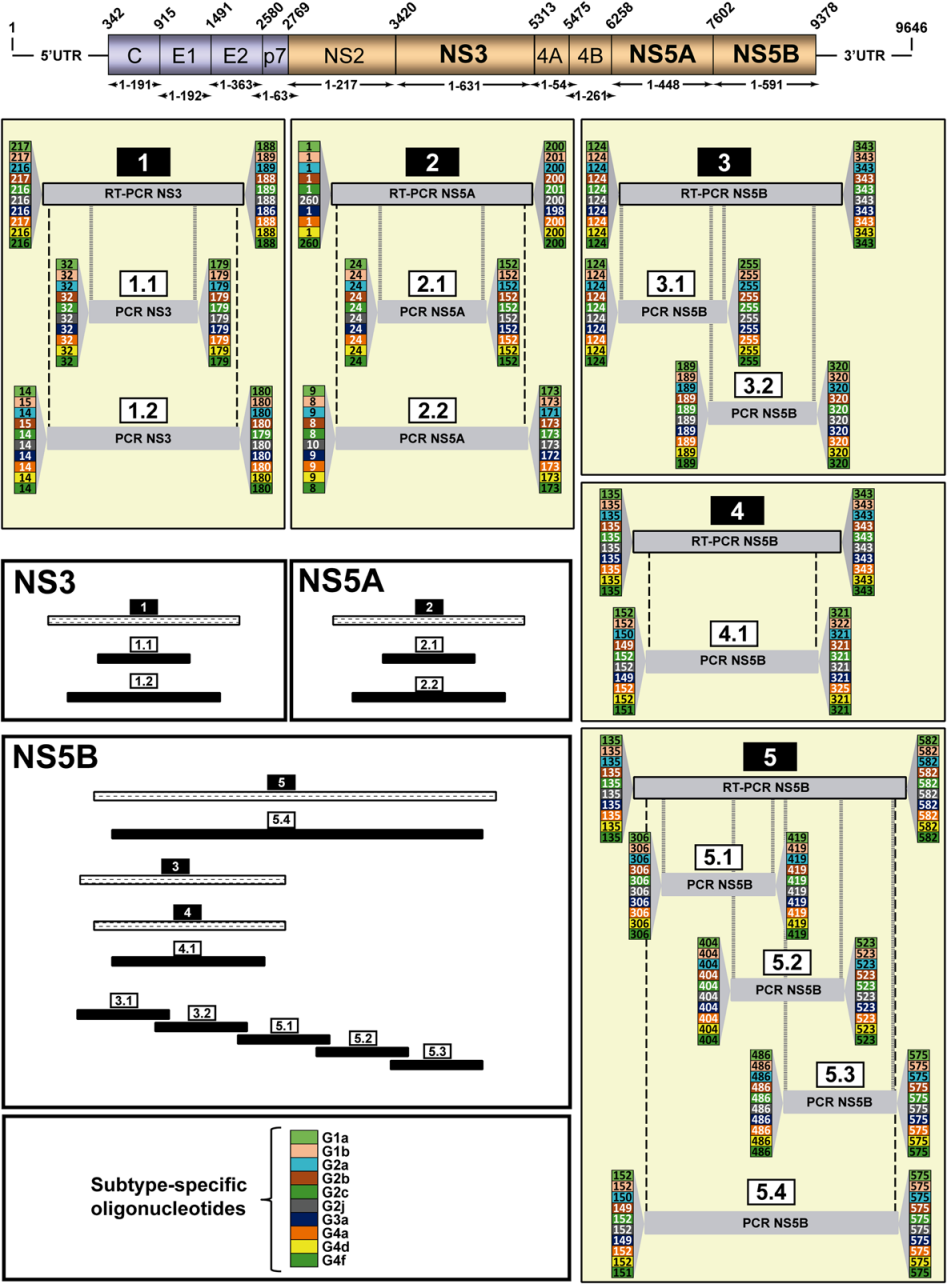
Scheme of the RT-PCRs and PCRs designed to amplify HCV NS3-, NS5A, and NS5B-coding regions. Top: Scheme of the HCV genome with indication of nucleotide numbering (above the genome), and amino acid numbering for individual proteins (below the genome); numbering is defined according to [49]. Regions chosen to design the oligonucleotides described in the present study were those that include several subtype-specific positions, and that amplify regions that have been associated with DAA resistance. RT-PCRs labeled 1, 2 correspond to NS3-, NS5A- coding region, respectively, and 3, 4 and 5 to NS5B-coding region (numbers in black rectangles above the identified RT-PCR). Internal PCRs with different sizes can interrogate different positions in the HCV genome, and can be chosen according to the amplicon size restrictions imposed by the sequencing platform (several possibilities are depicted with identification numbers in white rectangles). Numbering at the beginning and at the end of each PCR (columns with colored numbers; color codes in the bottom box) correspond to the first and last amino acid of the amplicon, excluding oligonucleotides. When the number of the first amino acid is higher than the number corresponding to the reverse oligonucleotide means that the forward oligonucleotide is located in the gene upstream from the region to be amplified. Relative sizes of standard and internal PCRs for NS3, NS5A and NS5B are depicted comparatively in the white large boxes

The inter-subtype comparison defined positions that were discriminatory for a specific subtype (Fig. 1b). The regions chosen preferentially for primer design were those that include several subtype-specific positions (Additional file 4: Figure S1, Additional file 5: Figure S2 and Additional file 6: Figure S3). A total of 280 oligonucleotides (60 for NS3-, 60 for NS5A-, and 160 for NS5B-coding region), with a length range between 18 and 30 nucleotides, a maximum of three degenerate positions per oligonucleotide, and melting temperatures ranging from 47.7 °C to 66.1 °C were designed (Additional files 7: Table S4, Additional file 8: Table S5 and Additional file 9: Table S6, and Additional files 4: Figure S1, Additional file 5: Figure S2 and Additional file 6: Figure S3). These oligonucleotides were used to amplify the NS3, NS5A, and NS5B-coding regions with several external and nested PCRs (Fig. 2).

**Fig. 3:**
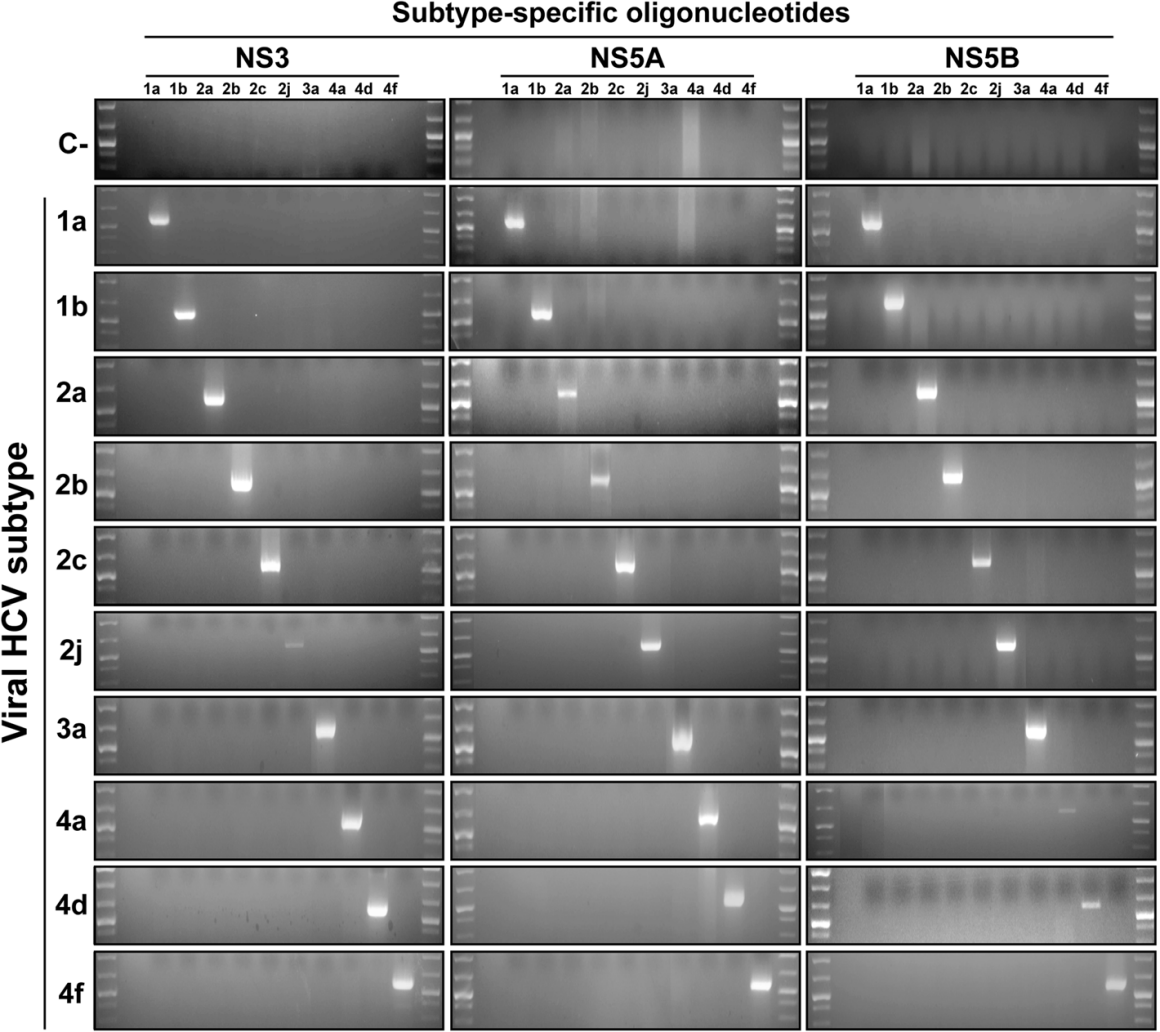
Specific amplification with subtype-specific oligonucleotides in patient samples infected with HCV. Ten viral RNAs that correspond to subtypes 1a, 1b, 2a, 2b, 2c, 2j, 3a, 4a, 4d, 4f (left colum) isolated from HCV-infected patients were subtyped using next-generation sequencing [34], and used to amplify NS3-, NS5A-, and NS5B-coding regions with subtype-specific oligonucleotides. Viral RNA of each subtype was confronted with oligonucleotide pairs of the subtypes under study (listed at the top, below the genomic region). A total of 120 oligonucleotide primers out of 280 were tested. Amplifications correspond to RT-PCR 1 and PCR 1.2 in the NS3-coding region, RT-PCR 2 and PCR 2.2 in the NS5A-coding region, and RT-PCR 4 and PCR 4.1 in the NS5B-coding region, as depicted in Fig. 2. C-, negative control, amplification without RNA. Conditions of amplifications are detailed in Materials and Methods

As a proof-of-concept that the oligonucleotide design resulted in subtype-specific amplifications, viral RNAs from infected patients belonging to each of the subtypes (assigned by ultra-deep sequencing [34]) were used to perform amplifications within the NS3-, NS5A-, and NS5B-coding regions [external RT-PCRs 1, 2 y 4, and nested PCRs 1.2, 2.2 and 4.1 (Fig. 2)]. Each RNA was confronted with all oligonucleotide pairs. Amplifications were positive only when the HCV subtype of the viral RNA corresponded with the subtype for which the oligonucleotides were devised (Fig. 3). This result validates the procedure used to design oligonucleotides to amplify HCV genomes in a subtype-specific manner.

### Limit of detection of individual amino acid substitutions: Experimental and theoretical approaches

Previous studies from our group have defined a sequence depth of at least 10,000 reads per DNA strand to achieve a 0.5% cut-off value for mutant frequency at the nucleotide level using the 454 pyrosequencing system (Roche) [36]. Here we have performed again the main controls of the whole process for three main reasons: 1) the enzymes chosen for the amplification steps were different from those previously used (see Materials and Methods); 2) the bioinformatics procedures were modified for amino acid rather than nucleotide analyses; and 3) we have compared a previous platform 454 GS-Junior (Roche) with MiSeq (Illumina) that we subsequently implemented. The first control was the determination of the basal error of the entire process which includes the amplification steps and sequencing. To this aim, a full-length HCV DNA encoding NS5A with amino acid substitutions N248H, E269K, and A346V (associated with IFN-α resistance [32]) was used as template. Only the genomic region corresponding to N248H and E269K was amplified in this control. Triplicate amplifications of the NS5A-coding region and sequencing were performed with platforms 454 GS-Junior and MiSeq (Additional file 11: Figure S5). Mutations other than those leading to N248 K and E269K were considered artifacts introduced during amplicon synthesis and/or sequencing. Erroneous individual amino acid substitutions were found at a maximum frequency of 0.50 ± 0.05%, and 0.63 ± 0.1% for 454 GS-Junior and MiSeq platforms, respectively. Similar error frequency (0.89 ± 0.24) was obtained using RNA as starting material. These results established a conservative limit of detection for individual amino acid substitutions at 1% frequency.

An important question in the mutant spectrum analyses by ultra-deep sequencing refers to the coverage (e.g. minimum number of reads) needed to ensure that a mutant at 1% or above is reliably detected. To address this question, we performed two theoretical studies*.* In the first one, we envisaged a huge repository of mutants at different frequencies (mimicking a viral quasispecies), and asked which was the range of frequencies at which a mutant (theoretically present at 1%) would be detected when the coverage varied between 500 and 10,000 reads (Additional file 12: Figure S6A). With a coverage of 500 reads, mutants present at 1% were sampled at quite variable frequencies (a range between no detection and up to 2.5%, depending on the confidence interval). An increase from 500 to 10,000 reads narrowed the varibility of mutant detection to around 1%. In a second experiment, we devised a huge repository of mutants at different frequencies including those expected to be artifacts, present at ≤0.5%. We asked which was the range of frequencies at which mutants at 1% and mutant artifacts at 0.5% were found with four sample sizes (1,000, 3,000, 6,000, and 10,000 reads), and at four confidence intervals (95%, 99%, 99.5% and 99.9%) (Additional file 12: Figure S6B). Coverages of 1,000 and 3,000 reads resulted in overlaps of the frequency range at which real mutants at 1% and artifact mutants at 0.5% were detected at all confidence intervals tested. However, when the number of reads was 10,000 no overlap between real and artifactual mutants was detected at confidence intervals from 95 to 99.5%. Thus, we define 10,000 as the minimum number of reads to detect mutants present at ≥1% in a reliable manner when the noise level is at 0.5%.

### Limit of detection of combined amino acid substitutions

Subsequent experiments were designed to control the sensitivity of mutant detection, and the degree of recombination during PCR amplifications. In order to mimic the presence of a minority mutant observed in natural samples, we mixed the reference (wt) viral HCV DNA and HCV DNA including mutations A7010C and G7073A (corresponding to amino acid substitutions N248H and E269K) at a 90:10 ratio. This mixture was subjected to four amplification protocols that compared different number of PCRs, and amplification conditions (Additional file 13: Figure S7). Each assay was performed in duplicate. Protocol 1 consisted in three PCRs (external PCR, internal PCR, and MID PCR) in which the concentration of the oligonucleotides was 0.4 μM, the elongation time 40 s, and the number of cycles 80. Protocol 2 included also three PCRs but the oligonucleotide concentration and the elongation time were increased to 1 μM and 60 s, respectively, and the number of cycles was decreased to 60. Protocols 3 and 4 consisted in two PCRs (external PCR, and MID), with PCR amplification conditions of protocol 1 and 2, respectively. Four types of molecules were expected in the sequence analysis: wt without mutations, a mutant clone encoding substitutions N248H and E269K, and two recombinant molecules N248H/wt, and wt/E269K. The four amplification products were sequenced using the platform 454-GS Junior, and the products of protocols 3 and 4 also with MiSeq. Protocols 1 and 2 resulted in the detection of recombinant genomes at a frequency of 3.55% for N248H/wt, and 2.44% for wt/E269K in protocol 1, and 3.71% for N248H/wt, and 2.04% for wt/E269K in protocol 2 (all values are the average of the two replicates). Protocols 3 and 4 (that included two PCRs) resulted in the detection of N248H/wt recombinant genomes at frequencies of 0.68% and 0.69% for protocols 3 and 4, respectively. Samples amplified using protocols 3 and 4 (including two PCRs) were also sequenced with MiSeq (Illumina). Recombinant genomes were detected at a frequency of 0.58% for N248H/wt, and 0.56% for wt/E269K in protocol 3, and 0.80% for N248H/wt, and 0.70% for wt/E269K in protocol 4. Taken together, amplification protocols based on two PCRs not only improved the frequency at which the double mutant was detected but also minimized the frequency of recombinant molecules to levels below 1%. These results established a frequency of 1% as conservative limit of haplotype detection.

### Ultra-deep sequencing data management

Due to the high variability of HCV in vivo the establishment of sequences that can be defined as reference sequences for each genotype and subtype is not trivial. Here, we have used the HCV sequence alignments retrieved from Los Alamos databank to define ten consensus sequences (one per each subtype) spanning amino acids 14 to 180 in NS3, 8 to 173 in NS5A, and 124 to 575 in NS5B which are the longest regions covered by our PCRs (Additional file 14: Figure S8). Each consensus sequence was determined by the most frequent amino acid at each position. Since previously characterized RAS are comprised within amino acid 36 to 175 in NS3, 24 to 93 in NS5A, and 159 to 561 in NS5B [9, 10, 14], their detection is included in our analysis.

**Fig. 4:**
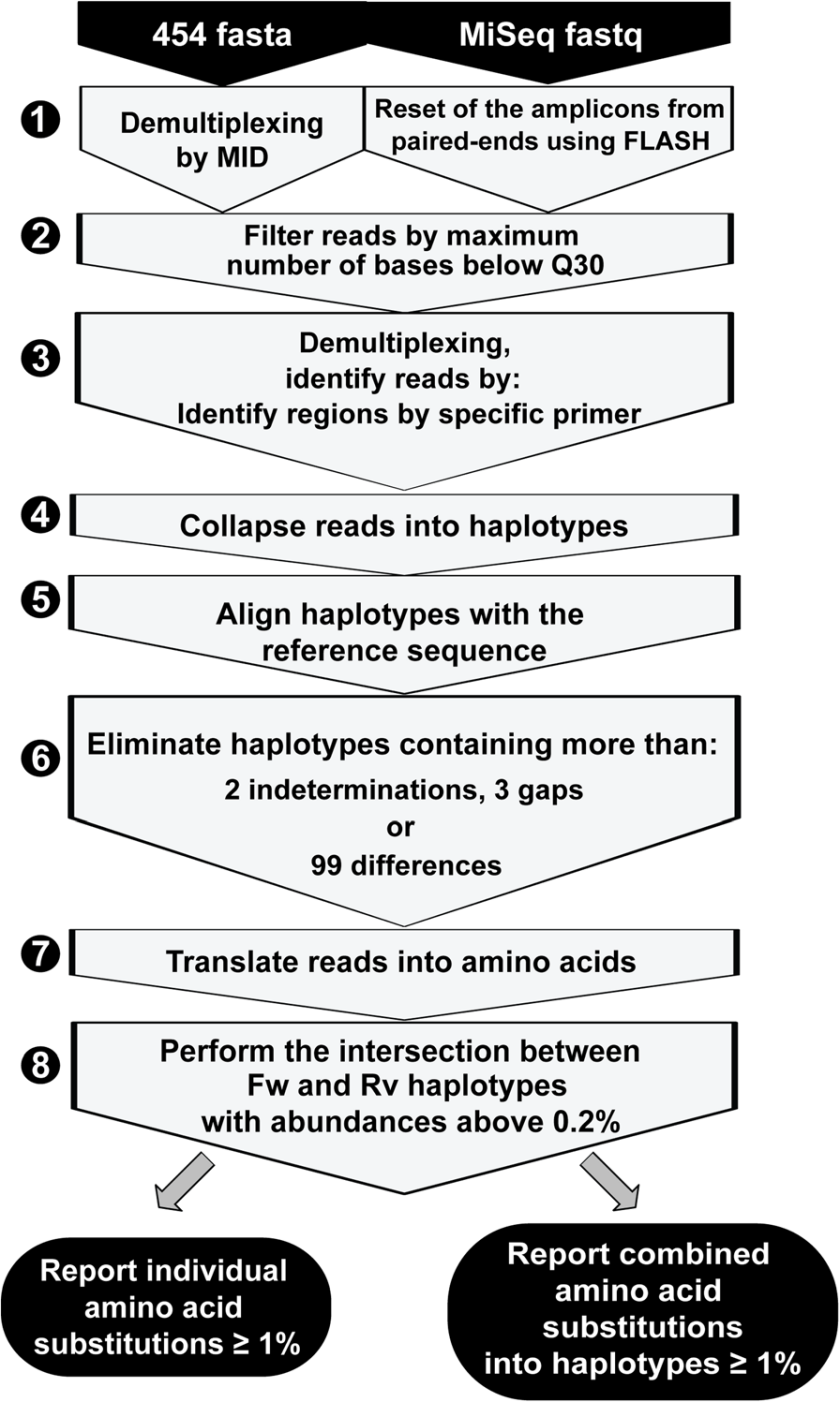
Bioinformatic pipeline to obtain the report of amino acid substitutions. Massive sequencing was performed using 454/GS-Junior (Roche), and MiSeq (Illumina) platforms. Each fasta file was subjected to the eight sequential steps depicted in the irregular pentagons with gray background; steps are explained in the text. The outcome is a report of amino acid substitutions with abundances ≥1% compared with the reference sequence. The report is described in two ways: individual amino acid substitutions present at a given frequency (filled panel at the bottom left), and combined amino acid susbtitutions into haplotypes (each at a given frequency) (filled panel at the bottom right)

We have developed a bioinformatic *haplotype-centric* procedure to exclude full reads that do not meet minimum quality requirements (Fig. 4). Raw data were obtained from GS-Junior 454 (454 fasta), and from MiSeq (fastq). The first step includes demultiplexing by MID (in the case of 454 GS-Junior), and the overlap of paired-end reads using FLASH (in the case of MiSeq). The FLASH parameters were established as a minimum overlap between R1 and R2 of 20 bp with a maximum of 10% differences. Reads not fulfilling this requirement were discarded. The yield of this process ranged from 60 to 80% in all experiments. The quality profile of the overlapped reads was substantially better than the original reads, with a 5% lower quantile consistently above Q30, and just slightly below Q30 for the ~ 50 bp in the center. A filter step on the FLASH reads was added to improve both sensitivity and specificity, the threshold was stablished by experiments with spikes and controls, finally excluding all reads with more than 5% bp with Phred score below Q30. The yield of this filter is very sensitive to the general quality of the sequencing run, and has been found to be in the range of 70–90%. The third step was a demultiplexing by specific oligonucleotides to obtain a fasta file by region. Reads were then collapsed into haplotypes with corresponding frequencies. Haplotypes were aligned with the reference sequence, and haplotypes containing more than two indeterminations, three gaps or 99 differences were also discarded. Accepted indeterminations and gaps were repaired as per the contents of the dominant haplotype. The yield of this process was above 90% in all cases. Then, reads were translated to amino acids, and the intersection between forward and reverse haplotypes with abundances ≥0.2% was performed. The yield of this process has been found to be in the range of 45–60%. Based on the controls reported in Additional file 11: Figure S5 and Additional file 13: Figure S7, all amino acid variants by site or haplotype at 1% or above are reported. The global yield is 15–30% of raw reads.

### Testing the pipeline: Ultra-deep sequencing of viral RNA from HCV-infected patients

**Fig. 5:**
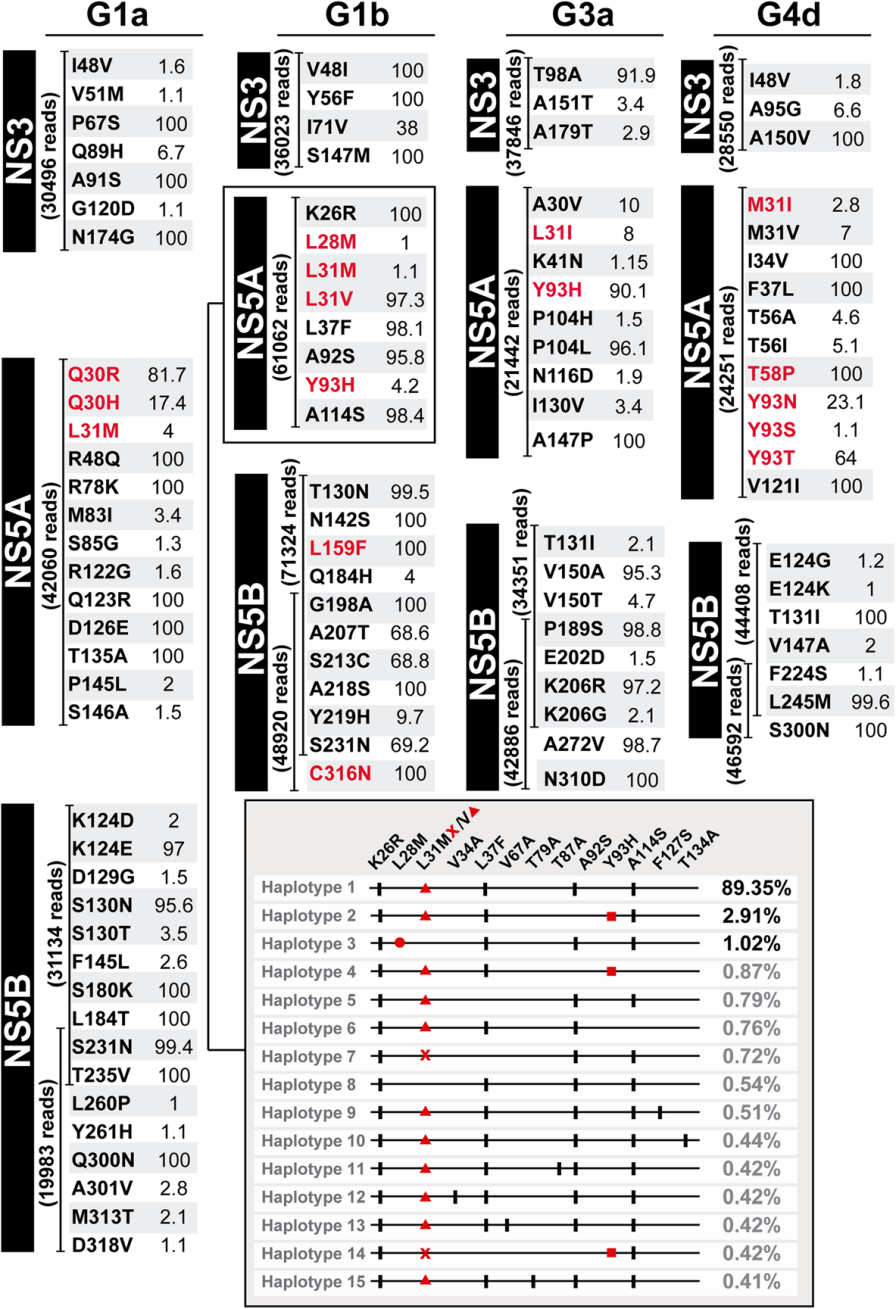
Ultra-deep sequencing of four HCV samples derived from patients who failed directly-acting antiviral agents (DAAs) therapies. Viral RNAs from four HCV-infected patients, corresponding to G1a, G1b, G3a, and G4d (top headings) were used to amplify NS3- [PCR 1.1 (533 bp) in Fig. 2], NS5A- [PCR 2.1 (477 bp) in Fig. 2], and NS5B-coding regions [PCRs 3.1 (478 bp) and 3.2 (483 bp) in Fig. 2] using MiSeq platform. For each amplicon the number of cleaned reads (given in parenthesis), the list of amino acid substitutions, and their frequencies (percentage given following each amino acid substitution) are indicated. Failure occurred after the following treatments: ledipasvir/sofosbuvir/ribavirin (12 weeks), ledipasvir/sofosbuvir (12 weeks), daclatasvir/sofosbuvir/ribavirin (12 weeks), ledipasvir/sofosbuvir/ribavirin (12 weeks) for G1a, 1b, 3a, and 4d HCV-infected patients, respectively. Viral load was 6.9 × 10^4^ IU/ml, 1.4 × 10^6^ IU/ml, 7.6 × 10^5^ IU/ml, and 6.3 × 10^4^ IU/ml for G1a, 1b, 3a, and 4d HCV-infected patients, respectively. Substitutions written in red means that they are known to confer resistance to DAAs included in the treatment combinations. Distribution of haplotypes in the NS5A-coding region for the G1b patient is depicted in the box at the bottom to explain the difference between the frequency of an individual amino acid substitution (independently of the haplotypes that contain it) or the frequency of an haplotype that includes the same substitution in its constellation of substitutions

To validate the subtype-specific deep-sequencing procedure described in previous sections for its application in vivo, the NS3-, NS5A-, and NS5B-coding regions of four patient samples (four amplicons per patient) belonging to subtypes 1a, 1b, 3a, and 4d that failed DAA-antiviral treatments were sequenced using the MiSeq platform (Fig. 5). An average 33,229, 37,204, 45,304, and 39,595 reads were generated for NS3 1.1, NS5A 2.1, NS5B 3.1, and NS5B 3.2 amplicons, respectively. Frequencies of individual substitutions ranged between 1 and 100%, and treatment failures were explained by the presence of RAS in the genomic region encoding the protein targeted by the DAAs. Thus, G1a, 3a, and 4d HCV-infected patients selected RAS only in NS5A-coding regions, whereas in the G1b HCV-infected patient RAS both in NS5A- and NS5B-coding regions were selected. To illustrate how amino acid substitutions can be quantified individually or combined in the same read, ultra-deep sequencing results of NS5A of the G1b HCV-infected patient were also analyzed by haplotypes. Substitution L28 M was found only in one haplotype which means that its frequency (1.02%) is similar either considered as an individual substitution or combined with other substitutions in the same haplotype. In contrast, L31 M has a frequency above 1% as individual RAS (the sum of haplotypes 7 and 14 in Fig. 5), but the frequency of the individual haplotypes is below 1% (0.72% for haplotype 7, and 0.42% for haplotype 14). Thus, resistance to ledipasvir that a priori could be assigned to replacements L31 M and Y93H (both present in haplotype 14 in Fig. 5), in our computation will be attributed to L31 M (that reaches a frequency above 1% when quantitated individually), but not to the combination of L31 M and Y93H because their frequency as a combination is below the cut-off value. In the same position, L31 V was found combined with Y93H in haplotype 2 whose frequency (2.91%) was above the limit of detection (Fig. 5). L31 V and Y93H confer 15-, and 12-fold increased resistance to daclatasvir compared to wild-type replicon, respectively, when they are alone, but the mean-fold change in resistance increases to 5425 when combined in the same amplicon [14].

To further study the dynamics of amino acid substitutions in a prolonged infection with HCV, serum samples derived from a G1a HCV-infected patient that had undergone three sequential rounds of DAA-based treatments were analyzed (Additional file 15: Figure S9). Viral load was measured during 116 weeks. Viral RNA at four time points (pre- and post-treatment with ledipasvir/sofosbuvir and daclatasvir/sofosbuvir) was used to amplify NS3-, NS5A, and NS5B-coding regions using G1a-specific oligonucleotides. The results indicated that viral failure upon the first treatment with ledipasvir/sofosbuvir was accompanied with selection of substitution Y93C in NS5A, known to confer resistance to NS5A inhibitors [9, 10, 14]. This susbtitution was maintained at 100% frequency before starting the second treatment which included daclatasvir to which Y93C also confers resistance. Interestingly, two additional RAS M28 T and L31 M were also co-selected with Y93C (Additional file 15: Figure S9), that may also contribute to resistance to NS5A inhibitors. Thus, the analysis of HCV samples from infected patients validates the experimental bioinformatic pipeline for HCV characterization. Analysis of RAS using our subtype-specific analytical procedure before DAA treatment onset would help to define a salvage treatment that avoids a new failure.

## Discussion

An estimate of the nucleotide sequence heterogeneity of HCV when it circulates in infected patients can now be approached using ultra-deep sequencing technologies even knowing that only a part of the real mutant repertoire at any given time will be analyzed. We have developed a procedure for a robust HCV viral quasispecies analysis by ultra-deep sequencing taking into account that the degree of oligonucleotide match for hybridization to viral RNA is limited by the high diversification of HCV into genotypes and subtypes in clinical samples. We propose an oligonucleotide design based on the comparison of ensembles of sequences representative of each subtype. Using this approach, we defined positions that were discriminatory for a specific subtype to generate a total of 280 oligonucleotides for three coding regions that have been associated with drug resistance (NS3, NS5A, and NS5B) in ten prevalent subtypes worldwide. This design can be extended to additional subtypes, should they arise in the course of HCV expansions [37, 38]. Our sequencing strategy requires knowing the subtype (or mixture of subtypes) present in the infected patient in order to choose the appropriate oligonucleotide pairs. Since current antiviral therapies are still largely genotype-dependent, an accurate determination of the genotype and subtype is mandatory in clinical settings for treatment planning. Depending on the genotypic assay used, a non-negligible percentage of patients are misclassified regarding genotype and subtype [39–41]. High resolution NGS technologies ensure an accurate determination of HCV subtypes [34, 42]. Thus, we suggest a two-step NGS procedure based on an initial HCV subtyping coupled with a second NS3-, NS5A-, and NS5B-coding region RAS determination using subtype-specific oligonucleotides. This approach fits EASL guidelines that recommend retreatment of HCV-infected patients —who failed one or more antiviral therapies— with a new DAA combination based on RAS determination. In our view, RAS analysis should include NS3, NS5A and NS5B, the target of most current DAAs. Analyses of RAS at basal samples in DAA-naïve patients is not standardized yet due to the success rates of DAA therapies. However, it would be advisable to implement them, especially when a higher than average NS5B RAS prevalence is present in some treatment-naïve cohorts [16].

Other approaches for general HCV sequencing have been proposed. Hedskog et al., described a genotype- and subtype-independent HCV amplification strategy coupled with NGS but based on the use of random oligonucleotides [43]. This subtype-independent method would be especially advantageous to amplify and sequence unknown HCV genotypes and subtypes. Bull et al. described a near full-length amplification adequate for the all major six genotypes [44]. A comparison of several NGS technologies revealed their utility for diagnostic and clinical assessment [45]. The development of a subtype-specific ultra-deep sequencing analysis can be seen as a limitation due to the inherent complexity of the oligonucleotide primer design. Also, the small number of samples analyzed here should be increased with additional cohorts. However, the development of standardized RAS sequencing methods should be a priority [46]. In our procedure we have designed amplifications able to discriminate between subtypes that may disclose hidden subpopulations that would be difficult to amplify using suboptimal oligonucleotides. When new amplicons or sequencing platforms are used, sources of potential sequencing error should be controlled in order to distinguish genuine sequence variation from technical mistakes. Analyses of clonal sequences in our procedure established that individual mutations detected at < 1% should be excluded. The need of amplifying viral RNA molecules by RT-PCR raises also the problem of potential artificial recombinants produced during PCRs [29]. Several factors have been described that enable a substantial decrease of PCR-associated recombination, including increases in oligonucleotide primer concentrations and elongation times, and a reduction in the number of PCR cycles and of initial template concentrations [28, 47]. Under our experimental conditions, only the reduction in the number of PCRs (two instead of three) decreased the frequency of artifactual chimeras to < 1%.

Our approach can be easily adapted to other HCV genomic regions in case new DAAs with other viral target are developed, or to additional HCV genotypes and subtypes that are likely to arise in view of the progressive diversification of the virus, as judged from its evolution over the last decade (compare [4] with [48]). Our approach should also broaden its application given the increasing evidence of the presence of RAS in treatment-naïve patients [15–17]. Finally, an analogous pipeline of sequence-based oligonucleotide design and deep-sequencing scrutiny of prominent mutations can be applied to emergent viral diseases associated with highly variable RNA viruses.

## Conclusions

We have established a robust pipeline for the amplification and next-generation sequencing of samples derived from HCV-infected patients. This procedure is based on the design of subtype-specific oligonucleotide primers that amplify HCV genomic regions in a subtype-specific manner, as validated with patient’s viral samples. Cut-off values for reliable quantification of mutations associated with drug resistance have been established that may guide treatment planning. The method can also be adapted to sequence new HCV subtypes, as they arise in the course of HCV diversification.

## Additional files

Additional file 1: **Table S1.** Reference accession numbers of sequences retrieved from Los Alamos database to design subtype-specific oligonucleotides. (PDF 11 kb)

Additional file 2: **Table S2.** Oligonucleotides used to perform the site-directed mutagenesis, qRT-PCR, control of basal amino acid sequencing error, and control of PCR recombination. (PDF 97 kb)

Additional file 3: **Table S3.** Experimental samples and sequencing platforms used throughout this study. (PDF 181 kb)

Additional file 4: **Figure S1.** Subtype-specific oligonucleotides designed to sequence the NS3-coding region. Residue numbering is according to the reference strain AF009606. Positions in red are conserved among the different subtypes, and positions with different colors are discriminatory of a specific subtype (color codes given in the left column at each panel). Discriminatory positions for genotype are highlighted in pink. (PDF 1007 kb)

Additional file 5: **Figure S2.** Subtype-specific oligonucleotides designed to sequence the NS5A-coding region. Residue numbering is according to the reference strain AF009606. Positions in red are conserved among the different subtypes, and positions with different colors are discriminatory of a specific subtype (color codes given in the left column at each panel). Discriminatory positions for genotype are highlighted in pink. (PDF 1014 kb)

Additional file 6: **Figure S3.** Subtype-specific oligonucleotides designed to sequence the NS5B-coding region. Residue numbering is according to the reference strain AF009606. Positions in red are conserved among the different subtypes, and positions with different colors are discriminatory of a specific subtype (color codes given in the left column at each panel). Discriminatory positions for genotype are highlighted in pink. (PDF 2762 kb)

Additional file 7: **Table S4.** Subtype-specific oligonucleotides designed to sequence the NS3-coding region. (PDF 157 kb)

Additional file 8: **Table S5.** Subtype-specific oligonucleotides designed to sequence the NS5A-coding region. (PDF 158 kb)

Additional file 9: **Table S6.** Subtype-specific oligonucleotides designed to sequence the NS5B-coding region. (PDF 250 kb)

Additional file 10: **Figure S4.** Comparative analysis using inter- and intra- sequencing platforms. Viral RNAs of two G1b HCV-infected patients (termed 8101 and JRR) at treatment (simeprevir/sofosbuvir/ribavirin) failure were used to amplify NS3- (PCR 1.1 in Fig. 2), NS5A- (PCR 2.1 in Fig. 2), and NS5B-coding regions (PCRs 3.1 and 3.2 in Fig. 2), following the procedure described in Materials ans Methods. Amplicons derived from 8101 viral RNA amplifications were sequenced in parallel using MiSeq and 454 GSJunior platforms (inter-sequencing platforms comparison). Amplicons derived from JRR viral RNA amplifications were sequenced twice in two different runs using MiSeq platform (MiSeq A and B) (intra-sequencing platform comparison). Percentage of common and unique haplotypes and reads between both runs at each comparison are represented. Scatter plots comparing the percentage of variants obtained according to the two runs of each amplicon are shown on the right. (PDF 415 kb)

Additional file 11: **Figure S5.** Control of basal error. A full-length HCV DNA encoding NS5A with amino acid substitutions N248 K, E269K and A346V was used as a template to determine the basal error of the amplification and sequencing process using 454 GS-Junior and Illumina MiSeq platforms. Due to restriction size of amplicon length, only N248 K and E269K were detected. Experiments were performed in triplicate. Haplotypes obtained after amplification and sequencing are numbered on the left of each replicate, and percentages of reads that include the indicated substitutions are shown on the right. Artifacts means mutations other than those encoding N248 K and E269K. Basal error average is the mean ± standard deviation of the haplotypes number 2, which are those including artifacts found at the highest frequency. (PDF 291 kb)

Additional file 12: **Figure S6.** Theoretical study to define the reliable coverage needed to detect a mutant present at 1% in a viral population. (A) Confidence intervals (CI) of the observed proportions (given in the abscissa) of a variant amino acid present at 1% frequency in a viral population, with coverages varying from 500 to 10,000 reads (given in the ordinate) according to the binomial law. Left: 95% CI; Right: 99% CI. (B) Effect of the coverage at four different CIs (indicated in ordinate) considering that true variants (in blue) are present at 1%, and artifact variants (in pink) at 0.5%. The abscissa gives the percentage at which the two classes of variants are observed. Note that at high read coverages the overlap between true and artifact variants is minimal. (PDF 1158 kb)

Additional file 13: **Figure S7.** Control of PCR-based recombination. A full-length reference (wt) viral HCV DNA was mixed with HCV DNA with mutations A7010C and G7073A (corresponding to amino acid substitutions N248 K and E269K in NS5A) at a 90:10 ratio (depicted on the left of the top box). The total number of DNA molecules was 100,000. The mixture was used as a template to determine the degree of recombination after the amplification and sequencing process, following four different protocols (termed 1 to 4), and two next-generation sequencing platforms (454 GS-Junior and Illumina MiSeq). Protocol conditions are described in Materials and Methods. Top box depicts the four types of expected molecules; wt clone, mutant N248 K/E269K, and recombinant molecules N248 K/wt and wt/E269K. Each experiment was performed in duplicate (replicates 1 and 2), and the average frequency (%) of both replicates is shown on the right of each molecule; n.d. means not detected. The percentage of reads that include the indicated substitutions is represented for each haplotype and protocol in the three panels at the bottom. (PDF 399 kb)

Additional file 14: **Figure S8.** Definition of the reference sequence for the ten subtypes under study. Nucleotide numbering at the beginning and at the end of NS3, NS5A and NS5B indicates the length of each coding region. Amino acid number of each coding region is indicated between the arrows. Positions at which resistance-associated substitutions (RAS) have been described are shown above the coding regions, and are included in our analyses. Alignments include a reference sequence for each subtype (1a, 1b, 2a, 2b, 2c, 2j, 3a, 4a, 4d, 4f); amino acid numbering above the boxes is indicated. Each reference sequence is defined as the most frequent amino acid at each position of the sequence ensemble for each subtype described in Table S1. Nucleotide and amino acid numbering are based on the HCV strain H77 (GenBank accession AF009606). (PDF 849 kb)

Additional file 15: **Figure S9.** Ultra-deep sequencing of viral samples from an G1a HCV-infected patient subjected to sequential DAA treatments. First panel: Representation of the viral load [international units (IU)/ml] as a function of time (in weeks). A yellow background indicates period in which antiviral treatment was applied (LDV: ledipasvir; SOF: sofosbuvir; RBV: ribavirin; DCV: daclatasvir; SMV: simeprevir). Viral relapse ocurred after the first two treatments, and SVR (sustained virological response) was achieved after the third treatment. Viral RNAs from four sequential samples (termed 1 to 4, indicated with arrows) were used to amplify NS3- (PCR 1.1 in Fig. 2), NS5A- (PCR 2.1 in Fig. 2), and NS5B-coding regions (PCRs 3.1 and 3.2 in Fig. 2) using MiSeq platforms. Subsequent panels: For each coding region (NS3, NS5A, NS5B) variations in mutation frequencies (as percentage of individual mutations) are represented as mutational waves (mutations that increase or decrease in frequency, relative to the previous or subsequent sample analyzed), and in a heat map of frequencies (color boxed below the graphics). Substitutions written in red confer resistance to the DAAs included in the treatment combinations, or are described in the literature (European Association for the Study of the Liver. Electronic address, 2017; Sarrazin, [9]; Lontok et al., [14]). Asteriscs in the heat map of NS5A-coding region in samples at viral relapse 2 and 4 highlight RAS that confer resistance to ledipasvir and daclatasvir, respectively. Haplotypes and their percentages (proportion of reads that include the indicated substitutions) are depicted on the right of each panel. (PDF 1030 kb)

## Abbreviations

AASLD/IDSA: American Association for the Study of Liver Diseases/Infectious Diseases Society of America
DAA: Direct-acting Antiviral Agent
EASL: European Association for the Study of the Liver
FLASH: Fast Length Adjustment of SHort reads
HCV: Hepatitis C Virus
ICTV: International Committee on Taxonomy of Viruses
MID: Multiplex IDentifier
NGS: Next-Generation Sequencing
PCR: Polymerase Chain Reaction
RAS: Resistance-Associated Substitution
RT-PCR: Reverse Transcriptase-Polymerase Chain Reaction
SVR: Sustained Virological Response
