# Supplementary material for: Pipeline for specific subtype amplification and drug resistance detection in hepatitis C virus
Source: BMC Infect Dis. 2018 Sep 3;18:446. doi: 10.1186/s12879-018-3356-6 (PMC6122477; doi:10.1186/s12879-018-3356-6)
Supplement: Supplementary file 1 — Table S1. Reference accession numbers of sequences retrieved from Los Alamos database to design subtype-specific oligonucleotides. (PDF 11 kb) [file 12879_2018_3356_MOESM1_ESM.pdf]

Table S1. Reference accession numbers of sequences retrieved from Los Alamos database to design subtype-specific oligonucleotides.

Subtype 1a: AB520610, AF009606, AF011751 - AF011753, AF271632, AF511949, AF511950, AJ278830, EF407411 - EF407415, EF407417 - EF407419, EF407421 - EF407423, EF407425, EF407427, EF407428, EF407431 - EF407447, EF407449 - EF407457, EF621489, EU155214 - EU155216, EU155233, EU155236 - EU155245, EU155249 - EU155252, EU155265 - EU155278, EU155282 - EU155288, EU155291, EU155293, EU155294, EU155296, EU155297, EU155299, EU155309, EU155311, EU155313, EU155314, EU155319 - EU155323, EU155338 - EU155355, EU155378, EU155379, EU234064, EU234065, EU239715, EU239716, EU250017, EU255927 - EU255958, EU255963 - EU255971, EU255973 - EU255992, EU255994 - EU255999, EU256002 - EU256024, EU256026, EU256028 - EU256034, EU256036 - EU256044, EU256047 - EU256053, EU256055 - EU256058, EU256060, EU256067, EU256068, EU256070 - EU256074, EU256087, EU256094, EU256095, EU256097, EU256105 - EU256107, EU260396, EU362876, EU362877, EU362879, EU362880, EU362882, EU362884 - EU362887, EU362891 - EU362898, EU362901, EU482831, EU482832, EU482834 - EU482838, EU482840 - EU482848, EU482852 - EU482858, EU482861 - EU482873, EU482878, EU482882, EU482884, EU482887, EU482889, EU529676 - EU529681, EU569722, EU569723, EU595697 - EU595699, EU660383 - EU660385, EU660387, EU687193 - EU687195, EU781746 - EU781803, EU781805 - EU781822, EU862824, EU862827, EU862828, EU862830 - EU862832, EU862834, EU862839 - EU862841, FJ024087, FJ024274 - FJ024276, FJ024278, FJ024280 - FJ024282, FJ181999 - FJ182001, FJ205867 - FJ205869, FJ390394, FJ390395, FJ390399, FJ410172, GQ149768, JQ914271, JQ914272, JX463525 - JX463530, JX463532 - JX463538, JX463541 - JX463545, JX463551 - JX463615, JX463617 - JX463622, JX463624 - JX463626, JX463628 - JX463633, JX463635 - JX463638, KC844049, M62321, M67463, NC_004102.

Subtype 1b: AB049087 - AB049096, AB049098 - AB049101, AB080299, AB154177 - AB154206, AB191333, AB249644, AB426117, AB429050, AB435162, AB442219 - AB442222, AB691953, AB779562, AB779679, AF054247 - AF054255, AF054257 - AF054259, AF165045 - AF165064, AF176573, AF207752 - AF207758, AF207760 - AF207774, AF208024, AF313916, AF333324, AF356827, AF483269, AJ000009, AJ132996, AJ132997, AJ238799, AJ238800, AY045702, AY587016, AY587844, D10750, D10934, D11168, D11355, D13558, D14484, D30613, D45172, D50480 - D50485, D63857, D85516, D89815, D89872, D90208, DQ071885, EF032892 - EF032894, EF407458 - EF407504, EU155217 - EU155232, EU155235, EU155253 - EU155264, EU155279 - EU155281, EU155300 - EU155308, EU155315 - EU155318, EU155324 - EU155337, EU155356 - EU155377, EU155381, EU155382, EU234061, EU234062, EU239714, EU255960 - EU255962, EU256000, EU256001, EU256045, EU256059, EU256061, EU256062, EU256064 - EU256066, EU256075 - EU256085, EU256088 - EU256092, EU256098 - EU256103, EU482833, EU482839, EU482849, EU482859, EU482860, EU482874, EU482875, EU482877, EU482879 - EU482881,

EU482883, EU482885, EU482886, EU482888, EU529682, EU660386, EU660388, EU781825 - EU781832, EU862835, EU862837, FJ024086, FJ024277, FJ024279, FJ390396 - FJ390398, FJ478453, FN435993, GU133617 - GU451224, HQ110091, HQ639937, HQ639940, HQ639946, HQ639947, HQ719473, HQ912956 - HQ912959, JN120912, KC439481 - KC439527, KC844051, KC844052, L02836, M58335, M84754, M96362, U01214, U16362, U45476, X61596.

Subtype 2a: AB047639 - AB047645, AB690460, AB690461, AF169002 - AF169005, AF177036, AF238481 - AF238485, AY746460, D00944, HQ639938, HQ639939, HQ639943 - HQ639945, JX014307, KC844043, KC967476, KF676351, KF676352, KF700370, NC_009823.

Subtype 2b: AB030907, AB559564, AB661373, AB661374, AB661376 - AB661378, AB661380 - AB661386, AB661389 - AB661393, AB661395 - AB661397, AB661399 - AB661403, AB661405 - AB661407, AB661409 - AB661422, AB661424 - AB661431, AF238486, AY232730 - AY232749, D10988, DQ430815, DQ430817, JQ745651, KC197226, KC844048.

Subtype 2c: D50409, JX227950, JX227951, JX227965, JX227966, KC197227, KC197228.

Subtype 2j: HM777358, HM777359, JF735113, KC197232, KC197233.

Subtype 3a: AB691595, AB691596, AB792683, AF046866, AY956467, D17763, D28917, DQ430819, DQ430820, DQ437509, GQ275355, GQ356200 - GQ356217, GU814263, HQ639941, HQ639942, HQ912953, JN714194, JQ717254 - JQ717260, KC844041, KF035123 - KF035127, NC_009824, X76918.

Subtype 4a: AB795432, DQ418782 - DQ418784, DQ418787 - DQ418789, DQ516084, DQ988073 - DQ988079, GU814265, NC_009825, Y11604.

Subtype 4d: DQ418786, DQ516083, EU392172, FJ462437, KC844045.

Subtype 4f: EF589160, EF589161, EU392169, EU392170, EU392174, EU392175.
