# Supplementary material for: Pipeline for specific subtype amplification and drug resistance detection in hepatitis C virus
Source: BMC Infect Dis. 2018 Sep 3;18:446. doi: 10.1186/s12879-018-3356-6 (PMC6122477; doi:10.1186/s12879-018-3356-6)
Supplement: Supplementary file 2 — Table S2. Oligonucleotides used to perform the site-directed mutagenesis, qRT-PCR, control of basal amino acid sequencing error, and control of PCR recombination. (PDF 97 kb) [file 12879_2018_3356_MOESM2_ESM.pdf]

Table S2. Oligonucleotides used to perform the site-directed mutagenesis, qRT-PCR, control of basal amino acid sequencing error, and control of PCR recombination.

Primer Name	Sense	Sequence (5'-3')	Position[a]
Jc1-NS5A-F2	Fw	ACTACCTTCTCCAGAGTTTTTC	6697
Bch-0-3[b]	Rv	AGCAGGTTGGCATCGACCATGTCCACGTCA TAGGTATGGCTGTGGGTGGTGCAGGTGG	7047
Bch-0-4[c]	Fw	TGCCAACCTGCTCATGGAGGGCGGTGTGGCT CAGACAAAACCTGAGTCCAGGGTGCCCGT	7036
Bch-0-21[d]	Rv	AGGCGTCGGAACCTTCTTGG	7315
Bch-0-6[d]	Fw	AGAAGGTTCCGACGCCTCCCCCAAGGAGAC	7299
Jc1-NS5B-R1	Rv	TCTCGCAGACCCGGACGCCGAG	8180
HCV-5UTR-F2	Fw	TGAGGAACTACTGTCTTCACGCAGAAAG	47
HCV-5UTR-R2	Rv	TGCTCATGGTGCACGGTCTACGAG	347
Jc1NS5Au6521	Fw	CAGGGGACCTTTCCTATCAATTGCT	6521
Jc1NS5Ad7211	Rv	GCCGTGCCCAAGCCGGTA	7211
Jc1NS5AM13u6693[e]	Fw	GTTGTAAAACGACGGCCAGTGCCAACTACCTTCT CCAGAGTTTTT	6693
Jc1NS5AM13d7131[e]	Rv	CACAGGAAACAGCTATGACCCTCTCTTCCTC GGCCATTGGCT	7131

[a]According to JFH-1 accession number #AB047639
[b]Underlined nucleotides indicate modifications of the genomic sequence introduced to express NS5A with substitution N248H
[c]Underlined nucleotides indicate modifications of the genomic sequence introduced to express NS5A with substitution E269K
[d]Underlined nucleotides indicate modifications of the genomic sequence introduced to express NS5A with substitution A346V
[e]Underlined nucleotides indicate universal M13 oligonucleotide
