# Supplementary material for: Pipeline for specific subtype amplification and drug resistance detection in hepatitis C virus
Source: BMC Infect Dis. 2018 Sep 3;18:446. doi: 10.1186/s12879-018-3356-6 (PMC6122477; doi:10.1186/s12879-018-3356-6)
Supplement: Supplementary file 3 — Table S3. Experimental samples and sequencing platforms used throughout this study. (PDF 181 kb) [file 12879_2018_3356_MOESM3_ESM.pdf]

Table S3. Experimental samples and sequencing platforms used throughout this study.

Viral sample	Sequencing platform	Number of samples	Number of amplicons	Aim
HCV double mutant DNA	454 GS-Junior<br>MiSeq	3<br>3	3<br>3	Control of basal nucleotide sequencing error
Standard HCV DNA +HCV double mutant DNA at proportion of 10%	454 GS-Junior<br>MiSeq	8<br>4	8<br>4	Control of PCR recombination
Viral RNA from G1a, G1b, G3a, and G4d HCV-infected patients at DAA treatment failure	MiSeq	4	16	Validation of subtype-specific deep-sequencing procedure
Viral RNA from G1a HCV-infected patient subjected to sequential DAA treatments	MiSeq	4	16	Validation of subtype-specific deep-sequencing procedure
Viral RNA from G1b HCV-infected patient at DAA treatment failure	454 GS-Junior<br>MiSeq	1<br>1	4<br>4	Comparative analysis using inter-sequencing platforms
Viral RNA from G1b HCV-infected patient at DAA treatment failure	MiSeq	1	4	Comparative analysis using intra-sequencing platforms
