# Supplementary material for: Pipeline for specific subtype amplification and drug resistance detection in hepatitis C virus
Source: BMC Infect Dis. 2018 Sep 3;18:446. doi: 10.1186/s12879-018-3356-6 (PMC6122477; doi:10.1186/s12879-018-3356-6)
Supplement: Supplementary file 4 — Figure S1. Subtype-specific oligonucleotides designed to sequence the NS3-coding region. Residue numbering is according to the reference strain AF009606. Positions in red are conserved among the different subtypes, and positions with different colors are discriminatory of a specific subtype (color codes given in the left column at each panel). Discriminatory positions for genotype are highlighted in pink. (PDF 1007 kb) [file 12879_2018_3356_MOESM4_ESM.pdf]

Figure S1

RT-PCR NS3 (1)

(5'- 3') (Forward)

Subtypes	3384	3385	3386	3387	3388	3389	3390	3391	3392	3393	3394	3395	3396	3397	3398	3399	3400	3401	3402	3403	3404	3405	3406	3407	3408	3409	3410	3411	3412	3413	3414	3415
1a								G	A	A	T	G	G	T	C	T	C	C	A	A	G	G	G	G	T	G	G	A	G	G	T	
1b											T	T	G	A	R	G	G	G	C	A	G	G	G	G	T	G	G	C	G	R	C	T
2a				G	A	T	G	G	C	T	A	C	A	C	C	T	C	Y	A	A	G	G	G	G	T	G	G	A				
2b								G	C	T	A	C	A	C	C	T	C	G	A	A	G	G	G	G	T	G	G	A	A	G	C	T
2c	G	C	T	G	A	T	G	G	A	T	A	Y	A	C	C	T	C	Y	A	A	R	G	G	T	T	G	G	A				
2j				G	A	C	G	A	G	T	A	C	G	T	C	T	C	C	A	R	G	G	G	R	T	G	G	A				
3a				G	A	T	G	A	Y	T	A	T	C	G	G	G	A	G	A	T	G	G	G	T	T	G	G	C				
4a								C	A	G	A	A	A	C	A	T	C	M	A	A	G	G	G	G	T	G	G	A	G	A	C	T
4d								C	G	C	A	C	R	C	Y	A	T	R	A	A	G	G	G	G	T	G	G	A				
4f									T	G	A	G	G	T	G	T	C	R	G	G	G	G	G	G	T	G	G	A				

(5'- 3') (Reverse)

Subtypes	4007	4006	4005	4004	4003	4002	4001	4000	3999	3998	3997	3996	3995	3994	3993	3992	3991	3990	3989	3988	3987	3986	3985	3984	3983	3982	3981	3980	3979
1a				C	T	G	G	G	G	C	A	C	T	G	C	T	G	G	T	G	G	R	G	A					
1b	T	G	T	C	T	G	C	G	G	T	A	C	R	G	C	C	G	G	R	G	G								
2a	G	G	T	C	T	G	G	G	G	C	A	C	A	G	C	Y	G	G	T	G	G								
2b				C	T	G	G	G	G	C	A	C	A	G	C	T	G	G	T	G	G	Y	G	T					
2c	G	G	T	C	T	G	G	G	G	C	A	C	A	G	C	T	G	G	A	G	G								
2j			T	C	T	G	G	G	G	C	A	C	A	G	C	T	G	G	R	G	G	C	G	T					
3a				C	T	G	T	G	G	R	A	C	R	G	C	A	G	G	A	G	G	A	G	T	T	G	A	A	T
4a				C	T	G	R	G	G	C	A	C	Y	G	C	D	G	G	G	G	G	T	G	T					
4d				C	T	G	G	G	G	C	A	C	G	G	C	A	G	G	A	G	G	A	G	T					
4f				C	T	G	G	G	G	C	A	C	A	G	C	A	G	G	A	G	G	C	G	T					

PCR NS3 (1.1)

(5'- 3') (Forward)

Subtypes	3490	3491	3492	3493	3494	3495	3496	3497	3498	3499	3500	3501	3502	3503	3504	3505	3506	3507	3508	3509	3510	3511	3512
1a	G	G	G	A	C	A	A	A	A	A	C	C	A	R	G	T	G	G	A	G	G	G	T
1b	G	G	G	A	C	A	A	T	A	A	C	C	A	G	G	T	C	G	A	G	G	G	G
2a	G	Y	G	A	C	A	R	G	A	C	A	G	A	A	C	A	G	G	C	C	G	G	G
2b	G	C	G	A	C	A	A	A	A	A	T	G	A	G	C	A	G	G	C	C	G	G	G
2c	G	Y	G	A	C	A	A	G	A	C	R	G	A	M	C	A	G	G	C	T	G	G	G
2j	G	C	G	A	Y	A	A	A	A	C	R	G	A	G	T	G	T	G	C	C	G	G	A
3a	G	G	G	A	Y	A	A	G	A	A	Y	G	T	G	G	T	G	A	C	C	G	G	T
4a	G	G	G	A	C	A	C	C	A	A	T	G	A	R	A	A	T	T	G	T	G	G	T
4d	G	A	G	A	C	A	C	C	A	A	C	G	A	G	A	A	C	T	G	C	G	G	T
4f	G	G	G	A	Y	A	C	T	A	A	Y	G	A	G	A	A	Y	T	G	T	G	G	T

(5'- 3') (Reverse)

Subtypes	3982	3981	3980	3979	3978	3977	3976	3975	3974	3973	3972	3971	3970	3969	3968	3967	3966	3965	3964	3963	3962	3961	3960	3959	3958
1a	G	A	G	T	T	G	T	C	Y	G	T	G	A	A	C	A	C	C	G	G	G	G	A	C	C
1b	G	A	R	T	T	G	T	C	Y	G	T	G	A	A	G	A	C	C	G	G	R	G	A	C	C
2a	C	T	G	T	T	G	T	C	A	C	T	R	A	A	G	G	T	G	G	G	A	G	A	C	C
2b	C	T	G	T	T	G	T	C	A	G	A	G	A	A	A	C	T	G	G	G	C	G	W	C	C
2c	C	T	G	T	T	G	T	C	A	G	T	R	A	A	G	Y	T	T	G	G	A	G	A	C	C
2j	C	T	G	T	T	G	T	C	A	G	T	G	A	A	R	C	T	G	G	G	G	G	A	C	C
3a	G	A	A	T	T	G	T	C	A	G	A	G	A	A	R	G	A	T	G	G	A	G	A	C	C
4a	G	A	R	T	T	G	T	C	A	G	T	G	A	A	C	A	C	T	G	G	T	G	A	T	C
4d	G	A	A	T	T	G	T	C	A	G	A	G	A	A	T	A	C	T	G	G	R	G	A	C	C
4f	G	A	R	T	T	G	T	C	A	G	A	R	A	A	G	A	C	C	G	G	G	G	A	T	C

PCR NS3 (1.2)

(5'- 3') (Forward)

Subtypes	3438	3439	3440	3441	3442	3443	3444	3445	3446	3447	3448	3449	3450	3451	3452	3453	3454	3455	3456	3457	3458	3459	3460	3461
1a	G	C	C	C	A	G	C	A	G	A	C	R	A	G	G	G	G	C	C	T				
1b		C	C	C	A	A	C	A	G	A	C	G	C	G	G	G	G	C	C	T	A	C	T	T
2a	G	C	C	C	A	G	C	A	R	A	C	A	C	G	A	G	G	Y	C	T				
2b	A	C	Y	C	A	G	C	A	G	A	C	T	C	G	T	G	G	Y	C	T	C	C	T	
2c	G	C	C	C	A	G	C	A	A	A	C	C	C	G	A	G	G	K	C	T				
2j	G	C	T	C	A	G	C	A	G	A	C	C	C	G	T	G	G	C	C	T				
3a	G	C	C	C	A	G	C	A	A	A	C	Y	A	G	R	G	G	C	C	T	T			
4a		C	G	C	A	G	C	A	G	A	C	C	C	G	C	G	G	C	T	T				
4d		C	G	C	A	G	C	A	G	A	C	C	C	G	A	G	G	G	A	T				
4f	G	C	G	C	A	G	C	A	A	A	C	T	C	G	A	G	G	C	T	T				

(5'- 3') (Reverse)

Subtypes	3988	3987	3986	3985	3984	3983	3982	3981	3980	3979	3978	3977	3976	3975	3974	3973	3972	3971	3970	3969	3968	3967	3966	3965	3964	3963	3962	3961	3960	3959	3958	3957
1a							G	A	G	T	T	G	T	C	Y	G	T	G	A	A	C	A	C	C	G	G	G	G	A			
1b							G	A	R	T	T	G	T	C	Y	G	T	G	A	A	G	A	C	C	G	G	R	G	A	C	C	
2a						G	C	T	G	T	T	G	T	C	A	C	T	R	A	A	G	G	T	G	G	G	R	G	A			
2b				G	T	R	C	T	G	T	T	G	T	C	A	G	A	G	A	A	A	C	T	G	G	G	C	G				
2c							C	T	G	T	T	G	T	C	A	G	T	R	A	A	R	Y	T	T	G	G	A	G	A	C	C	T
2j						G	C	T	G	T	T	G	T	C	A	G	T	G	A	A	R	C	T	G	G	G	G	G	A			
3a	G	G	A	G	T	T	G	A	A	T	T	G	T	C	A	G	A	G	A	A	R	G	A	T	G	G	A	G	A			
4a				G	T	T	G	A	R	T	T	G	T	C	A	G	T	G	A	A	C	A	C	T	G	G	T	G	A			
4d				G	T	G	G	A	A	T	T	G	T	C	A	G	A	G	A	A	T	A	C	T	G	G	A	G	A			
4f							G	A	R	T	T	G	T	C	A	G	A	A	A	A	G	A	C	C	G	G	G	G	A			

Figure S1. Subtype-specific oligonucleotides designed to sequence the NS3-coding region. Residue numbering is according to the reference strain AF009606. Positions in red are conserved among the different subtypes, and positions with different colors are discriminatory of a specific subtype (color codes given in the left column at each panel). Discriminatory positions for genotype are highlighted in pink.
