# Supplementary material for: Pipeline for specific subtype amplification and drug resistance detection in hepatitis C virus
Source: BMC Infect Dis. 2018 Sep 3;18:446. doi: 10.1186/s12879-018-3356-6 (PMC6122477; doi:10.1186/s12879-018-3356-6)
Supplement: Supplementary file 6 — Figure S3. Subtype-specific oligonucleotides designed to sequence the NS5B-coding region. Residue numbering is according to the reference strain AF009606. Positions in red are conserved among the different subtypes, and positions with different colors are discriminatory of a specific subtype (color codes given in the left column at each panel). Discriminatory positions for genotype are highlighted in pink. (PDF 2762 kb) [file 12879_2018_3356_MOESM6_ESM.pdf]

Figure S3

RT-PCR NS5B (3)

(5'- 3') (Forward)

Subtypes	7952	7953	7954	7955	7956	7957	7958	7959	7960	7961	7962	7963	7964	7965	7966	7967	7968	7969	7970
1a	C	C	A	C	A	T	C	A	A	C	T	C	C	G	T	G	T	G	G
1b	C	C	A	C	A	T	C	C	R	C	T	C	C	G	T	G	T	G	G
2a	C	C	A	C	A	T	C	A	A	G	T	C	C	G	T	G	T	G	G
2b	C	C	A	C	A	T	C	C	G	G	T	C	C	G	T	G	T	G	G
2c	C	C	A	C	A	T	C	A	A	G	T	C	C	G	T	G	T	G	G
2j	C	C	A	C	A	T	C	M	A	G	T	C	C	G	T	G	T	G	G
3a	C	C	A	G	A	T	C	C	G	C	T	C	C	G	T	C	T	G	G
4a	C	C	A	C	A	T	C	A	R	C	T	C	C	G	T	G	T	G	G
4d	C	C	A	C	A	T	C	A	A	C	T	C	C	G	T	G	T	G	G
4f	C	C	A	C	A	T	C	A	A	C	T	C	C	G	T	G	T	G	G

(5'- 3') (Reverse)

Subtypes	8652	8651	8650	8649	8648	8647	8646	8645	8644	8643	8642	8641	8640	8639	8638	8637	8636	8635	8634	8633	8632	8631
1a					G	G	G	R	G	C	G	G	A	G	T	A	C	C	T	G	G	T
1b			G	G	G	G	G	G	G	C	A	G	A	G	T	A	C	C	T	A	G	T
2a	C	A	G	G	A	G	G	G	G	C	A	G	A	R	T	A	C	C	T	G	G	T
2b		G	G	G	A	G	G	G	G	C	R	G	A	A	T	A	C	C	T	G	G	T
2c			G	G	R	G	G	R	G	C	G	G	A	G	T	A	C	C	T	G	G	T
2j				G	R	G	G	G	G	C	G	G	A	G	T	A	C	C	T	G	G	T
3a	C	G	G	G	T	G	G	A	G	C	A	G	A	A	T	A	C	C	T	G	G	T
4a			G	G	G	G	G	A	G	C	C	G	A	G	T	A	Y	C	T	C	G	T
4d				G	G	G	G	G	G	C	T	G	A	G	T	A	C	C	T	C	G	T
4f			G	G	G	G	G	A	G	C	C	G	A	R	T	A	C	C	T	C	G	T

RT-PCR NS5B (4)

(5'- 3') (Forward)

Subtypes	7976	7977	7978	7979	7980	7981	7982	7983	7984	7985	7986	7987	7988	7989	7990	7991	7992	7993	7994	7995	7996	7997	7998	7999	8000	8001	8002	8003
1a		C	T	T	C	T	G	G	A	A	G	A	C	A	G	T	G	T	A	A	C	A	C	C	A	A	T	A
1b	C	T	T	G	C	T	G	G	A	A	G	A	C	A	C	T	G	A	R	A	C	A	C	C	A	A	T	T
2a		C	T	C	C	T	G	G	A	A	G	A	C	T	C	A	C	A	A	A	C	A	C	C	A	A	T	T
2b		C	T	C	C	T	G	G	A	A	G	A	C	C	A	A	C	A	T	A	C	Y	C	C	A	A	T	T
2c		C	T	C	C	T	G	G	A	A	G	A	C	C	A	Y	T	G	Y	T	C	A	C	C	A	A	T	T
2j		C	T	T	T	T	G	G	A	G	G	A	C	T	C	A	R	A	C	A	C	A	C	C	A	A	T	T
3a	C	T	T	G	C	T	G	G	A	A	G	A	C	A	C	Y	A	C	A	A	C	T	C	C	A	A	T	T
4a	C	T	T	G	C	T	G	G	A	A	G	A	C	A	A	C	A	A	T	A	C	C	C	C	A	A	T	A
4d							G	G	A	A	G	A	C	A	A	C	A	C	T	A	C	C	C	C	C	A	T	C
4f	C	T	T	G	C	T	G	G	A	A	G	A	C	A	A	C	A	C	A	A	C	C	C	C	T	A	T	T

(5'- 3') (Reverse)

Subtypes	8652	8651	8650	8649	8648	8647	8646	8645	8644	8643	8642	8641	8640	8639	8638	8637	8636	8635	8634	8633	8632	8631
1a					G	G	G	R	G	C	G	G	A	G	T	A	C	C	T	G	G	T
1b			G	G	G	G	G	G	G	C	A	G	A	G	T	A	C	C	T	A	G	T
2a	C	A	G	G	A	G	G	G	G	C	A	G	A	R	T	A	C	C	T	G	G	T
2b		G	G	G	A	G	G	G	G	C	R	G	A	A	T	A	C	C	T	G	G	T
2c			G	G	R	G	G	R	G	C	G	G	A	G	T	A	C	C	T	G	G	T
2j				G	R	G	G	G	G	C	G	G	A	G	T	A	C	C	T	G	G	T
3a	C	G	G	G	T	G	G	A	G	C	A	G	A	A	T	A	C	C	T	G	G	T
4a			G	G	G	G	G	A	G	C	C	G	A	G	T	A	Y	C	T	C	G	T
4d				G	G	G	G	G	G	C	T	G	A	G	T	A	C	C	T	C	G	T
4f			G	G	G	G	G	A	G	C	C	G	A	R	T	A	C	C	T	C	G	T

RT-PCR NS5B (5)

(5'- 3') (Forward)

Subtypes	7976	7977	7978	7979	7980	7981	7982	7983	7984	7985	7986	7987	7988	7989	7990	7991	7992	7993	7994	7995	7996	7997	7998	7999	8000	8001	8002	8003
1a		C	T	T	C	T	G	G	A	A	G	A	C	A	G	T	G	T	A	A	C	A	C	C	A	A	T	A
1b	C	T	T	G	C	T	G	G	A	A	G	A	C	A	C	T	G	A	R	A	C	A	C	C	A	A	T	T
2a		C	T	C	C	T	G	G	A	A	G	A	C	T	C	A	C	A	A	A	C	A	C	C	A	A	T	T
2b		C	T	C	C	T	G	G	A	A	G	A	C	C	A	A	C	A	T	A	C	Y	C	C	A	A	T	T
2c		C	T	C	C	T	G	G	A	A	G	A	C	C	A	Y	T	G	Y	T	C	A	C	C	A	A	T	T
2j		C	T	T	T	T	G	G	A	G	G	A	C	T	C	A	R	A	C	A	C	A	C	C	A	A	T	T
3a	C	T	T	G	C	T	G	G	A	A	G	A	C	A	C	Y	A	C	A	A	C	T	C	C	A	A	T	T
4a	C	T	T	G	C	T	G	G	A	A	G	A	C	A	A	C	A	A	T	A	C	C	C	C	A	A	T	A
4d							G	G	A	A	G	A	C	A	A	C	A	C	T	A	C	C	C	C	C	A	T	C
4f	C	T	T	G	C	T	G	G	A	A	G	A	C	A	A	C	A	C	A	A	C	C	C	C	T	A	T	T

(5'- 3') (Reverse)

Subtypes	9372	9371	9370	9369	9368	9367	9366	9365	9364	9363	9362	9361	9360	9359	9358	9357	9356	9355	9354	9353	9352	9351	9350
1a	G	G	T	T	G	G	G	G	A	G	G	A	G	G	T	A	G	A	T	G	C	C	T
1b	G	G	T	T	G	G	G	G	A	G	C	A	G	G	T	A	G	A	T	G	C	C	T
2a	G	A	G	C	G	G	G	G	A	G	T	A	G	R	A	A	G	A	G	G	C	C	T
2b	G	A	G	C	G	G	G	G	A	G	T	A	A	A	A	A	G	A	T	G	C	C	T
2c	G	A	G	C	G	G	G	G	A	G	T	A	R	G	A	A	G	A	T	G	C	C	T
2j	G	A	G	C	G	G	G	G	A	G	T	A	A	A	A	A	G	A	T	G	C	C	T
3a	G	W	G	C	T	G	G	C	A	G	G	A	G	A	T	A	G	A	T	G	C	C	T
4a	G	A	G	C	A	G	G	C	A	G	C	A	G	G	T	A	G	A	T	G	C	C	T
4d	G	A	G	C	R	G	G	C	A	G	C	A	G	G	T	A	G	A	T	G	C	C	T
4f	G	A	G	C	A	G	G	C	A	G	C	A	R	R	T	A	G	A	T	G	C	C	T

Figure S3. Subtype-specific oligonucleotides designed to sequence the NS5B-coding region. Residue numbering is according to the reference strain AF009606. Positions in red are conserved among the different subtypes, and positions with different colors are discriminatory of a specific subtype (color codes given in the left column at each panel). Discriminatory positions for genotype are highlighted in pink.

PCR NS5B (3.1)

Figure S3 *(continued)*

(5'- 3') (Forward)

Subtypes	7952	7953	7954	7955	7956	7957	7958	7959	7960	7961	7962	7963	7964	7965	7966	7967	7968	7969	7970
1a	C	C	A	C	A	T	C	A	A	C	T	C	C	G	T	G	T	G	G
1b	C	C	A	C	A	T	C	C	R	C	T	C	C	G	T	G	T	G	G
2a	C	C	A	C	A	T	C	A	A	G	T	C	C	G	T	G	T	G	G
2b	C	C	A	C	A	T	C	C	G	G	T	C	C	G	T	G	T	G	G
2c	C	C	A	C	A	T	C	A	A	G	T	C	C	G	T	G	T	G	G
2j	C	C	A	C	A	T	C	M	A	G	T	C	C	G	T	G	T	G	G
3a	C	C	A	G	A	T	C	C	G	C	T	C	C	G	T	C	T	G	G
4a	C	C	A	C	A	T	C	A	R	C	T	C	C	G	T	G	T	G	G
4d	C	C	A	C	A	T	C	A	A	C	T	C	C	G	T	G	T	G	G
4f	C	C	A	C	A	T	C	A	A	C	T	C	C	G	T	G	T	G	G

(5'- 3') (Reverse)

Subtypes	8389	8388	8387	8386	8385	8384	8383	8382	8381	8380	8379	8378	8377	8376	8375	8374	8373	8372	8371	8370	8369	8368	8367
1a	C	C	R	A	C	A	T	A	A	A	G	C	C	T	C	T	C	R	G	T	G	A	G
1b	C	C	G	A	T	R	T	A	A	A	G	C	C	G	C	T	C	T	G	T	G	A	G
2a	C	C	Y	A	C	G	T	A	A	A	G	T	C	T	C	T	C	A	G	T	C	A	G
2b	C	C	T	A	C	G	T	A	R	A	G	T	C	T	C	T	C	A	G	T	G	A	G
2c	C	C	Y	A	C	G	T	A	H	A	G	T	C	T	C	T	C	A	G	T	C	A	G
2j	C	C	C	A	C	G	T	A	R	A	G	T	C	T	C	T	C	A	G	T	G	A	G
3a	C	C	G	C	A	G	T	A	A	A	G	C	C	G	C	T	C	C	G	T	G	A	G
4a	C	C	C	A	C	R	T	A	G	A	G	T	C	T	Y	T	C	T	G	T	G	A	G
4d	C	C	C	A	C	G	T	A	G	A	G	T	C	T	C	T	C	T	G	T	G	A	G
4f	C	C	C	A	C	R	T	A	G	A	G	T	C	T	Y	T	C	A	G	T	G	A	G

PCR NS5B (3.2)

(5'- 3') (Forward)

Subtypes	8142	8143	8144	8145	8146	8147	8148	8149	8150	8151	8152	8153	8154	8155	8156	8157	8158	8159	8160	8161	8162	8163	8164
1a	A	A	G	C	T	C	C	C	Y	C	T	G	G	C	C	G	T	G	A	T	G	G	G
1b	A	C	C	C	T	T	C	C	T	C	A	G	G	C	C	G	T	G	A	T	G	G	G
2a	A	A	G	C	T	T	C	C	T	C	A	G	G	C	G	G	T	G	A	T	G	G	G
2b	A	A	G	C	T	T	C	C	C	A	A	R	G	C	A	R	T	A	A	T	G	G	G
2c	A	A	G	C	T	T	C	C	C	G	T	C	G	C	R	G	T	G	A	T	G	G	G
2j	A	A	A	C	T	Y	C	C	C	C	A	G	G	C	R	G	T	G	A	T	G	G	G
3a	A	A	G	T	T	G	T	C	A	A	T	T	G	M	G	A	C	G	A	T	G	G	G
4a	M	A	A	C	T	A	C	C	T	R	A	G	G	C	C	G	T	G	A	T	G	G	G
4d	C	A	A	C	T	T	T	C	T	C	T	G	G	C	C	G	T	G	A	T	G	G	G
4f	A	A	M	C	T	C	Y	C	T	G	A	G	G	C	C	G	T	G	A	T	G	G	G

(5'- 3') (antisense)

Subtypes	8584	8583	8582	8581	8580	8579	8578	8577	8576	8575	8574	8573	8572	8571	8570	8569	8568	8567	8566	8565	8564	8563	8562
1a	C	C	C	K	S	A	C	T	T	T	C	A	C	A	G	A	T	A	A	C	G	A	C
1b	C	C	C	G	C	G	C	T	Y	T	C	A	C	A	G	A	T	A	A	C	G	A	C
2a	C	C	C	T	G	G	C	T	T	T	C	T	G	A	G	A	T	G	A	C	W	A	C
2b	C	C	T	T	G	G	C	T	C	T	C	T	G	A	G	A	T	G	A	C	R	A	C
2c	C	C	Y	T	G	A	C	T	Y	T	C	T	G	A	G	A	T	G	A	C	G	A	C
2j	C	C	C	T	G	A	C	T	C	T	C	T	G	A	G	A	T	G	A	C	R	A	C
3a	C	C	A	T	C	A	C	T	C	T	C	R	G	C	C	A	C	C	A	C	R	A	C
4a	C	C	G	T	C	G	C	T	C	T	C	A	G	C	G	A	T	R	A	C	G	A	C
4d	C	C	A	T	C	G	C	T	T	T	C	A	G	C	G	A	T	A	A	C	G	A	C
4f	C	C	A	T	C	G	C	T	T	T	C	G	G	Y	G	A	T	A	A	C	G	A	C

PCR NS5B (4.1)

(5'- 3') (Forward)

Subtypes	8018	8019	8020	8021	8022	8023	8024	8025	8026	8027	8028	8029	8030	8031	8032	8033	8034	8035	8036	8037	8038	8039	8040	8041	8042	8043	8044	8045	8046	8047	8048	8049	8050	8051	8052	8053
1a														G	T	T	T	T	C	T	G	C	G	T	T	C	A	G	C	C	T	G	A	G	A	A
1b											G	A	G	G	T	T	T	T	C	T	G	Y	G	T	C	C	A	A	C	C	A	G	A	G	A	
2a											G	A	G	G	T	G	T	T	C	T	G	C	G	T	G	G	A	C	C	C						
2b	G	G	C	C	A	A	A	A	A	T	G	A	G	G	T	G	T	T	C	T	G	T	R	T	T	G	A	T								
2c														G	T	G	T	T	C	T	G	C	G	T	Y	G	A	Y	C	C	C	R	C	C	A	
2j														G	T	G	T	T	C	T	G	C	G	T	R	G	A	Y	C	C	C	G	C	C	A	
3a		G	C	G	A	A	G	A	A	C	G	A	G	G	T	G	T	T	T	T	G	Y	G	T	G	G	A									
4a														G	T	Y	T	T	C	G	C	T	G	T	R	A	A	C	C	C	A	G	C	G	A	
4d													G	G	T	C	T	T	C	T	C	C	G	T	A	A	A	A	C	C	G	G	A	G	A	
4f				C	A	A	A	A	A	T	G	A	G	G	T	T	T	T	Y	T	C	T	G	T	Y	A	A	C	C	C	C	C	A			

(5'- 3') (Reverse)

Subtypes	8589	8588	8587	8586	8585	8584	8583	8582	8581	8580	8579	8578	8577	8576	8575	8574	8573	8572	8571	8570	8569	8568	8567	8566
1a	G	G	A	C	Y	C	C	C	K	S	A	C	T	T	T	C	A	C	A	G	A	T	A	
1b	G	G	G	T	T	C	C	C	G	C	G	C	T	Y	T	C	A	C	A	G	A	T	A	
2a	C	A	G	T	C	C	C	C	T	G	G	C	T	T	T	C	T	G	A	G	A	T	G	
2b	C	G	T	T	R	C	C	T	T	G	G	C	T	C	T	C	T	G	A	G	A	T	G	
2c				C	C	C	C	Y	T	G	A	C	T	Y	T	C	T	G	A	G	A	T	G	A
2j	C	A	G	C	Y	C	C	C	T	G	A	C	T	C	T	C	T	G	A	G	A	T	G	
3a				C	G	C	C	A	T	C	A	C	T	C	T	C	R	G	C	C	A	C	C	A
4a				C	G	C	C	G	T	C	G	C	T	C	T	C	A	G	C	G	A	T		
4d	C	C	A	C	G	C	C	A	T	C	G	C	T	T	T	C	A	G	C	G	A	T	A	
4f	C	G	A	C	A	C	C	A	T	C	G	C	T	T	T	C	G	G	Y	G	A	T	A	

PCR NS5B (5.1)

(5'- 3') (Forward)

Subtypes	8494	8495	8496	8497	8498	8499	8500	8501	8502	8503	8504	8505	8506	8507	8508	8509	8510	8511	8512	8513	8514
1a	A	G	G	C	C	C	R	R	G	C	A	G	C	C	T	G	T	C	G	A	G
1b	A	G	G	C	C	W	C	T	G	C	R	G	C	C	T	G	T	C	G	A	G
2a	A	A	G	C	C	C	T	A	G	C	G	G	C	Y	T	G	Y	A	A	G	G
2b	A	A	G	C	C	C	T	T	G	C	R	G	C	R	T	G	Y	A	A	R	G
2c	A	R	G	C	C	M	R	R	G	C	G	G	C	B	T	G	Y	A	A	M	G
2j	A	G	G	C	C	Y	T	A	G	C	R	G	C	C	T	G	T	A	A	G	G
3a	A	G	G	C	C	A	C	A	G	C	G	G	C	Y	G	C	R	A	R	G	G
4a	A	A	G	C	C	A	C	R	G	C	Y	G	C	Y	A	T	Y	A	R	R	G
4d	A	G	G	C	C	A	G	C	G	C	A	G	C	C	A	T	C	A	G	G	G
4f	A	G	G	C	Y	A	C	R	G	C	G	G	C	Y	G	C	Y	A	A	A	G

Figure S3 *(continued)*

(5'- 3') (Reverse)

Subtypes	8878	8877	8876	8875	8874	8873	8872	8871	8870	8869	8868	8867	8866	8865	8864	8863	8862	8861	8860	8859
1a	A	T	C	A	G	T	A	T	C	A	T	Y	C	T	C	G	C	C	C	A
1b	A	T	C	A	G	R	A	T	C	A	T	C	C	T	T	G	C	C	C	A
2a	A	T	Y	A	G	G	A	C	C	A	T	G	C	G	A	R	C	C	C	A
2b	A	T	T	A	T	G	A	C	C	A	T	C	C	G	G	A	C	C	C	A
2c	A	T	Y	A	G	G	A	C	C	A	T	R	C	G	C	R	C	C	C	A
2j	A	T	T	A	A	G	A	C	C	A	T	A	C	G	G	G	C	C	C	A
3a	A	T	C	A	T	Y	A	C	C	A	T	G	C	G	C	A	C	C	C	A
4a	A	T	C	A	R	T	A	Y	C	A	T	R	C	G	C	A	C	C	C	A
4d	A	T	C	A	A	A	A	C	C	A	T	G	C	G	C	A	C	C	C	A
4f	A	T	C	A	A	T	A	T	C	A	T	G	C	G	C	A	C	C	C	A

PCR NS5B (5.2)

(5'- 3') (Forward)

Subtypes	8787	8788	8789	8790	8791	8792	8793	8794	8795	8796	8797	8798	8799	8800	8801	8802	8803	8804	8805	8806	8807	8808	8809	8810
1a	G	C	G	T	G	G	G	A	G	A	C	A	G	C	A	A	G	A	C	A	C	A	C	T
1b	G	C	G	T	G	G	G	A	G	A	C	A	G	C	T	A	G	A	C	A	C	A	C	T
2a	G	C	C	T	G	G	G	A	A	A	C	A	G	T	T	A	G	A	C	A	C	T	C	C
2b	G	C	T	T	G	G	G	A	A	A	C	A	G	T	A	A	G	A	C	A	C	T	C	C
2c	G	C	C	T	G	G	G	A	G	A	C	A	G	C	A	A	G	A	C	A	C	T	C	C
2j	G	C	C	T	G	G	G	A	A	A	C	T	G	Y	A	A	A	R	C	A	C	T	C	Y
3a	G	C	T	T	G	G	G	A	R	A	C	A	G	C	T	C	G	T	C	A	C	A	C	T
4a	G	C	R	T	G	G	G	A	G	A	C	A	G	T	C	C	G	A	C	A	C	A	C	T
4d	G	C	C	T	G	G	G	A	G	A	C	A	G	C	T	C	G	A	C	A	C	A	C	T
4f	G	C	G	T	G	G	G	A	G	A	C	A	G	C	C	A	G	A	C	A	C	A	C	T

(5'- 3') (Reverse)

Subtypes	9190	9189	9188	9187	9186	9185	9184	9183	9182	9181	9180	9179	9178	9177	9176	9175	9174	9173	9172	9171
1a	A	C	T	G	C	C	C	A	R	T	T	G	A	A	G	A	G	G	T	A
1b	A	C	T	G	C	C	C	A	G	T	T	G	A	A	G	A	G	G	T	A
2a	A	C	C	G	C	C	C	A	R	T	T	G	A	A	G	A	G	A	T	A
2b	A	C	C	G	C	C	C	A	G	T	T	G	A	A	G	A	G	G	T	A
2c	A	C	A	G	C	C	C	A	G	T	T	G	A	A	G	A	G	G	T	A
2j	A	C	T	G	C	C	C	A	G	T	T	R	A	A	G	A	G	G	T	A
3a	A	C	C	G	C	C	C	A	A	T	T	R	A	A	G	A	G	R	T	A
4a	A	C	C	G	C	C	C	A	G	T	T	A	A	A	G	A	G	G	T	A
4d	A	C	C	G	C	C	C	A	G	T	T	A	A	A	G	A	G	G	T	A
4f	R	Y	C	G	C	C	C	A	G	T	T	R	A	A	G	A	G	G	T	A

PCR NS5B (5.3)

(5'- 3') (Forward)

Subtypes	9032	9033	9034	9035	9036	9037	9038	9039	9040	9041	9042	9043	9044	9045	9046	9047	9048	9049	9050	9051	9052	9053	9054	9055
1a	A	C	T	C	T	C	C	A	G	G	T	G	A	A	A	T	C	A	A	T	A	G	G	G
1b	A	C	T	C	T	C	C	A	G	G	T	G	A	R	A	T	C	A	A	T	A	G	G	G
2a	A	C	Y	C	T	C	M	C	C	A	C	G	A	A	C	T	G	A	C	G	C	G	G	G
2b	A	C	T	C	T	C	C	C	C	A	C	G	A	A	C	T	C	T	C	R	C	G	G	G
2c	A	C	T	C	T	C	M	C	C	A	C	G	A	A	C	T	C	A	S	T	C	G	G	G
2j	A	C	T	C	T	C	C	Y	C	A	T	G	A	A	C	T	C	A	C	A	C	G	G	G
3a	A	C	T	C	T	C	C	A	G	T	A	G	A	G	C	T	C	A	A	Y	A	G	G	G
4a	A	C	T	C	T	C	C	A	C	A	C	G	A	A	C	T	C	A	A	Y	C	G	G	G
4d	A	C	T	C	T	C	C	A	C	A	C	G	A	A	C	T	C	A	A	C	C	G	G	G
4f	A	C	T	C	T	C	C	A	C	A	C	G	A	A	C	T	C	A	A	T	C	G	G	G

(5'- 3') (Reverse)

Subtypes	9348	9347	9346	9345	9344	9343	9342	9341	9340	9339	9338	9337	9336	9335	9334	9333	9332	9331	9330	9329	9328
1a	C	C	C	C	T	G	C	A	G	C	A	A	G	C	A	G	G	A	G	T	A
1b	C	C	C	C	T	A	C	R	G	A	A	A	G	T	A	G	G	A	G	T	A
2a	C	C	C	C	T	A	C	R	R	A	A	A	G	T	A	G	G	A	G	T	A
2b	C	Y	C	C	T	A	C	G	C	T	A	A	G	T	A	G	G	A	G	T	A
2c	C	C	C	C	T	A	C	R	C	A	A	A	G	t	A	G	G	A	G	T	A
2j	C	C	C	C	T	A	C	A	A	G	A	A	G	T	A	G	G	A	G	T	A
3a	C	C	C	C	T	A	C	C	G	T	T	A	G	T	A	G	G	A	G	T	A
4a	C	C	C	C	T	A	C	K	G	W	a	A	G	T	A	G	G	A	G	T	A
4d	C	C	C	C	T	A	C	G	G	A	A	A	G	T	A	G	G	A	G	T	A
4f	C	C	C	C	T	A	C	T	G	A	A	A	G	T	A	G	G	A	G	T	A

PCR NS5B (5.4)

(5'- 3') (Forward)

Subtypes	8018	8019	8020	8021	8022	8023	8024	8025	8026	8027	8028	8029	8030	8031	8032	8033	8034	8035
1a														G	T	T	T	T
1b											G	A	G	G	T	T	T	T
2a											G	A	G	G	T	G	T	T
2b	G	G	C	C	A	A	A	A	A	T	G	A	G	G	T	G	T	T
2c														G	T	G	T	T
2j														G	T	G	T	T
3a		G	C	G	A	A	G	A	A	C	G	A	G	G	T	G	T	T
4a														G	T	Y	T	T
4d													G	G	T	C	T	T
4f				C	A	A	A	A	A	T	G	A	G	G	T	T	T	T

Subtypes	8036	8037	8038	8039	8040	8041	8042	8043	8044	8045	8046	8047	8048	8049	8050	8051	8052	8053
1a	C	T	G	C	G	T	T	C	A	G	C	C	T	G	A	G	A	A
1b	C	T	G	Y	G	T	C	C	A	A	C	C	A	G	A	G	A	
2a	C	T	G	C	G	T	G	G	A	C	C	C						
2b	C	T	G	T	R	T	T	G	A	T								
2c	C	T	G	C	G	T	Y	G	A	Y	C	C	C	R	C	C	A	
2j	C	T	G	C	G	T	R	G	A	Y	C	C	C	G	C	C	A	
3a	T	T	G	Y	G	T	G	G	A									
4a	C	G	C	T	G	T	R	A	A	C	C	C	A	G	C	G	A	
4d	C	T	C	C	G	T	A	A	A	A	C	C	G	G	A	G	A	
4f	Y	T	C	T	G	T	Y	A	A	C	C	C	C	C	A			

(5'- 3') (Reverse)

Subtypes	9348	9347	9346	9345	9344	9343	9342	9341	9340	9339	9338	9337	9336	9335	9334	9333	9332	9331	9330	9329	9328
1a	C	C	C	C	T	G	C	A	G	C	A	A	G	C	A	G	G	A	G	T	A
1b	C	C	C	C	T	A	C	R	G	A	A	A	G	T	A	G	G	A	G	T	A
2a	C	C	C	C	T	A	C	R	R	A	A	A	G	T	A	G	G	A	G	T	A
2b	C	Y	C	C	T	A	C	G	C	T	A	A	G	T	A	G	G	A	G	T	A
2c	C	C	C	C	T	A	C	R	C	A	A	A	G	t	A	G	G	A	G	T	A
2j	C	C	C	C	T	A	C	A	A	G	A	A	G	T	A	G	G	A	G	T	A
3a	C	C	C	C	T	A	C	C	G	T	T	A	G	T	A	G	G	A	G	T	A
4a	C	C	C	C	T	A	C	K	G	W	a	A	G	T	A	G	G	A	G	T	A
4d	C	C	C	C	T	A	C	G	G	A	A	A	G	T	A	G	G	A	G	T	A
4f	C	C	C	C	T	A	C	T	G	A	A	A	G	T	A	G	G	A	G	T	A
