# Supplementary material for: Pipeline for specific subtype amplification and drug resistance detection in hepatitis C virus
Source: BMC Infect Dis. 2018 Sep 3;18:446. doi: 10.1186/s12879-018-3356-6 (PMC6122477; doi:10.1186/s12879-018-3356-6)
Supplement: Supplementary file 7 — Table S4. Subtype-specific oligonucleotides designed to sequence the NS3-coding region. (PDF 157 kb) [file 12879_2018_3356_MOESM7_ESM.pdf]

Table S4. Subtype-specific oligonucleotides designed to sequence the NS3-coding region.

Subtype	PCR Name	Sense	Primer Name	Sequence (5'-3')[a]	Position[b]	Tm (ºC)
1a	**External oligonucleotides (5'-3')**					
	**RT-PCR 1**	**Fw**	**NS3u1a3391**	GAATGGTCTCCAAGGGGTGGAGGT	3391-3414	60.8
		**Rv**	**NS3d1a4004**	CTGGGGCACTGCTGGTGGRGA	4004-3984	60.2-62.2
	**Internal oligonucleotides (5'-3')**					
	**PCR 1.1**	**Fw**	**13u1a3490**	GTTGTAAAACGACGGCCAGTGGGACAAAAACCARGTGGAGGGT	3490-3512	57.1-58.8
	**PCR 1.1**	**Rv**	**13d1a3982**	CACAGGAAACAGCTATGACCGAGTTGTCYGTGAACACCGGGGACC	3982-3958	62.6-64.2
	**PCR 1.2**	**Fw**	**NS3M13u1a3438**	GTTGTAAAACGACGGCCAGTGCCCAGCAGACRAGGGGCCT	3438-3457	60-62
	**PCR 1.2**	**Rv**	**NS3M13d1a3982**	CACAGGAAACAGCTATGACCGAGTTGTCYGTGAACACCGGGGA	3982-3960	58.8-60.6
1b	**External oligonucleotides (5'-3')**					
	**RT-PCR 1**	**Fw**	**NS3u1b3394**	TTGARGGGCAGGGGTGGCGRCT	3394-3415	60.4-64.2
		**Rv**	**NS3d1b4007**	TGTCTGCGGTACRGCCGGRGG	4007-3987	60.2-64.1
	**Internal oligonucleotides (5'-3')**					
	**PCR 1.1**	**Fw**	**13u1b3490**	GTTGTAAAACGACGGCCAGTGGGACAATAACCAGGTCGAGGGG	3490-3512	60.6
	**PCR 1.1**	**Rv**	**13d1b3982**	CACAGGAAACAGCTATGACCGARTTGTCYGTGAAGACCGGRGACC	3982-3958	59.3-64.2
	**PCR 1.2**	**Fw**	**NS3M13u1b3439**	GTTGTAAAACGACGGCCAGTCCCAACAGACGCGGGGCCTACTT	3439-3461	62.4
	**PCR 1.2**	**Rv**	**NS3M13d1b3982**	CACAGGAAACAGCTATGACCGARTTGTCYGTGAAGACCGGRGACC	3982-3958	59.3-64.2
2a	**External oligonucleotides (5'-3')**					
	**RT-PCR 1**	**Fw**	**NS3u2a3387**	GATGGCTACACCTCYAAGGGGTGGA	3387-3411	61-62.6
		**Rv**	**NS3d2a4007**	GGTCTGGGGCACAGCYGGTGG	4007-3987	62.2-64.1
	**Internal oligonucleotides (5'-3')**					
	**PCR 1.1**	**Fw**	**13u2a3490**	GTTGTAAAACGACGGCCAGTGYGACARGACAGAACAGGCCGGG	3490-3512	60.6-64.2
	**PCR 1.1**	**Rv**	**13d2a3982**	CACAGGAAACAGCTATGACCCTGTTGTCACTRAAGGTGGGAGACC	3982-3958	59.3-61
	**PCR 1.2**	**Fw**	**NS3M13u2a3438**	GTTGTAAAACGACGGCCAGTGCCCAGCARACACGAGGYCT	3438-3457	55.9-60
	**PCR 1.2**	**Rv**	**NS3M13d2a3983**	CACAGGAAACAGCTATGACCGCTGTTGTCACTRAAGGTGGGRGA	3983-3960	57.4-60.8
2b	**External oligonucleotides (5'-3')**					
	**RT-PCR 1**	**Fw**	**NS3u2b3391**	GCTACACCTCGAAGGGGTGGAAGCT	3391-3415	62.6
		**Rv**	**NS3d2b4004**	CTGGGGCACAGCTGGTGGYGT	4004-3984	60.2-62.2
	**Internal oligonucleotides (5'-3')**					
	**PCR 1.1**	**Fw**	**13u2b3490**	GTTGTAAAACGACGGCCAGTGCGACAAAAATGAGCAGGCCGGG	3490-3512	60.6
	**PCR 1.1**	**Rv**	**13d2b3982**	CACAGGAAACAGCTATGACCCTGTTGTCAGAGAAACTGGGCGWCC	3982-3958	61
	**PCR 1.2**	**Fw**	**NS3M13u2b3438**	GTTGTAAAACGACGGCCAGTACYCAGCAGACTCGTGGYCTCCT	3438-3460	58.8-62.4
	**PCR 1.2**	**Rv**	**NS3M13d2b3985**	CACAGGAAACAGCTATGACCGTRCTGTTGTCAGAGAAACTGGGCG	3985-3961	59.3-61
2c	**External oligonucleotides (5'-3')**					
	**RT-PCR 1**	**Fw**	**NS3u2c3384**	GCTGATGGATAYACCTCYAARGGTTGGA	3384-3411	58.5-62.9
		**Rv**	**NS3d2c4007**	GGTCTGGGGCACAGCTGGAGG	4007-3987	62.2
	**Internal oligonucleotides (5'-3')**					
	**PCR 1.1**	**Fw**	**13u2c3490**	GTTGTAAAACGACGGCCAGTGYGACAAGACRGAMCAGGCTGGG	3490-3512	58.8-64.2
	**PCR 1.1**	**Rv**	**13d2c3982**	CACAGGAAACAGCTATGACCCTGTTGTCAGTRAAGYTTGGAGACC	3982-3958	56-59.3
	**PCR 1.2**	**Fw**	**NS3M13u2c3438**	GTTGTAAAACGACGGCCAGTGCCCAGCAAACCCGAGGKCT	3438-3457	57.9-60

	**PCR 1.2**	**Rv**	**NS3M13d2c3982**	CACAGGAAACAGCTATGACCCTGTTGTCAGTRAARYTTGGAGACCT	3982-3957	54.8-59.5
**2j**	**External oligonucleotides (5'-3')**					
	**RT-PCR 1**	**Fw**	**NS3u2j3387**	GACGAGTACGTCTCCARGGGRTGGA	3387-3411	61-64.2
		**Rv**	**NS3d2j4005**	TCTGGGGCACAGCTGGRGGCGT	4005-3984	62.3-64.2
	**Internal oligonucleotides (5'-3')**					
	**PCR 1.1**	**Fw**	**13u2j3490**	GTTGTAAAACGACGGCCAGTGCGAYAAAACRGAGTGTGCCGGA	3490-3512	57.1-60.6
	**PCR 1.1**	**Rv**	**13d2j3982**	CACAGGAAACAGCTATGACCCTGTTGTCAGTGAARCTGGGGGACC	3982-3958	61-62.6
	**PCR 1.2**	**Fw**	**NS3M13u2j3438**	GTTGTAAAACGACGGCCAGTGCTCAGCAGACCCGTGGCCT	3438-3457	60
	**PCR 1.2**	**Rv**	**NS3M13d2j3983**	CACAGGAAACAGCTATGACCGCTGTTGTCAGTGAARCTGGGGGA	3983-3960	59.1-60.8
**3a**	**External oligonucleotides (5'-3')**					
	**RT-PCR 1**	**Fw**	**NS3u3a3387**	GATGAYTATCGGGAGATGGGTTGGC	3387-3411	59.3-61
		**Rv**	**NS3d3a4004**	CTGTGGRACRGCAGGAGGAGTTGAAT	4004-3979	59.5-62.7
	**Internal oligonucleotides (5'-3')**					
	**PCR 1.1**	**Fw**	**13u3a3490**	GTTGTAAAACGACGGCCAGTGGGAYAAGAAYGTGGTGACCGGT	3490-3512	57.1-60.6
	**PCR 1.1**	**Rv**	**13d3a3982**	CACAGGAAACAGCTATGACCGAATTGTCAGAGAARGATGGAGACC	3982-3958	56-57.7
	**PCR 1.2**	**Fw**	**NS3M13u3a3438**	GTTGTAAAACGACGGCCAGTGCCCAGCAAACYAGRGGCCTT	3438-3458	56.3-60.2
	**PCR 1.2**	**Rv**	**NS3M13d3a3988**	CACAGGAAACAGCTATGACCGGAGTTGAATTGTCAGAGAARGATGGAGA	3988-3960	58.7-60.1
**4a**	**External oligonucleotides (5'-3')**					
	**RT-PCR 1**	**Fw**	**NS3u4a3391**	CAGAAACATCMAAGGGGTGGAGACT	3391-3415	57.7-59.3
		**Rv**	**NS3d4a4004**	CTGRGGCACYGCDGGGGGTGT	4004-3984	60.2-66.1
	**Internal oligonucleotides (5'-3')**					
	**PCR 1.1**	**Fw**	**13u4a3490**	GTTGTAAAACGACGGCCAGTGGGACACCAATGARAAATTGTGGT	3490-3512	53.5-55.3
	**PCR 1.1**	**Rv**	**13d4a3982**	CACAGGAAACAGCTATGACCGARTTGTCAGTGAACACTGGTGATC	3982-3958	56-57.7
	**PCR 1.2**	**Fw**	**NS3M13u4a3439**	GTTGTAAAACGACGGCCAGTCGCAGCAGACCCGCGGCTT	3439-3457	59.7
	**PCR 1.2**	**Rv**	**NS3M13d4a3985**	CACAGGAAACAGCTATGACCGTTGARTTGTCAGTGAACACTGGTGA	3985-3960	56.4-58
**4d**	**External oligonucleotides (5'-3')**					
	**RT-PCR 1**	**Fw**	**NS3u4d3391**	CGCACRCYATRAAGGGGTGGA	3391-3411	54.4-60.2
		**Rv**	**NS3d4d4004**	CTGGGGCACGGCAGGAGGAGT	4004-3984	62.2
	**Internal oligonucleotides (5'-3')**					
	**PCR 1.1**	**Fw**	**13u4d3490**	GTTGTAAAACGACGGCCAGTGAGACACCAACGAGAACTGCGGT	3490-3512	58.8
	**PCR 1.1**	**Rv**	**13d4d3982**	CACAGGAAACAGCTATGACCGAATTGTCAGAGAATACTGGRGACC	3982-3958	56-57.7
	**PCR 1.2**	**Fw**	**NS3M13u4d3439**	GTTGTAAAACGACGGCCAGTCGCAGCAGACCCGAGGGAT	3439-3457	57.6
	**PCR 1.2**	**Rv**	**NS3M13d4d3985**	CACAGGAAACAGCTATGACCGTGGAATTGTCAGAGAATACTGGAGA	3985-3960	56.4
**4f**	**External oligonucleotides (5'-3')**					
	**RT-PCR 1**	**Fw**	**NS3u4f3392**	TGAGGTGTCRGGGGGGTGGA	3392-3411	57.9-60
		**Rv**	**NS3d4f4004**	CTGGGGCACAGCAGGAGGCGT	4004-3984	62.2
	**Internal oligonucleotides (5'-3')**					
	**PCR 1.1**	**Fw**	**13u4f3490**	GTTGTAAAACGACGGCCAGTGGGAYACTAAYGAGAAYTGTGGT	3490-3512	51.7-57.1
	**PCR 1.1**	**Rv**	**13d4f3982**	CACAGGAAACAGCTATGACCGARTTGTCAGARAAGACCGGGGATC	3982-3958	57.7-61
	**PCR 1.2**	**Fw**	**NS3M13u4f3438**	GTTGTAAAACGACGGCCAGTGCGCAGCAAACTCGAGGCTT	3438-3457	55.9
	**PCR 1.2**	**Rv**	**NS3M13d4f3982**	CACAGGAAACAGCTATGACCGARTTGTCAGAAAAGACCGGGGA	3982-3960	55.3-57.1

[a]The underlined nucleotides indicate universal M13 oligonucleotide

[b]Residue numbering according to the reference strain AF009606
