# Supplementary material for: Pipeline for specific subtype amplification and drug resistance detection in hepatitis C virus
Source: BMC Infect Dis. 2018 Sep 3;18:446. doi: 10.1186/s12879-018-3356-6 (PMC6122477; doi:10.1186/s12879-018-3356-6)
Supplement: Supplementary file 8 — Table S5. Subtype-specific oligonucleotides designed to sequence the NS5A-coding region. (PDF 158 kb) [file 12879_2018_3356_MOESM8_ESM.pdf]

Table S5. Subtype-specific oligonucleotides designed to sequence the NS5A-coding region.

Subtype	PCR Name	Sense	Primer Name	Sequence (5’-3’)[a]	Position[b]	Tm (ºC)
**1a**	**External oligonucleotides (5’-3’)**					
	**RT-PCR 2**	**Fw**	**NS5Au1a6234**	AGCTCGGAGTGYACCACTCCRT	6234-6255	56.7-60.4
		**Rv**	**NS5Ad1a6879**	GGGAGGGATCAGTGAGCATGGA	6879-6858	58.6
	**Internal oligonucleotides (5’-3’)**					
	**PCR 2.1**	**Fw**	**13u1a6299**	GTTGTAAAACGACGGCCAGTGGTGCTGAGCGAYTTTAAGACCTGGCT	6299-6325	61.3-62.8
	**PCR 2.1**	**Rv**	**13d1a6735**	CACAGGAAACAGCTATGACCTATGYAGGCGCACCCCGTCCA	6735-6715	58.3-60.2
	**PCR 2.2**	**Fw**	**NS5AM13u1a6260**	GTTGTAAAACGACGGCCAGTCGGTTCCTGGCTAAGGGACAT	6260-6280	56.3
	**PCR 2.2**	**Rv**	**NS5AM13d1a6799**	CACAGGAAACAGCTATGACCTCGTGKAGTCCYACTCTGAAYGA	6799-6777	53.5-58.8
**1b**	**External oligonucleotides (5’-3’)**					
	**RT-PCR 2**	**Fw**	**NS5Au1b6234**	AAYGAGGACTGCTCCACGCCAT	6234-6255	56.7-58.6
		**Rv**	**NS5Ad1b6879**	GGGAGGGGTCGGTGAGCAT	6879-6861	57.6
	**Internal oligonucleotides (5’-3’)**					
	**PCR 2.1**	**Fw**	**13u1b6299**	GTTGTAAAACGACGGCCAGTGGTGTTGACTGAYTTCAAGACCTGGCT	6299-6325	59.7-61.3
	**PCR 2.1**	**Rv**	**13d1b6735**	CACAGGAAACAGCTATGACCTGTGCARCCGYACCCCRTCCA	6735-6715	56.3-62.2
	**PCR 2.2**	**Fw**	**NS5AM13u1b6259**	GTTGTAAAACGACGGCCAGTCCGGCTCGTGGCTAAGGGA	6259-6277	57.6
	**PCR 2.2**	**Rv**	**NS5AM13d1b6799**	CACAGGAAACAGCTATGACCTGGTTGAGCCCGACCTGGAATGT	6799-6777	58.8
**2a**	**External oligonucleotides (5’-3’)**					
	**RT-PCR 2**	**Fw**	**NS5Au2a6234**	ACTGAGGACTGCCCCATCCCAT	6234-6255	58.6
		**Rv**	**NS5Ad2a6880**	TGGGAYGGATCTGTTAGCATGGA	6880-6858	55.3-57.1
	**Internal oligonucleotides (5’-3’)**					
	**PCR 2.1**	**Fw**	**13u2a6299**	GTTGTAAAACGACGGCCAGTCATCCTRACAGACTTYAAAAAYTGGCT	6299-6325	52.1-58.2
	**PCR 2.1**	**Rv**	**13d2a6735**	CACAGGAAACAGCTATGACCTRTGGATYTGCACTCCGTCCA	6735-6715	52.4-56.3
	**PCR 2.2**	**Fw**	**NS5AM13u2a6260**	GTTGTAAAACGACGGCCAGTCGGMTCVTGGCTCCGCGAYGT	6260-6280	58.3-64.1
	**PCR 2.2**	**Rv**	**NS5AM13d2a6793**	CACAGGAAACAGCTATGACCAGCCCAACGCWRAACGAGACCTC	6793-6771	58.8-60.6
**2b**	**External oligonucleotides (5’-3’)**					
	**RT-PCR 2**	**Fw**	**NS5Au2b6230**	GATCACTGAAGATTGCCCAGTCCCAT	6230-6255	59.5
		**Rv**	**NS5Ad2b6879**	GGGACGGGTCTGTCAACATGGA	6879-6858	58.6
	**Internal oligonucleotides (5’-3’)**					
	**PCR 2.1**	**Fw**	**13u2b6299**	GTTGTAAAACGACGGCCAGTCATYCTCACAGACTTTAAGAACTGGCT	6299-6325	56.7-58.2
	**PCR 2.1**	**Rv**	**13d2b6735**	CACAGGAAACAGCTATGACCGGTGTATYTGCACCCCGTCCA	6735-6715	56.3-58.3
	**PCR 2.2**	**Fw**	**NS5AM13u2b6260**	GTTGTAAAACGACGGCCAGTGGGGTCTTGGCTCCRGGA	6260-6277	54.9-57.2
	**PCR 2.2**	**Rv**	**NS5AM13d2b6799**	CACAGGAAACAGCTATGACCGAATTGAGGCCYACRGTGAACGT	6799-6777	55.3-58.8
**2c**	**External oligonucleotides (5’-3’)**					
	**RT-PCR 2**	**Fw**	**NS5Au2c6237**	GARGAYTGCCCYGTCCCCT	6237-6255	53.2-59.7
		**Rv**	**NS5Ad2c6882**	TGTGGGAYGGGTCYGTTAGCAT	6882-6861	54.8-58.6
	**Internal oligonucleotides (5’-3’)**					
	**PCR 2.1**	**Fw**	**13u2c6299**	GTTGTAAAACGACGGCCAGTRATCCTGACAGACTTYAAGARTTGGCT	6299-6325	55.2-59.7
	**PCR 2.1**	**Rv**	**13d2c6735**	CACAGGAAACAGCTATGACCGATGGATYTGRACYCCGTCCA	6735-6715	52.4-58.3
	**PCR 2.2**	**Fw**	**NS5AM13u2c6256**	GTTGTAAAACGACGGCCAGTGCTCYRGCTCATGGCTYCGAGA	6256-6277	56.7-62.3

	**PCR 2.2**	**Rv**	**NS5AM13d2c6799**	CACAGGAAACAGCTATGACCGAGTTGAGMCCCACGCWGAACGA	6799-6777	58.8-60.6
**2j**	**External oligonucleotides (5'-3')**					
	**RT-PCR 2**	**Fw**	**NS5Au2j6229**	GGATCACTGAGGACTGYCCCGT	6229-6250	58.6-60.4
		**Rv**	**NS5Ad2j6878**	GGAYGGGTCCGTCAGCATGGA	6878-6858	58.3-60.2
	**Internal oligonucleotides (5'-3')**					
	**PCR 2.1**	**Fw**	**13u2j6299**	GTTGTAAAACGACGGCCAGTCATCTTGACTGACTTYAARAAYTGGCT	6299-6325	53.7-58.2
	**PCR 2.1**	**Rv**	**13d2j6735**	CACAGGAAACAGCTATGACCGRTGGATCTGYACCCCRTCCA	6735-6715	54.4-60.2
	**PCR 2.2**	**Fw**	**NS5AM13u2j6261**	GTTGTAAAACGACGGCCAGTGGCTCTTGGCTTCGCGACATAT	6261-6282	56.7
	**PCR 2.2**	**Rv**	**NS5AM13d2j6799**	CACAGGAAACAGCTATGACCGAGTTGAGGCCCACGCTAAACGA	6799-6777	58.8
**3a**	**External oligonucleotides (5'-3')**					
	**RT-PCR 2**	**Fw**	**NS5Au3a6230**	GATCAATGAAGACTACCCAAGYCCTT	6230-6255	56.4-58
		**Rv**	**NS5Ad3a6874**	GGGTCTCTCAACATCGAGGTCA	6874-6853	56.7
	**Internal oligonucleotides (5'-3')**					
	**PCR 2.1**	**Fw**	**13u3a6299**	GTTGTAAAACGACGGCCAGTGGTGYTGTCYGACTTCAAGACATGGCT	6299-6325	59.7-62.8
	**PCR 2.1**	**Rv**	**13d3a6735**	CACAGGAAACAGCTATGACCGGTGGAGTCTCACCCCRTCCA	6735-6715	58.3-60.2
	**PCR 2.2**	**Fw**	**NS5AM13u3a6260**	GTTGTAAAACGACGGCCAGTCGGYGATTGGCTGCGTACCAT	6260-6280	56.3-58.3
	**PCR 2.2**	**Rv**	**NS5AM13d3a6799**	CACAGGAAACAGCTATGACCGAATTCAWCCCTACCRTGAAAGTGAT	6799-6774	54.8-56.4
**4a**	**External oligonucleotides (5'-3')**					
	**RT-PCR 2**	**Fw**	**NS5Au4a6230**	GATCAATGAAGATTGYTCCACYCCAT	6230-6255	54.8-58
		**Rv**	**NS5Ad4a6879**	GTGATGGGTCTGTCARCATGGA	6879-6858	54.8-56.7
	**Internal oligonucleotides (5'-3')**					
	**PCR 2.1**	**Fw**	**13u4a6299**	GTTGTAAAACGACGGCCAGTCGTGCTGAGTGACTTCAAGACGTGGCT	6299-6325	62.8
	**PCR 2.1**	**Rv**	**13d4a6735**	CACAGGAAACAGCTATGACCGGTGTAGYCTGAYGCCGTCYA	6735-6715	54.4-60.2
	**PCR 2.2**	**Fw**	**NS5AM13u4a6260**	GTTGTAAAACGACGGCCAGTCGAATCTTGGCTRTGGGAGGT	6260-6280	54.4-56.3
	**PCR 2.2**	**Rv**	**NS5AM13d4a6799**	CACAGGAAACAGCTATGACCGAATTGAGTCCTACYGAGAACGA	6799-6777	53.5-55.3
**4d**	**External oligonucleotides (5'-3')**					
	**RT-PCR 2**	**Fw**	**NS5Au4d6234**	AACGAGGACTGCTCTACTCCTT	6234-6255	54.8
		**Rv**	**NS5Ad4d6879**	GAGATGGATCTGTCAGCATAGA	6879-6858	53
	**Internal oligonucleotides (5'-3')**					
	**PCR 2.1**	**Fw**	**13u4d6299**	GTTGTAAAACGACGGCCAGTCGTACTGAGTGACTTTAAAACGTGGCT	6299-6325	58.2
	**PCR 2.1**	**Rv**	**13d4d6735**	CACAGGAAACAGCTATGACCGATGGAGCCTGACGCCATCCA	6735-6715	58.3
	**PCR 2.2**	**Fw**	**NS5AM13u4d6256**	GTTGTAAAACGACGGCCAGTGTGATCGCTCTTGGTTATGGGAGAT	6256-6280	57.7
	**PCR 2.2**	**Rv**	**NS5AM13d4d6799**	CACAGGAAACAGCTATGACCGTGTTGAGGCCCACTGTAAAGGA	6799-6777	57.1
**4f**	**External oligonucleotides (5'-3')**					
	**RT-PCR 2**	**Fw**	**NS5Au4f6227**	GTGGATCAATGAAGACTGYCCYACT	6227-6251	56-59.3
		**Rv**	**NS5Ad4f6879**	GGGATGGGTCTGTAAGCATGGA	6879-6858	56.7
	**Internal oligonucleotides (5'-3')**					
	**PCR 2.1**	**Fw**	**13u4f6299**	GTTGTAAAACGACGGCCAGTCGTAYTGTCTGACTTYAAGACYTGGCT	6299-6325	56.7-61.3
	**PCR 2.1**	**Rv**	**13d4f6735**	CACAGGAAACAGCTATGACCGGTGAAKTCTRACRCCATCCA	6735-6715	50.5-56.3
	**PCR 2.2**	**Fw**	**NS5AM13u4f6256**	GTTGTAAAACGACGGCCAGTGTGACTCATCYTGGCTRTGGGA	6256-6277	54.8-58.6
	**PCR 2.2**	**Rv**	**NS5AM13d4f6798**	CACAGGAAACAGCTATGACCAGTTRAGGCCCACGGAGAACGT	6798-6777	56.7-58.6

[a]The underlined nucleotides indicate universal M13 oligonucleotide

[b]Residue numbering according to the reference strain AF009606
