# Supplementary material for: Pipeline for specific subtype amplification and drug resistance detection in hepatitis C virus
Source: BMC Infect Dis. 2018 Sep 3;18:446. doi: 10.1186/s12879-018-3356-6 (PMC6122477; doi:10.1186/s12879-018-3356-6)
Supplement: Supplementary file 9 — Table S6. Subtype-specific oligonucleotides designed to sequence the NS5B-coding region. (PDF 250 kb) [file 12879_2018_3356_MOESM9_ESM.pdf]

Table S6. Subtype-specific oligonucleotides designed to sequence the NS5B-coding region.

Subtype	PCR Name	Sense	Primer Name	Sequence (5’-3’)[a]	Position[b]	Tm (ºC)
1a	**External oligonucleotides (5’-3’)**					
	**RT-PCR 3**	**Fw**	**5Bu1a7952**	CCACATCAACTCCGTGTGG	7952-7970	53.2
	**RT-PCR 3/4**	**Rv**	**NS5Bd1a8648**	GGGRGCGGAGTACCTGGT	8648-8631	54.9-57.2
	**RT-PCR 5/4**	**Fw**	**NS5Bu1a7977**	CTTCTGGAAGACAGTGTAACACCAATA	7977-8003	56.7
	**RT-PCR 5**	**Rv**	**NS5Bd1a9372**	GGTTGGGGAGGAGGTAGATGCCT	9372-9350	60.6
	**Internal oligonucleotides (5’-3’)**					
	**PCR 3.1**	**Fw**	**13u1a7952**	GTTGTAAAACGACGGCCAGTCCACATCAACTCCGTGTGG	7952-7970	53.2
	**PCR 3.1**	**Rv**	**13d1a8389**	CACAGGAAACAGCTATGACCCCRACATAAAGCCTCTCRGTGAG	8389-8367	55.3-58.8
	**PCR 3.2**	**Fw**	**13u1a8142**	GTTGTAAAACGACGGCCAGTAAGCTCCCYCTGGCCGTGATGGG	8142-8164	62.4-64.2
	**PCR 3.2**	**Rv**	**13d1a8584**	CACAGGAAACAGCTATGACCCCCKSACTTTCACAGATAACGAC	8584-8562	55.3-57.1
	**PCR 5.1**	**Fw**	**13u1a8494**	GTTGTAAAACGACGGCCAGTAGGCCCRRGCAGCCTGTCGAG	8494-8514	60.2-64.1
	**PCR 5.1**	**Rv**	**13d1a8878**	CACAGGAAACAGCTATGACCATCAGTATCATYCTCGCCCA	8878-8859	49.7-51.8
	**PCR 5.2**	**Fw**	**13u1a8787**	GTTGTAAAACGACGGCCAGTGCGTGGGAGACAGCAAGACACACT	8787-8810	60.8
	**PCR 5.2**	**Rv**	**13d1a9190**	CACAGGAAACAGCTATGACCACTGCCCARTTGAAGAGGTA	9190-9171	49.7-51.8
	**PCR 5.3**	**Fw**	**13u1a9032**	GTTGTAAAACGACGGCCAGTACTCTCCAGGTGAAATCAATAGGG	9032-9055	55.7
	**PCR 5.3/5.4**	**Rv**	**NS5BM13d1a9348**	CACAGGAAACAGCTATGACCCCCCTGCAGCAAGCAGGAGTA	9348-9328	58.3
	**PCR 4.1/5.4**	**Fw**	**NS5BM13u1a8031**	GTTGTAAAACGACGGCCAGTGTTTTCTGCGTTCAGCCTGAGAA	8031-8053	55.3
	**PCR 4.1**	**Rv**	**NS5BM13d1a8589**	CACAGGAAACAGCTATGACCGGACYCCCKSACTTTCACAGATA	8589-8567	55.3-58.8
1b	**External oligonucleotides (5’-3’)**					
	**RT-PCR 3**	**Fw**	**5Bu1b7952**	CCACATCCRCTCCGTGTGG	7952-7970	55.4-57.6
	**RT-PCR 3/4**	**Rv**	**NS5Bd1b8650**	GGGGGGGCAGAGTACCTAGT	8650-8631	57.9
	**RT-PCR 5/4**	**Fw**	**NS5Bu1b7976**	CTTGCTGGAAGACACTGARACACCAATT	7976-8003	58.5-59.9
	**RT-PCR 5**	**Rv**	**NS5Bd1b9372**	GGTTGGGGAGCAGGTAGATGCCT	9372-9350	60.6
	**Internal oligonucleotides (5’-3’)**					
	**PCR 3.1**	**Fw**	**13u1b7952**	GTTGTAAAACGACGGCCAGTCCACATCCRCTCCGTGTGG	7952-7970	55.4-57.6
	**PCR 3.1**	**Rv**	**13d1b8389**	CACAGGAAACAGCTATGACCCCGATRTAAAGCCGCTCTGTGAG	8389-8367	57.1-58.8
	**PCR 3.2**	**Fw**	**13u1b8142**	GTTGTAAAACGACGGCCAGTACCCTTCCTCAGGCCGTGATGGG	8142-8164	62.4
	**PCR 3.2**	**Rv**	**13d1b8584**	CACAGGAAACAGCTATGACCCCCGCGCTYTCACAGATAACGAC	8584-8562	58.8-60.6
	**PCR 5.1**	**Fw**	**13u1b8494**	GTTGTAAAACGACGGCCAGTAGGCCWCTGCRGCCTGTCGAG	8494-8514	60.2-62.2
	**PCR 5.1**	**Rv**	**13d1b8878**	CACAGGAAACAGCTATGACCATCAGRATCATCCTTGCCCA	8878-8859	49.7-51.8
	**PCR 5.2**	**Fw**	**13u1b8787**	GTTGTAAAACGACGGCCAGTGCGTGGGAGACAGCTAGACACACT	8787-8810	60.8
	**PCR 5.2**	**Rv**	**13d1b9190**	CACAGGAAACAGCTATGACCACTGCCCAGTTGAAGAGGTA	9190-9171	51.8
	**PCR 5.3**	**Fw**	**13u1b9032**	GTTGTAAAACGACGGCCAGTACTCTCCAGGTGARATCAATAGGG	9032-9055	55.7-57.4
	**PCR 5.3/5.4**	**Rv**	**NS5BM13d1b9348**	CACAGGAAACAGCTATGACCCCCCTACRGAAAGTAGGAGTA	9348-9328	52.4-54.4
	**PCR 4.1/5.4**	**Fw**	**NS5BM13u1b8028**	GTTGTAAAACGACGGCCAGTGAGGTTTTCTGYGTCCAACCAGAGA	8028-8052	57.7-59.3
	**PCR 4.1**	**Rv**	**NS5BM13d1b8589**	CACAGGAAACAGCTATGACCGGGTTCCCGC	8589-8567	

				GCTYTCACAGATA		58.8-60.6
**2a**	**External oligonucleotides (5’-3’)**					
	**RT-PCR 3**	**Fw**	**5Bu2a7952**	CCACATCAAGTCCGTGTGG	7952-7970	53.2
	**RT-PCR 3/4**	**Rv**	**NS5Bd2a8652**	CAGGAGGGGCAGARTACCTGGT	8652-8631	58.6-60.4
	**RT-PCR 5/4**	**Fw**	**NS5Bu2a7977**	CTCCTGGAAGACTCACAAACACCAATT	7977-8003	58.2
	**RT-PCR 5**	**Rv**	**NS5Bd2a9372**	GAGCGGGGAGTAGRAAGAGGCCT	9372-9350	60.6-62.4
	**Internal oligonucleotides (5’-3’)**					
	**PCR 3.1**	**Fw**	**13u2a7952**	GTTGTAAAACGACGGCCAGTACCACATCAAGTCCGTGTGG	7952-7970	53.2
	**PCR 3.1**	**Rv**	**13d2a8389**	CACAGGAAACAGCTATGACCCCYACGTAAAGTCTCTCAGTCAG	8389-8367	55.3-57.1
	**PCR 3.2**	**Fw**	**13u2a8142**	GTTGTAAAACGACGGCCAGTAAGCTTCCTCAGGCGGTGATGGG	8142-8164	60.6
	**PCR 3.2**	**Rv**	**13d2a8584**	CACAGGAAACAGCTATGACCCCCTGGCTTTCTGAGATGACWAC	8584-8562	57.1
	**PCR 5.1**	**Fw**	**13u2a8494**	GTTGTAAAACGACGGCCAGTAAGCCCTAGCGGCYTGYAAGG	8494-8514	56.3-60.2
	**PCR 5.1**	**Rv**	**13d2a8878**	CACAGGAAACAGCTATGACCATYAGGACCATGCGARCCCA	8878-8859	51.8-5.9
	**PCR 5.2**	**Fw**	**13u2a8787**	GTTGTAAAACGACGGCCAGTGCCTGGGAAACAGTTAGACACTCC	8787-8810	59.1
	**PCR 5.2**	**Rv**	**13d2a9190**	CACAGGAAACAGCTATGACCACCGCCCARTTGAAGAGATA	9190-9171	49.7-51.8
	**PCR 5.3**	**Fw**	**13u2a9032**	GTTGTAAAACGACGGCCAGTACYCTCMCCACGAACTGACGCGGG	9032-9055	62.5-65.9
	**PCR 5.3/5.4**	**Rv**	**NS5BM13d2a9348**	CACAGGAAACAGCTATGACCCCCCTACRRAAAGTAGGAGTA	9348-9328	50.5-54.4
	**PCR 4.1/5.4**	**Fw**	**NS5BM13u2a8028**	GTTGTAAAACGACGGCCAGTGAGGTGTTCTGCGTGGACCC	8028-8047	57.9
	**PCR 4.1**	**Rv**	**NS5BM13d2a8589**	CACAGGAAACAGCTATGACCCAGTCCCCTGGCTTTCTGAGATG	8589-8567	58.8
**2b**	**External oligonucleotides (5’-3’)**					
	**RT-PCR 3**	**Fw**	**5Bu2b7952**	CCACATCCGGTCCGTGTGG	7952-7970	57.6
	**RT-PCR 3/4**	**Rv**	**NS5Bd2b8651**	GGGAGGGGCRGAATACCTGGT	8651-8631	58.3-60.2
	**RT-PCR 5/4**	**Fw**	**NS5Bu2b7977**	CTCCTGGAAGACCAACATACYCCAATT	7977-8003	58.2-59.7
	**RT-PCR 5**	**Rv**	**NS5Bd2b9372**	GAGCGGGGAGTAAAAAGATGCCT	9372-9350	57.1
	**Internal oligonucleotides (5’-3’)**					
	**PCR 3.1**	**Fw**	**13u2b7952**	GTTGTAAAACGACGGCCAGTCCACATCCGGTCCGTGTGG	7952-7970	57.6
	**PCR 3.1**	**Rv**	**13d2b8389**	CACAGGAAACAGCTATGACCCCTACGTARAGTCTCTCAGTGAG	8389-8367	55.3-57.1
	**PCR 3.2**	**Fw**	**13u2b8142**	GTTGTAAAACGACGGCCAGTAAGCTTCCCAARGCARTAATGGG	8142-8164	53.5-57.1
	**PCR 3.2**	**Rv**	**13d2b8584**	CACAGGAAACAGCTATGACCCCTTGGCTCTCTGAGATGACRAC	8584-8562	57.1-58.8
	**PCR 5.1**	**Fw**	**13u2b8494**	GTTGTAAAACGACGGCCAGTAAGCCCTTGCAGCRTGYAARG	8494-8514	52.4-58.3
	**PCR 5.1**	**Rv**	**13d2b8878**	CACAGGAAACAGCTATGACCATTATGACCATCCGGACCCA	8878-8859	51.8
	**PCR 5.2**	**Fw**	**13u2b8787**	GTTGTAAAACGACGGCCAGTGCTTGGGAAACAGTAAGACACTCC	8787-8810	57.4
	**PCR 5.2**	**Rv**	**13d2b9190**	CACAGGAAACAGCTATGACCACCGCCCAGTTGAAGAGGTA	9190-9171	53.8
	**PCR 5.3**	**Fw**	**13u2b9032**	GTTGTAAAACGACGGCCAGTACTCTCCCCACGAACTCTCRCGGG	9032-9055	62.5-64.2
	**PCR 5.3/5.4**	**Rv**	**NS5BM13d2b9348**	CACAGGAAACAGCTATGACCCYCCTACGCTAAGTAGGAGTA	9348-9328	52.4-54.4
	**PCR 4.1/5.4**	**Fw**	**NS5BM13u2b8018**	GTTGTAAAACGACGGCCAGTGGCCAAAAATGAGGTGTTCTGTGTTGAT	8018-8045	58.5
	**PCR 4.1**	**Rv**	**NS5BM13d2b8589**	CACAGGAAACAGCTATGACCCGTTRCCTTGGCTCTCTGAGATG	8589-8567	57.1-58.8

2c	**External oligonucleotides (5’-3’)**					
	**RT-PCR 3**	**Fw**	**5Bu2c7952**	CCACATCAAGTCCGTGTGG	7952-7970	53.2
	**RT-PCR 3/4**	**Rv**	**NS5Bd2c8650**	GGRGGRGCGGAGTACCTGGT	8650-8631	57.9-62
	**RT-PCR 5/4**	**Fw**	**NS5Bu2c7977**	CTCCTGGAAGACCAYTGYTCACCAATT	7977-8003	58.2-61.3
	**RT-PCR 5**	**Rv**	**NS5Bd2c9372**	GAGCGGGGAGTARGAAGATGCCT	9372-9350	58.8-60.6
	**Internal oligonucleotides (5’-3’)**					
	**PCR 3.1**	**Fw**	**13u2c7952**	GTTGTAAAACGACGGCCAGTCCACATCAAGTCCGTGTGG	7952-7970	53.2
	**PCR 3.1**	**Rv**	**13d2c8389**	CACAGGAAACAGCTATGACCCCYACGTAHAGTCTCTCAGTCAG	8389-8367	55.3-58.8
	**PCR 3.2**	**Fw**	**13u2c8142**	GTTGTAAAACGACGGCCAGTAAGCTTCCCGTCGCRGTGATGGG	8142-8164	60.6-62.4
	**PCR 3.2**	**Rv**	**13d2c8584**	CACAGGAAACAGCTATGACCCCYTGACTYTCTGAGATGACGAC	8584-8562	55.3-58.8
	**PCR 5.1**	**Fw**	**13u2c8494**	GTTGTAAAACGACGGCCAGTARGCCAGGGCGGCBTGYAACG	8494-8514	58.3-64.1
	**PCR 5.1**	**Rv**	**13d2c8878**	CACAGGAAACAGCTATGACCATYAGGACCATRCGCRCCCA	8878-8859	51.8-57.9
	**PCR 5.2**	**Fw**	**13u2c8787**	GTTGTAAAACGACGGCCAGTGCCTGGGAGACAGCAAGACACTCC	8787-8810	62.5
	**PCR 5.2**	**Rv**	**13d2c9190**	CACAGGAAACAGCTATGACCACAGCCCAGTTGAAGAGGTA	9190-9171	51.8
	**PCR 5.3**	**Fw**	**13u2c9032**	GTTGTAAAACGACGGCCAGTACTCTCMCCACGAACTCASTCGGG	9032-9055	60.8-62.5
	**PCR 5.3/5.4**	**Rv**	**NS5BM13d2c9348**	CACAGGAAACAGCTATGACCCCCCTACRCAAAGTAGGAGTA	9348-9328	52.4-54.4
	**PCR 4.1/5.4**	**Fw**	**NS5BM13u2c8031**	GTTGTAAAACGACGGCCAGTGTGTTCTGCGTYGAYCCCRCCA	8031-8052	56.7-62.3
	**PCR 4.1**	**Rv**	**NS5BM13d2c8586**	CACAGGAAACAGCTATGACCCCCCYTGACTYTCTGAGATGA	8586-8566	52.4-56.3
2j	**External oligonucleotides (5’-3’)**					
	**RT-PCR 3**	**Fw**	**5Bu2j7952**	CCACATCMAGTCCGTGTGG	7952-7970	53.2-55.4
	**RT-PCR 3/4**	**Rv**	**NS5Bd2j8649**	GRGGGGCGGAGTACCTGGT	8649-8631	57.6-59.7
	**RT-PCR 5/4**	**Fw**	**NS5Bu2j7977**	CTTTTGGAGGACTCARACACACCAATT	7977-8003	56.7-58.2
	**RT-PCR 5**	**Rv**	**NS5Bd2j9372**	GAGCGGGGAGTAAAAAGATGCCT	9372-9350	57.1
	**Internal oligonucleotides (5’-3’)**					
	**PCR 3.1**	**Fw**	**13u2j7952**	GTTGTAAAACGACGGCCAGTCCACATCMAGTCCGTGTGG	7952-7970	53.2-55.4
	**PCR 3.1**	**Rv**	**13d2j8389**	CACAGGAAACAGCTATGACCCCCACGTARAGTCTCTCAGTGAG	8389-8367	57.1-58.8
	**PCR 3.2**	**Fw**	**13u2j8142**	GTTGTAAAACGACGGCCAGTAAACTYCCCCAGGCRGTGATGGG	8142-8164	58.8-62.4
	**PCR 3.2**	**Rv**	**13d2j8584**	CACAGGAAACAGCTATGACCCCCTGACTCTCTGAGATGACRAC	8584-8562	57.1-58.8
	**PCR 5.1**	**Fw**	**13u2j8494**	GTTGTAAAACGACGGCCAGTAGGCCYTAGCRGCCTGTAAGG	8494-8514	56.3-60.2
	**PCR 5.1**	**Rv**	**13d2j8878**	CACAGGAAACAGCTATGACCATTAAGACCATACGGGCCCA	8878-8859	51.8
	**PCR 5.2**	**Fw**	**13u2j8787**	GTTGTAAAACGACGGCCAGTGCCTGGGAAACTGYAAARCACTCY	8787-8810	55.7-60.8
	**PCR 5.2**	**Rv**	**13d2j9190**	CACAGGAAACAGCTATGACCACTGCCCAGTTRAAGAGGTA	9190-9171	49.7-51.8
	**PCR 5.3**	**Fw**	**13u2j9032**	GTTGTAAAACGACGGCCAGTACTCTCCYCATGAACTCACACGGG	9032-9055	59.1-60.8
	**PCR 5.3/5.4**	**Rv**	**NS5BM13d2j9348**	CACAGGAAACAGCTATGACCCCCCTACAAGAAGTAGGAGTA	9348-9328	52.4
	**PCR 4.1/5.4**	**Fw**	**NS5BM13u2j8031**	GTTGTAAAACGACGGCCAGTGTGTTCTGCGTRGAYCCCGCCA	8031-8052	58.6-62.3
	**PCR 4.1**	**Rv**	**NS5BM13d2j8589**	CACAGGAAACAGCTATGACCCAGCYCCCTGACTCTCTGAGATG	8589-8567	58.8-60.6

**3a**	**External oligonucleotides (5’-3’)**					
	**RT-PCR 3**	**Fw**	**5Bu3a7952**	CCAGATCCGCTCCGTCTGG	7952-7970	57.6
	**RT-PCR 3/4**	**Rv**	**NS5Bd3a8652**	CGGGTGGAGCAGAATACCTGGT	8652-8631	58.6
	**RT-PCR 5/4**	**Fw**	**NS5Bu3a7976**	CTTGCTGGAAGACACYACAACTCCAATT	7976-8003	58.5-59.9
	**RT-PCR 5**	**Rv**	**NS5Bd3a9372**	GWGCTGGCAGGAGATAGATGCCT	9372-9350	58.8
	**Internal oligonucleotides (5’-3’)**					
	**PCR 3.1**	**Fw**	**13u3a7952**	GTTGTAAAACGACGGCCAGTCCAGATCCGC TCCGTCTGG	7952-7970	57.6
	**PCR 3.1**	**Rv**	**13d3a8389**	CACAGGAAACAGCTATGACCCCGCAGTAAA GCCGCTCCGTGAG	8389-8367	62.4
	**PCR 3.2**	**Fw**	**13u3a8142**	GTTGTAAAACGACGGCCAGTAAGTTGTCAA TTGMGACGATGGG	8142-8164	53.5-55.3
	**PCR 3.2**	**Rv**	**13d3a8584**	CACAGGAAACAGCTATGACCCCATCACTCT CRGCCACCACRAC	8584-8562	58.8-62.4
	**PCR 5.1**	**Fw**	**13u3a8494**	GTTGTAAAACGACGGCCAGTAGGCCACAGC GGCYGCRARGG	8494-8514	60.2-66.1
	**PCR 5.1**	**Rv**	**13d3a8878**	CACAGGAAACAGCTATGACCATCATYACCA TGCGCACCCA	8878-8859	51.8-53.8
	**PCR 5.2**	**Fw**	**13u3a8787**	GTTGTAAAACGACGGCCAGTGCTTGGGARA CAGCTCGTCACACT	8787-8810	59.1-60.8
	**PCR 5.2**	**Rv**	**13d3a9190**	CACAGGAAACAGCTATGACCACCGCCCAAT TRAAGAGRTA	9190-9171	47.7-51.8
	**PCR 5.3**	**Fw**	**13u3a9032**	GTTGTAAAACGACGGCCAGTACTCTCCAGT AGAGCTCAAYAGGG	9032-9055	57.4-59.1
	**PCR 5.3/5.4**	**Rv**	**NS5BM13d3a9348**	CACAGGAAACAGCTATGACCCCCCTACCGT TAGTAGGAGTA	9348-9328	54.4
	**PCR 4.1/5.4**	**Fw**	**NS5BM13u3a8019**	GTTGTAAAACGACGGCCAGTGCGAAGAACG AGGTGTTTTGYGTGGA	8019-8044	59.5-61.1
	**PCR 4.1**	**Rv**	**NS5BM13d3a8586**	CACAGGAAACAGCTATGACCCGCCATCACT CTCRGCGACCA	8586-8566	58.3-60.2
**4a**	**External oligonucleotides (5’-3’)**					
	**RT-PCR 3**	**Fw**	**5Bu4a7952**	CCACATCARCTCCGTGTGG	7952-7970	53.2-55.4
	**RT-PCR 3/4**	**Rv**	**NS5Bd4a8650**	GGGGGAGCCGAGTAYCTCGT	8650-8631	57.9-60
	**RT-PCR 5/4**	**Fw**	**NS5Bu4a7976**	CTTGCTGGAAGACAACAATACCCCAATA	7976-8003	58.5
	**RT-PCR 5**	**Rv**	**NS5Bd4a9372**	GAGCAGGCAGCAGGTAGATGCCT	9372-9350	60.6
	**Internal oligonucleotides (5’-3’)**					
	**PCR 3.1**	**Fw**	**13u4a7952**	GTTGTAAAACGACGGCCAGTCCACATCARC TCCGTGTGG	7952-7970	53.2-55.4
	**PCR 3.1**	**Rv**	**13d4a8389**	CACAGGAAACAGCTATGACCCCCACRTAGA GTCTYTCTGTGAG	8389-8367	55.3-58.8
	**PCR 3.2**	**Fw**	**13u4a8142**	GTTGTAAAACGACGGCCAGTMAACTACCTR AGGCCGTGATGGG	8142-8164	57.1-60.6
	**PCR 3.2**	**Rv**	**13d4a8584**	CACAGGAAACAGCTATGACCCCGTCGCTCT CAGCGATRACGAC	8584-8562	60.6-62.4
	**PCR 5.1**	**Fw**	**13u4a8494**	GTTGTAAAACGACGGCCAGTAAGCCACRGC CGCTATYARAG	8494-8514	52.4-58.3
	**PCR 5.1**	**Rv**	**13d4a8878**	CACAGGAAACAGCTATGACCATCARTAYCA TRCGCACCCA	8878-8859	47.7-53.8
	**PCR 5.2**	**Fw**	**13u4a8787**	GTTGTAAAACGACGGCCAGTGCRTGGGAGA CAGTCCGACACACT	8787-8810	60.8-62.5
	**PCR 5.2**	**Rv**	**13d4a9190**	CACAGGAAACAGCTATGACCACCGCCCAGT TAAAGAGGTA	9190-9171	51.8
	**PCR 5.3**	**Fw**	**13u4a9032**	GTTGTAAAACGACGGCCAGTACTCTCCACA CGAACTCAAYCGGG	9032-9055	59.1-60.8
	**PCR 5.3/5.4**	**Rv**	**NS5BM13d4a9348**	CACAGGAAACAGCTATGACCCCCCTACKG WAAGTAGGAGTA	9348-9328	52.4-54.4
	**PCR 4.1/5.4**	**Fw**	**NS5BM13u4a8031**	GTTGTAAAACGACGGCCAGTGTYTTCGCTG TRAACCCAGCGA	8031-8052	54.8-58.6
	**PCR 4.1**	**Rv**	**NS5BM13d4a8586**	CACAGGAAACAGCTATGACCCGCCGTCGCT CTCAGCGAT	8586-8568	57.6

**4d**	**External oligonucleotides (5’-3’)**					
	**RT-PCR 3**	**Fw**	**5Bu4d7952**	CCACATCAACTCCGTGTGG	7952-7970	53.2
	**RT-PCR 3/4**	**Rv**	**NS5Bd4d8649**	GGGGGGCTGAGTACCTCGT	8649-8631	57.6
	**RT-PCR 5/4**	**Fw**	**NS5Bu4d7982**	GGAAGACAACACTACCCCCATC	7982-8003	56.7
	**RT-PCR 5**	**Rv**	**NS5Bd4d9372**	GAGCRGGCAGCAGGTAGATGCCT	9372-9350	60.6-62.4
	**Internal oligonucleotides (5’-3’)**					
	**PCR 3.1**	**Fw**	**13u4d7952**	GTTGTAAAACGACGGCCAGTCCACATCAAC TCCGTGTGG	7952-7970	53.2
	**PCR 3.1**	**Rv**	**13d4d8389**	CACAGGAAACAGCTATGACCCCCACGTAGA GTCTCTCTGTGAG	8389-8367	58.8
	**PCR 3.2**	**Fw**	**13u4d8142**	GTTGTAAAACGACGGCCAGTCAACTTTCTC TGGCCGTGATGGG	8142-8164	58.8
	**PCR 3.2**	**Rv**	**13d4d8584**	CACAGGAAACAGCTATGACCCCATCGCTTT CAGCGATAACGAC	8584-8562	57.1
	**PCR 5.1**	**Fw**	**13u4d8494**	GTTGTAAAACGACGGCCAGTAGGCCAGCGC AGCCATCAGGG	8494-8514	62.2
	**PCR 5.1**	**Rv**	**13d4d8878**	CACAGGAAACAGCTATGACCATCAAAACCA TGCGCACCCA	8878-8859	51.8
	**PCR 5.2**	**Fw**	**13u4d8787**	GTTGTAAAACGACGGCCAGTGCCTGGGAGA CAGCTCGACACACT	8787-8810	62.5
	**PCR 5.2**	**Rv**	**13d4d9190**	CACAGGAAACAGCTATGACCACCGCCCAGT TAAAGAGGTA	9190-9171	51.8
	**PCR 5.3**	**Fw**	**13u4d9032**	GTTGTAAAACGACGGCCAGTACTCTCCACA CGAACTCAACCGGG	9032-9055	60.8
	**PCR 5.3/5.4**	**Rv**	**NS5BM13d4d9348**	CACAGGAAACAGCTATGACCCCCCTACGGA AAGTAGGAGTA	9348-9328	54.4
	**PCR 4.1/5.4**	**Fw**	**NS5BM13u4d8030**	GTTGTAAAACGACGGCCAGTGGTCTTCTCC GTAAAACCGGAGA	8030-8052	57.1
	**PCR 4.1**	**Rv**	**NS5BM13d4d8589**	CACAGGAAACAGCTATGACCCCACGCCATC GCTTTCAGCGATA	8589-8567	58.8
**4f**	**External oligonucleotides (5’-3’)**					
	**RT-PCR 3**	**Fw**	**5Bu4f7952**	CCACATCAACTCCGTGTGG	7952-7970	53.2
	**RT-PCR 3/4**	**Rv**	**NS5Bd4f8650**	GGGGGAGCCGARTACCTCGT	8650-8631	57.9-60
	**RT-PCR 5/4**	**Fw**	**NS5Bu4f7976**	CTTGCTGGAAGACAACACAACCCCTATT	7976-8003	59.9
	**RT-PCR 5**	**Rv**	**NS5Bd4f9372**	GAGCAGGCAGCARRTAGATGCCT	9372-9350	57.1-60.6
	**Internal oligonucleotides (5’-3’)**					
	**PCR 3.1**	**Fw**	**13u4f7952**	GTTGTAAAACGACGGCCAGTCCACATCAAC TCCGTGTGG	7952-7970	53.2
	**PCR 3.1**	**Rv**	**13d4f8389**	CACAGGAAACAGCTATGACCCCCACRTAGA GTCTYTCAGTGAG	8389-8367	55.3-58.8
	**PCR 3.2**	**Fw**	**13u4f8142**	GTTGTAAAACGACGGCCAGTAAMCTCYCTG AGGCCGTGATGGG	8142-8164	58.8-62.4
	**PCR 3.2**	**Rv**	**13d4f8584**	CACAGGAAACAGCTATGACCCCATCGCTTT CGGYGATAACGAC	8584-8562	57.1-58.8
	**PCR 5.1**	**Fw**	**13u4f8494**	GTTGTAAAACGACGGCCAGTAGGCYACRGC GGCCGCYAAAG	8494-8514	58.3-64.1
	**PCR 5.1**	**Rv**	**13d4f8878**	CACAGGAAACAGCTATGACCATCAATATCA TGCGCACCCA	8878-8859	49.7
	**PCR 5.2**	**Fw**	**13u4f8787**	GTTGTAAAACGACGGCCAGTGCGTGGGAGA CAGCCAGACACACT	8787-8810	62.5
	**PCR 5.2**	**Rv**	**13d4f9190**	CACAGGAAACAGCTATGACCRYCGCCCAGT TRAAGAGGTA	9190-9171	49.7-55.9
	**PCR 5.3**	**Fw**	**13u4f9032**	GTTGTAAAACGACGGCCAGTACTCTCCACA CGAACTCAATCGGG	9032-9055	59.1
	**PCR 5.3/5.4**	**Rv**	**NS5BM13d4f9348**	CACAGGAAACAGCTATGACCCCCCTACTGA AAGTAGGAGTA	9348-9328	52.4
	**PCR 4.1/5.4**	**Fw**	**NS5BM13u4f8021**	GTTGTAAAACGACGGCCAGTCAAAAATGAG GTTTTYTCTGTYAACCCCCA	8021-8050	57.5-60.3
	**PCR 4.1**	**Rv**	**NS5BM13d4f8589**	CACAGGAAACAGCTATGACCCGACACCATC GCTTTCGGYGATA	8589-8567	57.1-58.8

[a]The underlined nucleotides indicate universal M13 oligonucleotide

[b]Residue numbering according to the reference strain AF009606
