# Supplementary material for: Pipeline for specific subtype amplification and drug resistance detection in hepatitis C virus
Source: BMC Infect Dis. 2018 Sep 3;18:446. doi: 10.1186/s12879-018-3356-6 (PMC6122477; doi:10.1186/s12879-018-3356-6)
Supplement: Supplementary file 10 — Figure S4. Comparative analysis using inter- and intra- sequencing platforms. Viral RNAs of two G1b HCV-infected patients (termed 8101 and JRR) at treatment (simeprevir/sofosbuvir/ribavirin) failure were used to amplify NS3- (PCR 1.1 in Fig. 2), NS5A- (PCR 2.1 in Fig. 2), and NS5B-coding regions (PCRs 3.1 and 3.2 in Fig. 2), following the procedure described in Materials ans Methods. Amplicons derived from 8101 viral RNA amplifications were sequenced in parallel using MiSeq and 454 GSJunior platforms (inter-sequencing platforms comparison). Amplicons derived from JRR viral RNA amplifications were sequenced twice in two different runs using MiSeq platform (MiSeq A and B) (intra-sequencing platform comparison). Percentage of common and unique haplotypes and reads between both runs at each comparison are represented. Scatter plots comparing the percentage of variants obtained according to the two runs of each amplicon are shown on the right. (PDF 415 kb) [file 12879_2018_3356_MOESM10_ESM.pdf]

Figure S4

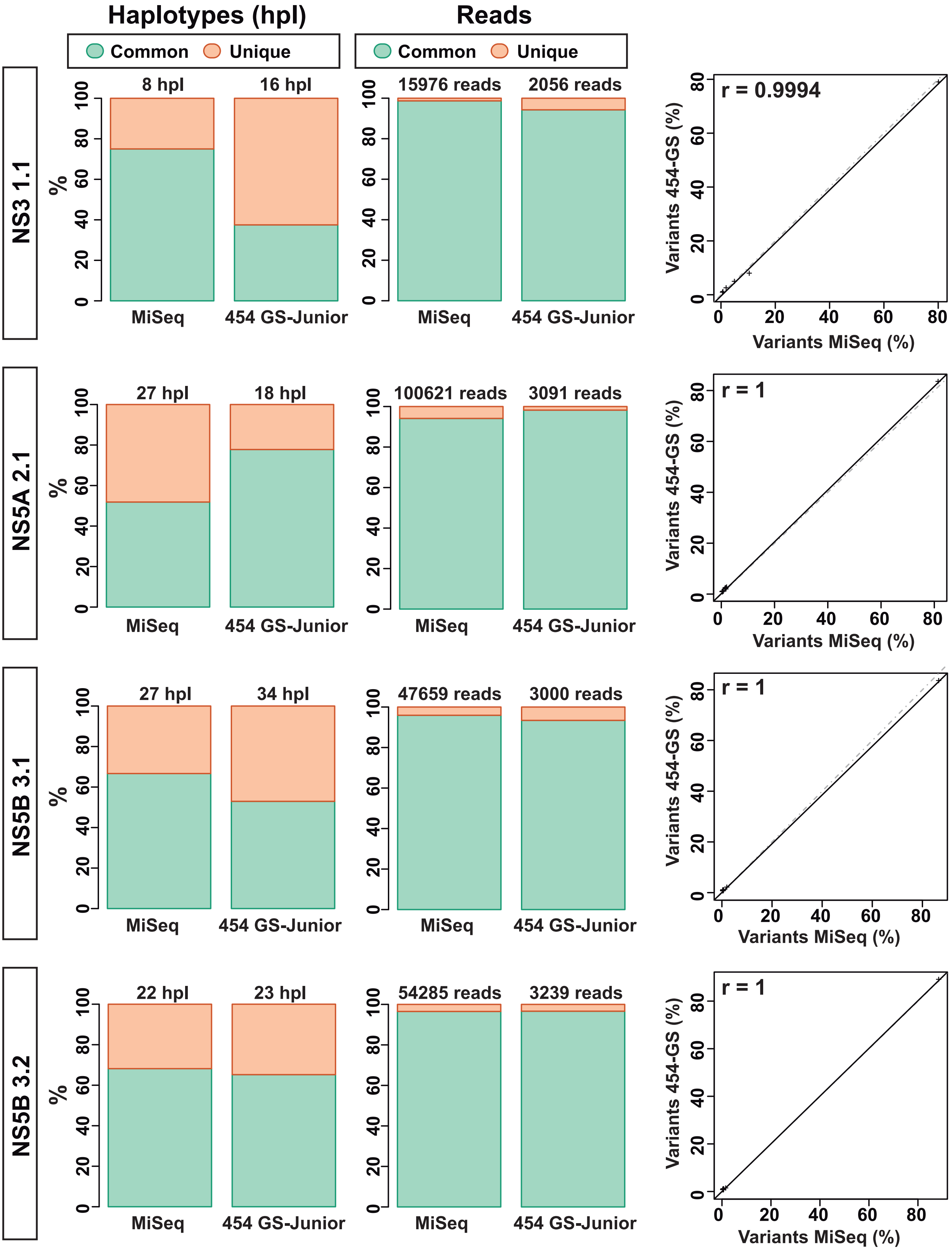

Haplotypes (hpl)
Reads
Common
Unique
NS3 1.1
NS5A 2.1
NS5B 3.1
NS5B 3.2
%
8 hpl
16 hpl
15976 reads
2056 reads
27 hpl
18 hpl
100621 reads
3091 reads
27 hpl
34 hpl
47659 reads
3000 reads
22 hpl
23 hpl
54285 reads
3239 reads
MiSeq
454 GS-Junior
r = 0.9994
r = 1
Variants 454-GS (%)
Variants MiSeq (%)

Figure S4 *(continued)*

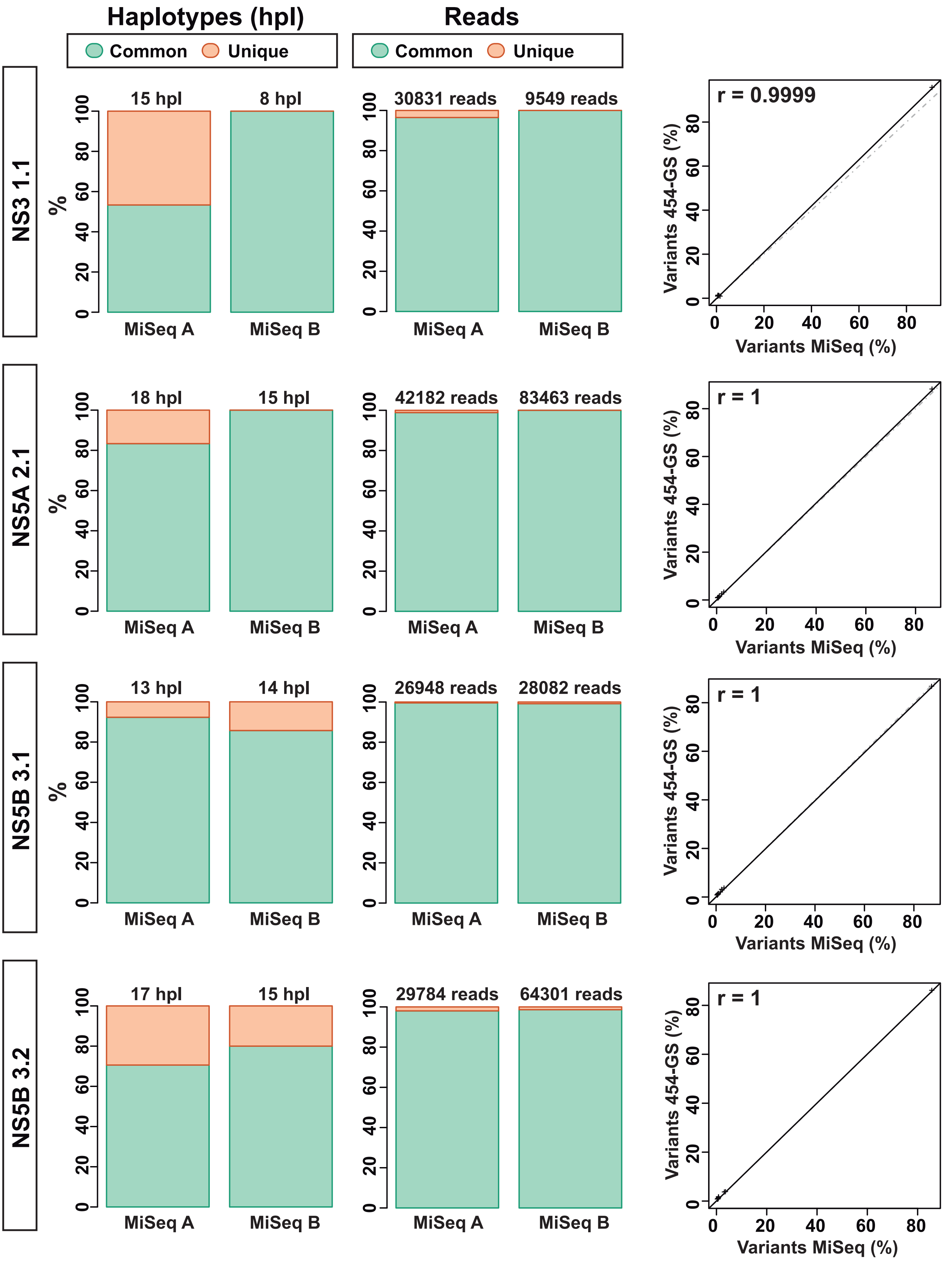

Figure S4. Comparative analysis using inter- and intra- sequencing platforms. Viral RNAs of two G1b HCV-infected patients (termed 8101 and JRR) at treatment (simeprevir/sofosbuvir/ribavirin) failure were used to amplify NS3- (PCR 1.1 in Figure 2), NS5A- (PCR 2.1 in Figure 2), and NS5B-coding regions (PCRs 3.1 and 3.2 in Figure 2), following the procedure described in Materials ans Methods. Amplicons derived from 8101 viral RNA amplifications were sequenced in parallel using MiSeq and 454 GS-Junior platforms (inter-sequencing platforms comparison). Amplicons derived from JRR viral RNA amplifications were sequenced twice in two different runs using MiSeq platform (MiSeq A and B) (intra-sequencing platform comparison). Percentage of common and unique haplotypes and reads between both runs at each comparison are represented. Scatter plots comparing the percentage of variants obtained according to the two runs of each amplicon are shown on the right.
