# Supplementary material for: Pipeline for specific subtype amplification and drug resistance detection in hepatitis C virus
Source: BMC Infect Dis. 2018 Sep 3;18:446. doi: 10.1186/s12879-018-3356-6 (PMC6122477; doi:10.1186/s12879-018-3356-6)
Supplement: Supplementary file 11 — Figure S5. Control of basal error. A full-length HCV DNA encoding NS5A with amino acid substitutions N248 K, E269K and A346V was used as a template to determine the basal error of the amplification and sequencing process using 454 GS-Junior and Illumina MiSeq platforms. Due to restriction size of amplicon length, only N248 K and E269K were detected. Experiments were performed in triplicate. Haplotypes obtained after amplification and sequencing are numbered on the left of each replicate, and percentages of reads that include the indicated substitutions are shown on the right. Artifacts means mutations other than those encoding N248 K and E269K. Basal error average is the mean ± standard deviation of the haplotypes number 2, which are those including artifacts found at the highest frequency. (PDF 291 kb) [file 12879_2018_3356_MOESM11_ESM.pdf]

Figure S5

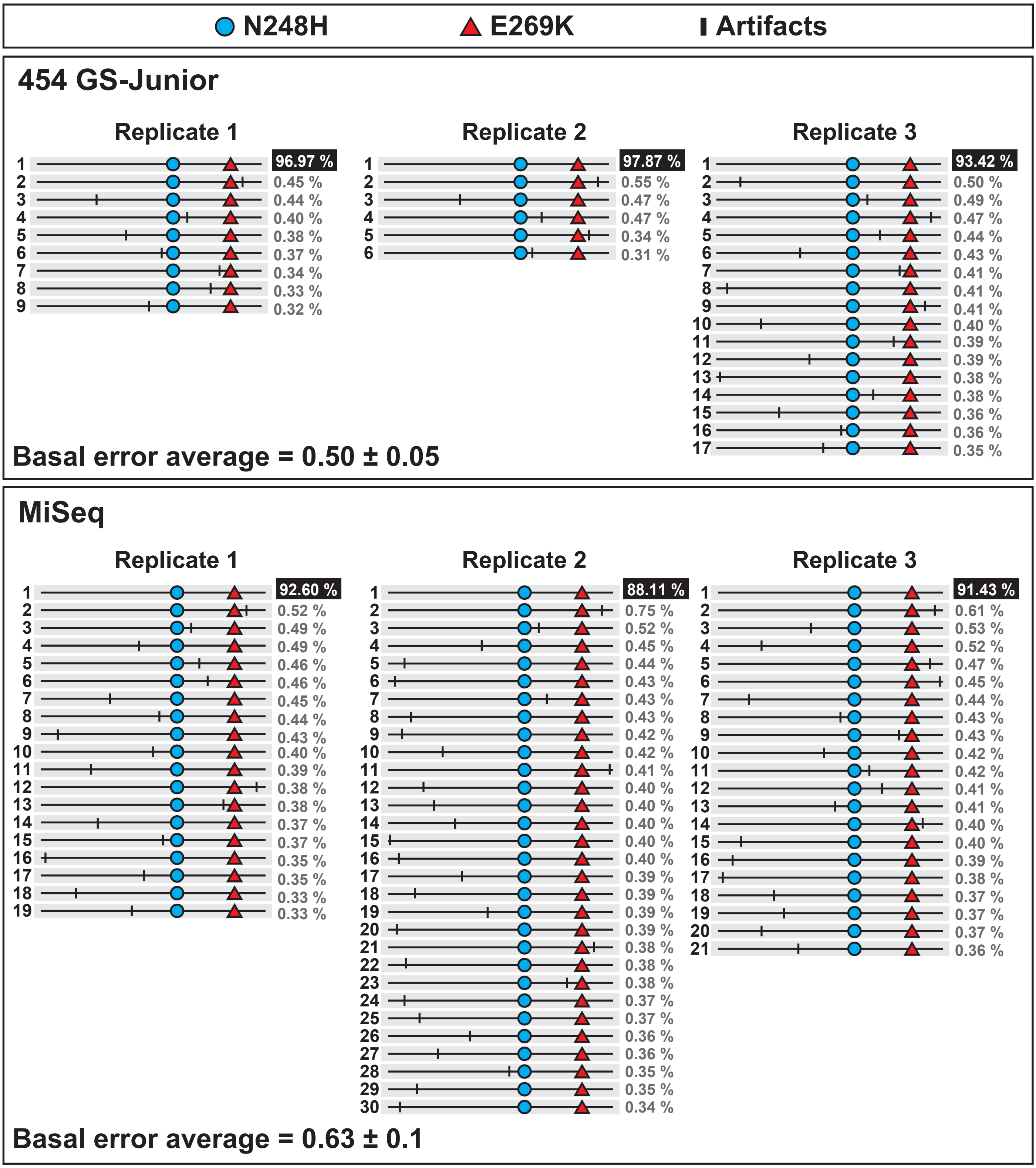

Figure S5. Control of basal error. A full-length HCV DNA encoding NS5A with amino acid substitutions N248K, E269K and A346V was used as a template to determine the basal error of the amplification and sequencing process using 454 GS-Junior and Illumina MiSeq platforms. Due to restriction size of amplicon length, only N248K and E269K were detected. Experiments were performed in triplicate. Haplotypes obtained after amplification and sequencing are numbered on the left of each replicate, and percentages of reads that include the indicated substitutions are shown on the right. Artifacts meansmutations other than those encoding N248K and E269K. Basal error average is the mean ± standard deviation of the haplotypes number 2, which are those including artifacts found at the highest frequency.
