# Supplementary material for: Pipeline for specific subtype amplification and drug resistance detection in hepatitis C virus
Source: BMC Infect Dis. 2018 Sep 3;18:446. doi: 10.1186/s12879-018-3356-6 (PMC6122477; doi:10.1186/s12879-018-3356-6)
Supplement: Supplementary file 12 — Figure S6. Theoretical study to define the reliable coverage needed to detect a mutant present at 1% in a viral population. (A) Confidence intervals (CI) of the observed proportions (given in the abscissa) of a variant amino acid present at 1% frequency in a viral population, with coverages varying from 500 to 10,000 reads (given in the ordinate) according to the binomial law. Left: 95% CI; Right: 99% CI. (B) Effect of the coverage at four different CIs (indicated in ordinate) considering that true variants (in blue) are present at 1%, and artifact variants (in pink) at 0.5%. The abscissa gives the percentage at which the two classes of variants are observed. Note that at high read coverages the overlap between true and artifact variants is minimal. (PDF 1158 kb) [file 12879_2018_3356_MOESM12_ESM.pdf]

Figure S6

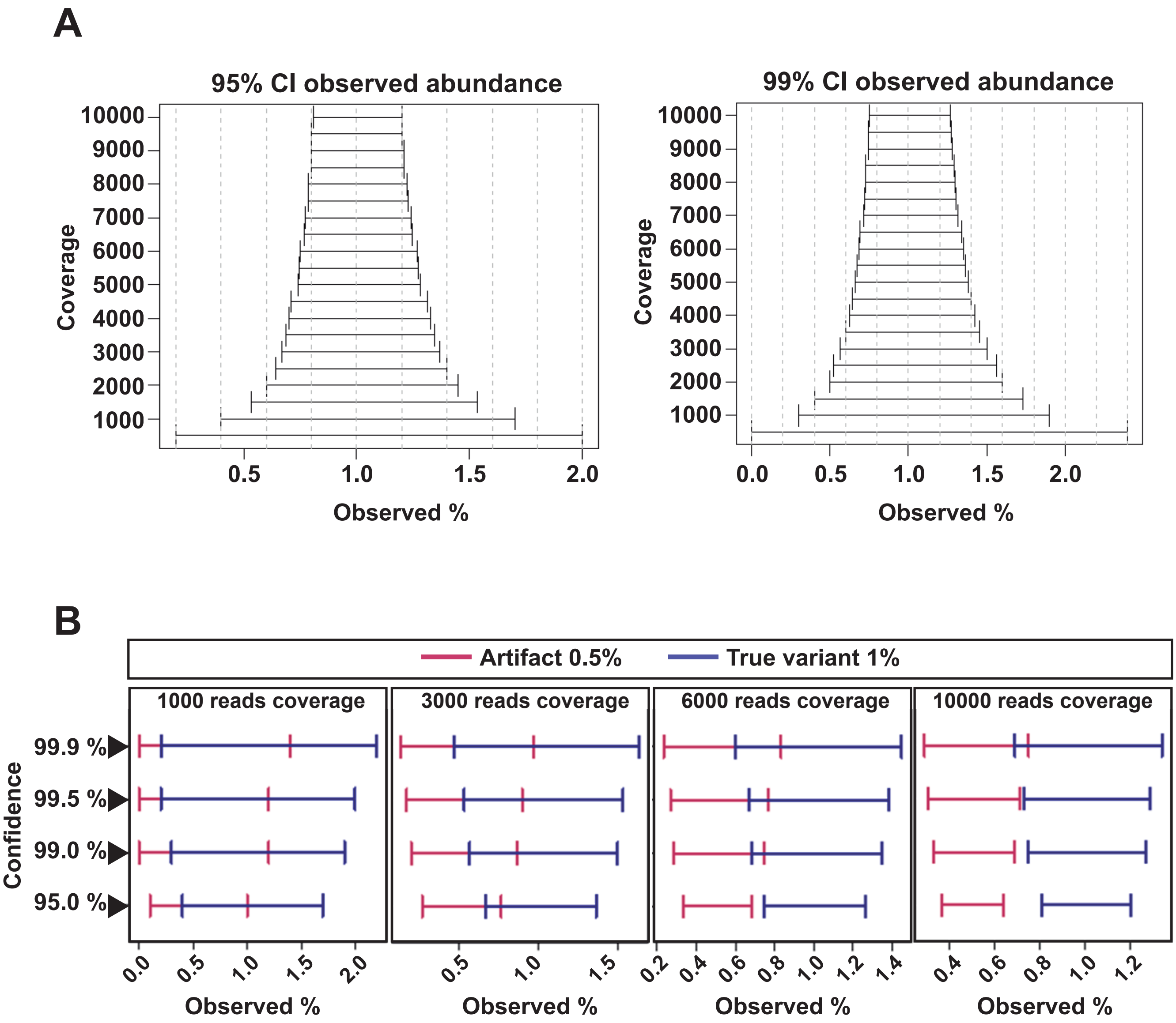

Figure S6. Theoretical study to define the reliable coverage needed to detect a mutant present at 1% in a viral population. (A) Confidence intervals (CI) of the observed proportions (given in the abscissa) of a variant amino acid present at 1% frequency in a viral population, with coverages varying from 500 to 10,000 reads (given in the ordinate) according to the binomial law. Left: 95% CI; Right: 99% CI. (B) Effect of the coverage at four different CIs (indicated in ordinate) considering that true variants (in blue) are present at 1%, and artifact variants (in pink) at 0.5%. The abscissa gives the percentage at which the two classes of variants are observed. Note that at high read coverages the overlap between true and artifact variants is minimal.
