# Supplementary material for: Pipeline for specific subtype amplification and drug resistance detection in hepatitis C virus
Source: BMC Infect Dis. 2018 Sep 3;18:446. doi: 10.1186/s12879-018-3356-6 (PMC6122477; doi:10.1186/s12879-018-3356-6)
Supplement: Supplementary file 5 — Figure S2. Subtype-specific oligonucleotides designed to sequence the NS5A-coding region. Residue numbering is according to the reference strain AF009606. Positions in red are conserved among the different subtypes, and positions with different colors are discriminatory of a specific subtype (color codes given in the left column at each panel). Discriminatory positions for genotype are highlighted in pink. (PDF 1014 kb) [file 12879_2018_3356_MOESM5_ESM.pdf]

Figure S2

RT-PCR NS5A (2)

(5'- 3') (Forward)

Subtypes	6227	6228	6229	6230	6231	6232	6233	6234	6235	6236	6237	6238	6239	6240	6241	6242	6243	6244	6245	6246	6247	6248	6249	6250	6251	6252	6253	6254	6255
1a								A	G	C	T	C	G	G	A	G	T	G	Y	A	C	C	A	C	T	C	C	R	T
1b								A	A	Y	G	A	G	G	A	C	T	G	C	T	C	C	A	C	G	C	C	A	T
2a								A	C	T	G	A	G	G	A	C	T	G	C	C	C	C	A	T	C	C	C	A	T
2b				G	A	T	C	A	C	T	G	A	A	G	A	T	T	G	C	C	C	A	G	T	C	C	C	A	T
2c											G	A	R	G	A	Y	T	G	C	C	C	Y	G	T	C	C	C	C	T
2j			G	G	A	T	C	A	C	T	G	A	G	G	A	C	T	G	Y	C	C	C	G	T					
3a				G	A	T	C	A	A	T	G	A	A	G	A	C	T	A	C	C	C	A	A	G	Y	C	C	T	T
4a				G	A	T	C	A	A	T	G	A	A	G	A	T	T	G	Y	T	C	C	A	C	Y	C	C	A	T
4d								A	A	C	G	A	G	G	A	C	T	G	C	T	C	T	A	C	T	C	C	T	T
4f	G	T	G	G	A	T	C	A	A	T	G	A	A	G	A	C	T	G	Y	C	C	Y	A	C	T				

(5'- 3') (Reverse)

Subtypes	6882	6881	6880	6879	6878	6877	6876	6875	6874	6873	6872	6871	6870	6869	6868	6867	6866	6865	6864	6863	6862	6861	6860	6859	6858	6857	6856	6855	6854	6853
1a				G	G	G	A	G	G	G	A	T	C	A	G	T	G	A	G	C	A	T	G	G	A					
1b				G	G	G	A	G	G	G	G	T	C	G	G	T	G	A	G	C	A	T								
2a			T	G	G	G	A	Y	G	G	A	T	C	T	G	T	T	A	G	C	A	T	G	G	A					
2b				G	G	G	A	C	G	G	G	T	C	T	G	T	C	A	A	C	A	T	G	G	A					
2c	T	G	T	G	G	G	A	Y	G	G	G	T	C	Y	G	T	T	A	G	C	A	T								
2j					G	G	A	Y	G	G	G	T	C	C	G	T	C	A	G	C	A	T	G	G	A					
3a									G	G	G	T	C	T	C	T	C	A	A	C	A	T	C	G	A	G	G	T	C	A
4a				G	T	G	A	T	G	G	G	T	C	T	G	T	C	A	R	C	A	T	G	G	A					
4d				G	A	G	A	T	G	G	A	T	C	T	G	T	C	A	G	C	A	T	A	G	A					
4f				G	G	G	A	T	G	G	G	T	C	T	G	T	A	A	G	C	A	T	G	G	A					

PCR NS5A (2.1)

(5'- 3') (Forward)

Subtypes	6299	6300	6301	6302	6303	6304	6305	6306	6307	6308	6309	6310	6311	6312	6313	6314	6315	6316	6317	6318	6319	6320	6321	6322	6323	6324	6325
1a	G	G	T	G	C	T	G	A	G	C	G	A	Y	T	T	T	A	A	G	A	C	C	T	G	G	C	T
1b	G	G	T	G	T	T	G	A	C	T	G	A	Y	T	T	C	A	A	G	A	C	C	T	G	G	C	T
2a	C	A	T	C	C	T	R	A	C	A	G	A	C	T	T	Y	A	A	A	A	A	Y	T	G	G	C	T
2b	C	A	T	Y	C	T	C	A	C	A	G	A	C	T	T	T	A	A	G	A	A	C	T	G	G	C	T
2c	R	A	T	C	C	T	G	A	C	A	G	A	C	T	T	Y	A	A	G	A	R	T	T	G	G	C	T
2j	C	A	T	C	T	T	G	A	C	T	G	A	C	T	T	Y	A	A	R	A	A	Y	T	G	G	C	T
3a	G	G	T	G	Y	T	G	T	C	Y	G	A	C	T	T	C	A	A	G	A	C	A	T	G	G	C	T
4a	C	G	T	G	C	T	G	A	G	T	G	A	C	T	T	C	A	A	G	A	C	G	T	G	G	C	T
4d	C	G	T	A	C	T	G	A	G	T	G	A	C	T	T	T	A	A	A	A	C	G	T	G	G	C	T
4f	C	G	T	A	Y	T	G	T	C	T	G	A	C	T	T	Y	A	A	G	A	C	Y	T	G	G	C	T

(5'- 3') (Reverse)

Subtypes	6735	6734	6733	6732	6731	6730	6729	6728	6727	6726	6725	6724	6723	6722	6721	6720	6719	6718	6717	6716	6715
1a	T	A	T	G	Y	A	G	G	C	G	C	A	C	C	C	C	G	T	C	C	A
1b	T	G	T	G	C	A	R	C	C	G	Y	A	C	C	C	C	R	T	C	C	A
2a	T	R	T	G	G	A	T	Y	T	G	C	A	C	T	C	C	G	T	C	C	A
2b	G	G	T	G	T	A	T	Y	T	G	C	A	C	C	C	C	G	T	C	C	A
2c	G	A	T	G	G	A	T	Y	T	G	R	A	C	Y	C	C	G	T	C	C	A
2j	G	R	T	G	G	A	T	C	T	G	Y	A	C	C	C	C	R	T	C	C	A
3a	G	G	T	G	G	A	G	T	C	T	C	A	C	C	C	C	R	T	C	C	A
4a	G	G	T	G	T	A	G	Y	C	T	G	A	Y	G	C	C	G	T	C	Y	A
4d	G	A	T	G	G	A	G	C	C	T	G	A	C	G	C	C	A	T	C	C	A
4f	G	G	T	G	A	A	K	T	C	T	R	A	C	R	C	C	A	T	C	C	A

PCR NS5A (2.2)

(5'- 3') (Forward)

Subtypes	6256	6257	6258	6259	6260	6261	6262	6263	6264	6265	6266	6267	6268	6269	6270	6271	6272	6273	6274	6275	6276	6277	6278	6279	6280	6281	6282
1a					C	G	G	T	T	C	C	T	G	G	C	T	A	A	G	G	G	A	C	A	T		
1b				C	C	G	G	C	T	C	G	T	G	G	C	T	A	A	G	G	G	A					
2a					C	G	G	M	T	C	V	T	G	G	C	T	C	C	G	C	G	A	Y	G	T		
2b					G	G	G	G	T	C	T	T	G	G	C	T	C	C	R	G	G	A					
2c	G	C	T	C	Y	R	G	C	T	C	A	T	G	G	C	T	Y	C	G	A	G	A					
2j						G	G	C	T	C	T	T	G	G	C	T	T	C	G	C	G	A	C	A	T	A	T
3a					C	G	G	Y	G	A	T	T	G	G	C	T	G	C	G	T	A	C	C	A	T		
4a					C	G	A	A	T	C	T	T	G	G	C	T	R	T	G	G	G	A	G	G	T		
4d	G	T	G	A	T	C	G	C	T	C	T	T	G	G	T	T	A	T	G	G	G	A	G	A	T		
4f	G	T	G	A	C	T	C	A	T	C	Y	T	G	G	C	T	R	T	G	G	G	A					

(5'- 3') (Reverse)

Subtypes	6799	6798	6797	6796	6795	6794	6793	6792	6791	6790	6789	6788	6787	6786	6785	6784	6783	6782	6781	6780	6779	6778	6777	6776	6775	6774	6773	6772	6771
1a	T	C	G	T	G	K	A	G	T	C	C	Y	A	C	T	C	T	G	A	A	Y	G	A						
1b	T	G	G	T	T	G	A	G	C	C	C	G	A	C	C	T	G	G	A	A	T	G	T						
2a							A	G	C	C	C	A	A	C	G	C	W	R	A	A	C	G	A	G	A	C	C	T	C
2b	G	A	A	T	T	G	A	G	G	C	C	Y	A	C	R	G	T	G	A	A	C	G	T						
2c	G	A	G	T	T	G	A	G	M	C	C	C	A	C	G	C	W	G	A	A	C	G	A						
2j	G	A	G	T	T	G	A	G	G	C	C	C	A	C	G	C	T	A	A	A	C	G	A						
3a	G	A	A	T	T	C	A	W	C	C	C	T	A	C	C	R	T	G	A	A	A	G	T	G	A	T			
4a	G	A	A	T	T	G	A	G	T	C	C	T	A	C	Y	G	A	G	A	A	C	G	A						
4d	G	T	G	T	T	G	A	G	G	C	C	C	A	C	T	G	T	A	A	A	G	G	A						
4f		A	G	T	T	R	A	G	G	C	C	C	A	C	G	G	A	G	A	A	C	G	T						

Figure S2. Subtype-specific oligonucleotides designed to sequence the NS5A-coding region. Residue numbering is according to the reference strain AF009606. Positions in red are conserved among the different subtypes, and positions with different colors are discriminatory of a specific subtype (color codes given in the left column at each panel). Discriminatory positions for genotype are highlighted in pink.
