# Supplementary material for: Pipeline for specific subtype amplification and drug resistance detection in hepatitis C virus
Source: BMC Infect Dis. 2018 Sep 3;18:446. doi: 10.1186/s12879-018-3356-6 (PMC6122477; doi:10.1186/s12879-018-3356-6)
Supplement: Supplementary file 14 — Figure S8. Definition of the reference sequence for the ten subtypes under study. Nucleotide numbering at the beginning and at the end of NS3, NS5A and NS5B indicates the length of each coding region. Amino acid number of each coding region is indicated between the arrows. Positions at which resistance-associated substitutions (RAS) have been described are shown above the coding regions, and are included in our analyses. Alignments include a reference sequence for each subtype (1a, 1b, 2a, 2b, 2c, 2j, 3a, 4a, 4d, 4f); amino acid numbering above the boxes is indicated. Each reference sequence is defined as the most frequent amino acid at each position of the sequence ensemble for each subtype described in Table S1. Nucleotide and amino acid numbering are based on the HCV strain H77 (GenBank accession AF009606). (PDF 849 kb) [file 12879_2018_3356_MOESM14_ESM.pdf]

Figure S8

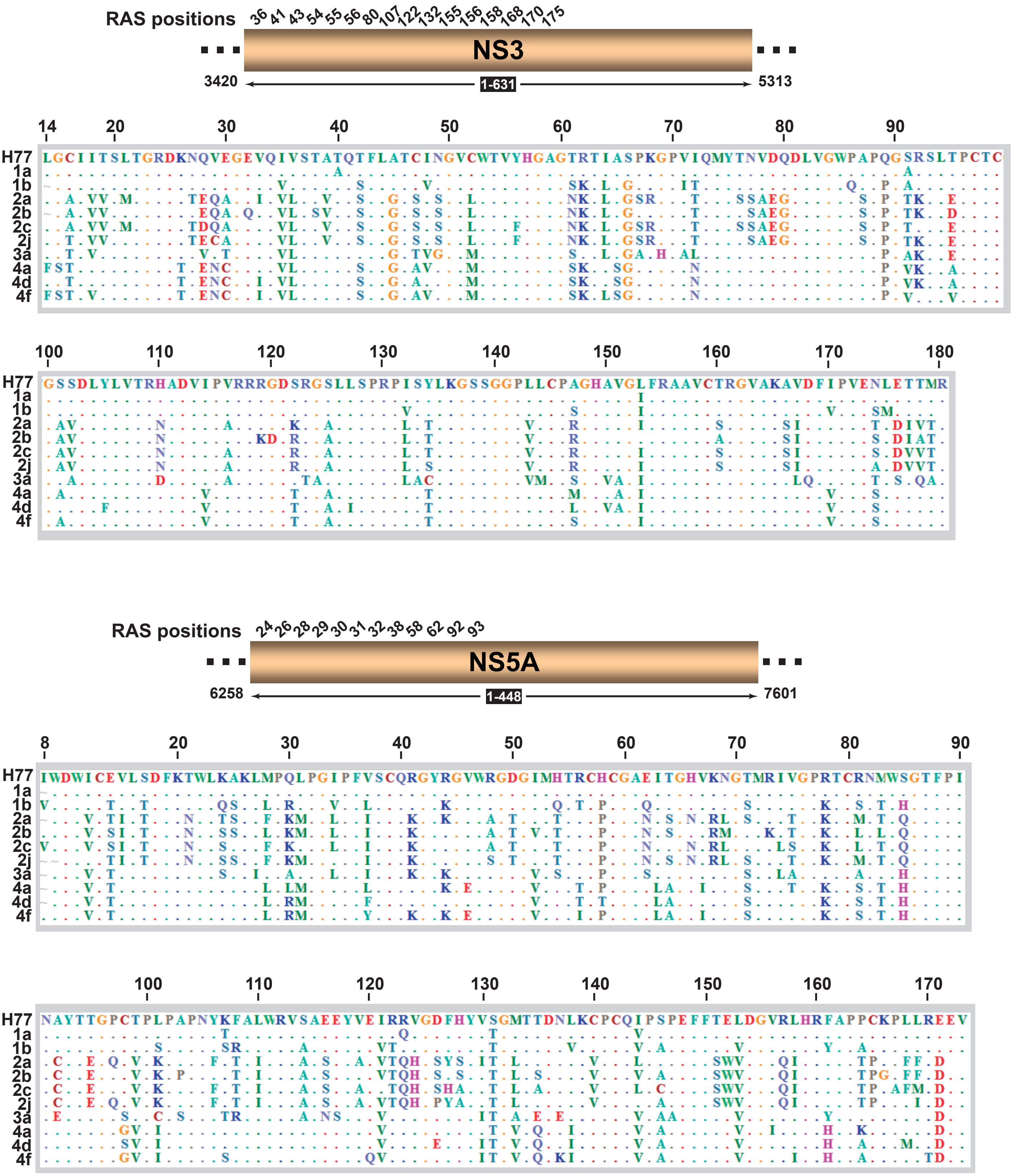
RAS positions
36 41 43 54 55 56 80 107 122 132 155 156 158 168 170 175
NS3
3420
1-631
5313
RAS positions
24 26 28 29 30 31 32 38 58 62 92 93
NS5A
6258
1-448
7601
H77
1a
1b
2a
2b
2c
2j
3a
4a
4d
4f

Figure S8 *(continued)*

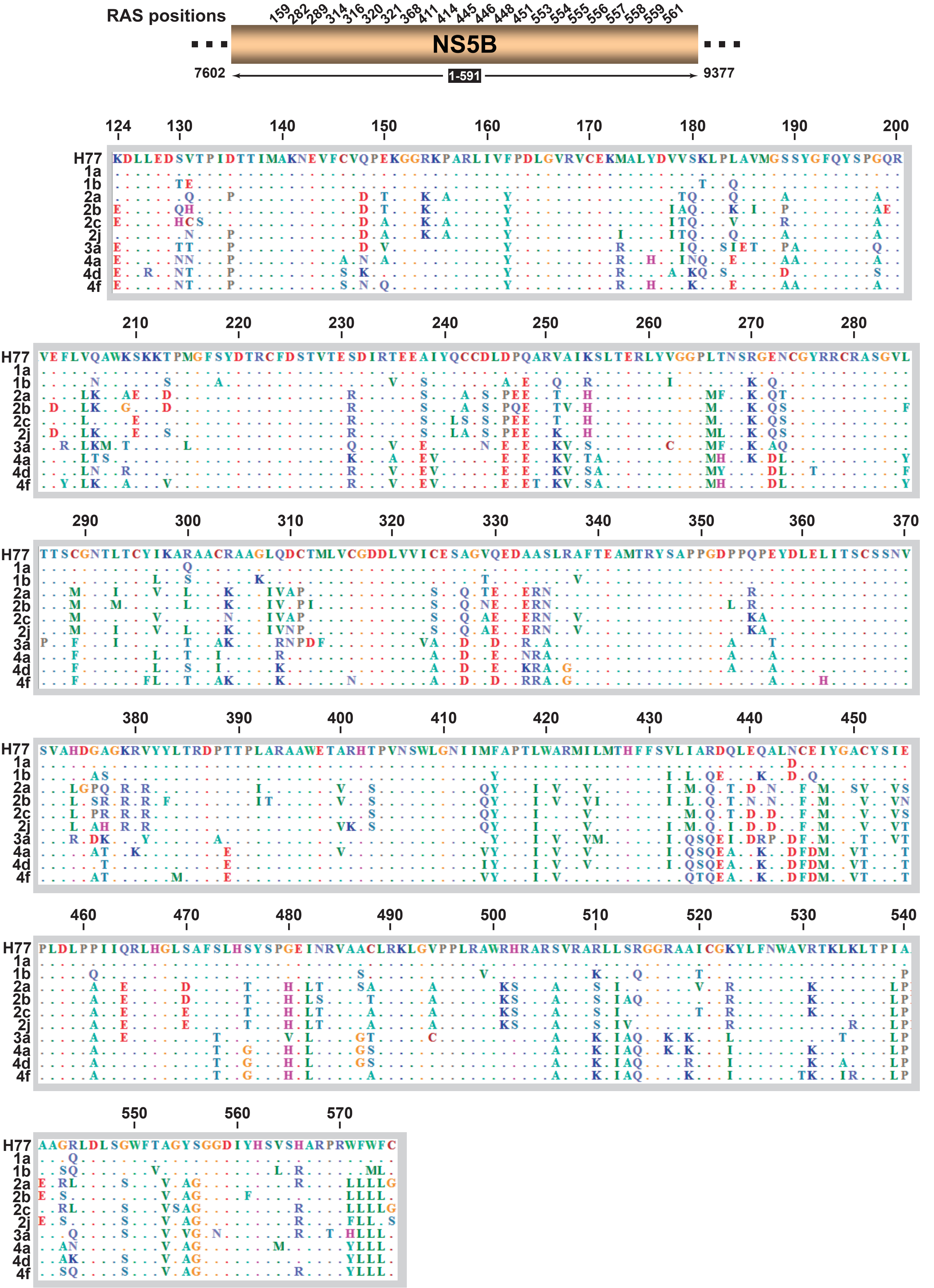

Figure S8. Definition of the reference sequence for the ten subtypes under study. Nucleotide numbering at the beginning and at the end of NS3, NS5A and NS5B indicates the length of each coding region. Amino acid number of each coding region is indicated between the arrows. Positions at which resistance-associated substitutions (RAS) have been described are shown above the coding regions, and are included in our analyses. Alignments include a reference sequence for each subtype (1a, 1b, 2a, 2b, 2c, 2j, 3a, 4a, 4d, 4f); amino acid numbering above the boxes is indicated. Each reference sequence is defined as the most frequent amino acid at each position of the sequence ensemble for each subtype described in Table S1. Nucleotide and amino acid numbering are based on the HCV strain H77 (GenBank accession AF009606).
