# Supplementary material for: Pipeline for specific subtype amplification and drug resistance detection in hepatitis C virus
Source: BMC Infect Dis. 2018 Sep 3;18:446. doi: 10.1186/s12879-018-3356-6 (PMC6122477; doi:10.1186/s12879-018-3356-6)
Supplement: Supplementary file 15 — Figure S9. Ultra-deep sequencing of viral samples from an G1a HCV-infected patient subjected to sequential DAA treatments. First panel: Representation of the viral load [international units (IU)/ml] as a function of time (in weeks). A yellow background indicates period in which antiviral treatment was applied (LDV: ledipasvir; SOF: sofosbuvir; RBV: ribavirin; DCV: daclatasvir; SMV: simeprevir). Viral relapse ocurred after the first two treatments, and SVR (sustained virological response) was achieved after the third treatment. Viral RNAs from four sequential samples (termed 1 to 4, indicated with arrows) were used to amplify NS3- (PCR 1.1 in Fig. 2), NS5A- (PCR 2.1 in Fig. 2), and NS5B-coding regions (PCRs 3.1 and 3.2 in Fig. 2) using MiSeq platforms. Subsequent panels: For each coding region (NS3, NS5A, NS5B) variations in mutation frequencies (as percentage of individual mutations) are represented as mutational waves (mutations that increase or decrease in frequency, relative to the previous or subsequent sample analyzed), and in a heat map of frequencies (color boxed below the graphics). Substitutions written in red confer resistance to the DAAs included in the treatment combinations, or are described in the literature (European Association for the Study of the Liver. Electronic address, 2017; Sarrazin, [9]; Lontok et al., [14]). Asteriscs in the heat map of NS5A-coding region in samples at viral relapse 2 and 4 highlight RAS that confer resistance to ledipasvir and daclatasvir, respectively. Haplotypes and their percentages (proportion of reads that include the indicated substitutions) are depicted on the right of each panel. (PDF 1030 kb) [file 12879_2018_3356_MOESM15_ESM.pdf]

Figure S9

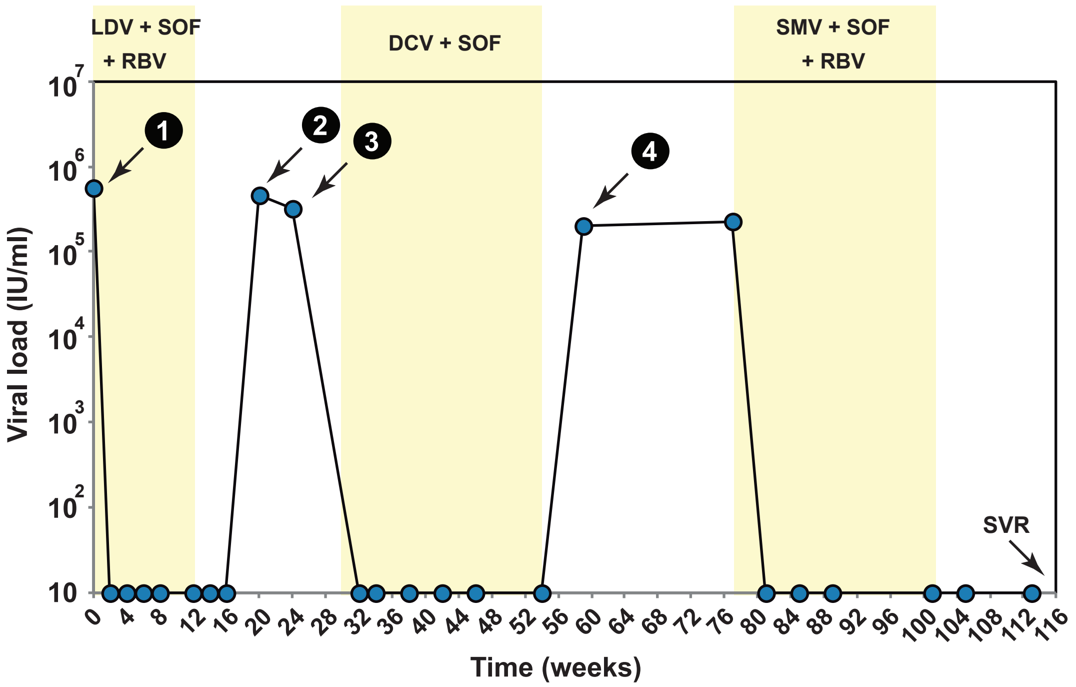
LDV + SOF + RBV
DCV + SOF
SMV + SOF + RBV
Viral load (IU/ml)
Time (weeks)
SVR

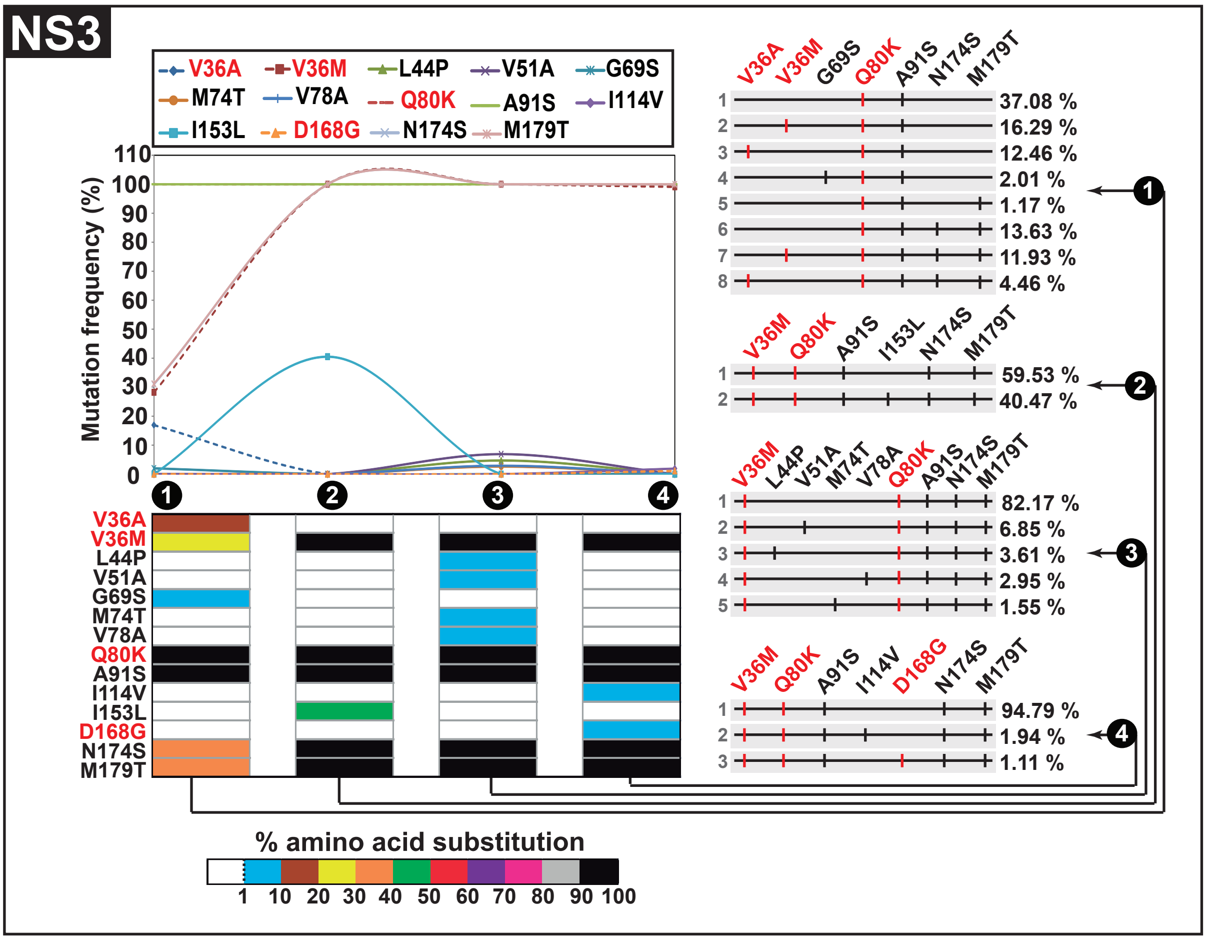
NS3
V36A
V36M
L44P
V51A
G69S
M74T
V78A
Q80K
A91S
I114V
I153L
D168G
N174S
M179T
Mutation frequency (%)
37.08 %
16.29 %
12.46 %
2.01 %
1.17 %
13.63 %
11.93 %
4.46 %
59.53 %
40.47 %
82.17 %
6.85 %
3.61 %
2.95 %
1.55 %
94.79 %
1.94 %
1.11 %
% amino acid substitution

Figure S9 *(continued)*

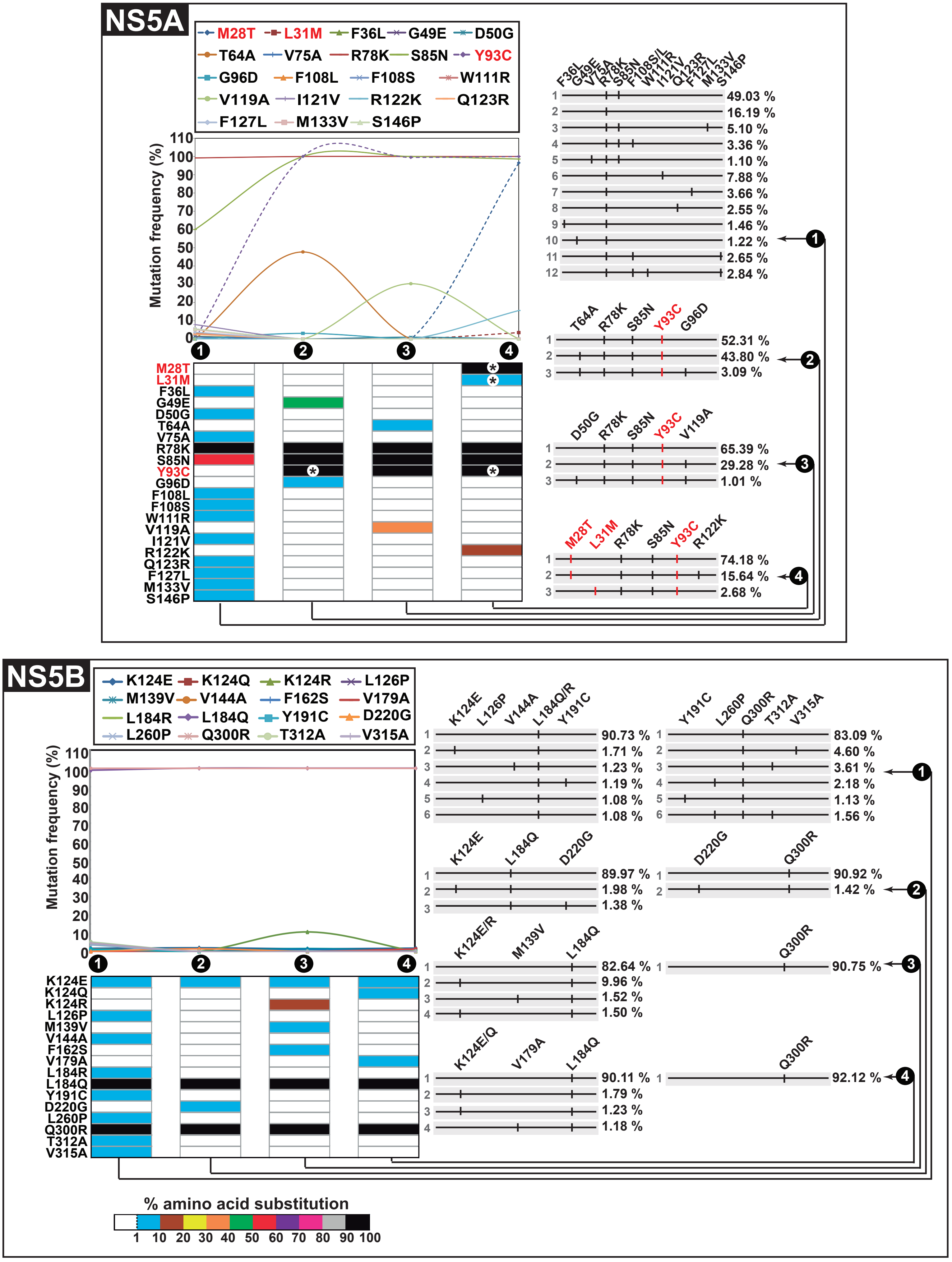

Figure S9. Ultra-deep sequencing of viral samples from an G1a HCV-infected patient subjected to sequential DAA treatments. First panel: Representation of the viral load [international units (IU)/ml] as a function of time (in weeks). A yellow background indicates period in which antiviral treatment was applied (LDV: ledipasvir; SOF: sofosbuvir; RBV: ribavirin; DCV: daclatasvir; SMV: simeprevir). Viral relapse ocurred after the first two treatments, and SVR (sustained virological response) was achieved after the third treatment. Viral RNAs from four sequential samples (termed 1 to 4, indicated with arrows) were used to amplify NS3- (PCR 1.1 in Figure 2), NS5A- (PCR 2.1 in Figure 2), and NS5B-coding regions (PCRs 3.1 and 3.2 in Figure 2) using MiSeq platforms. Subsequent panels: For each coding region (NS3, NS5A, NS5B) variations in mutation frequencies (as percentage of individual mutations) are represented as mutational waves (mutations that increase or decrease in frequency, relative to the previous or subsequent sample analyzed), and in a heat map of frequencies (color boxed below the graphics). Substitutions written in red confer resistance to the DAAs included in the treatment combinations, or are described in the literature (European Association for the Study of the Liver. Electronic address, 2017; Lontok et al., 2015; Sarrazin, 2016). Asteriscs in the heat map of NS5A-coding region in samples at viral relapse 2 and 4 highlight RAS that confer resistance to ledipasvir and daclatasvir, respectively. Haplotypes and their percentages (proportion of reads that include the indicated substitutions) are depicted on the right of each panel.
