# Supplementary material for: Pipeline for specific subtype amplification and drug resistance detection in hepatitis C virus
Source: BMC Infect Dis. 2018 Sep 3;18:446. doi: 10.1186/s12879-018-3356-6 (PMC6122477; doi:10.1186/s12879-018-3356-6)
Supplement: Supplementary file 13 — Figure S7. Control of PCR-based recombination. A full-length reference (wt) viral HCV DNA was mixed with HCV DNA with mutations A7010C and G7073A (corresponding to amino acid substitutions N248 K and E269K in NS5A) at a 90:10 ratio (depicted on the left of the top box). The total number of DNA molecules was 100,000. The mixture was used as a template to determine the degree of recombination after the amplification and sequencing process, following four different protocols (termed 1 to 4), and two next-generation sequencing platforms (454 GS-Junior and Illumina MiSeq). Protocol conditions are described in Materials and Methods. Top box depicts the four types of expected molecules; wt clone, mutant N248 K/E269K, and recombinant molecules N248 K/wt and wt/E269K. Each experiment was performed in duplicate (replicates 1 and 2), and the average frequency (%) of both replicates is shown on the right of each molecule; n.d. means not detected. The percentage of reads that include the indicated substitutions is represented for each haplotype and protocol in the three panels at the bottom. (PDF 399 kb) [file 12879_2018_3356_MOESM13_ESM.pdf]

Figure S7

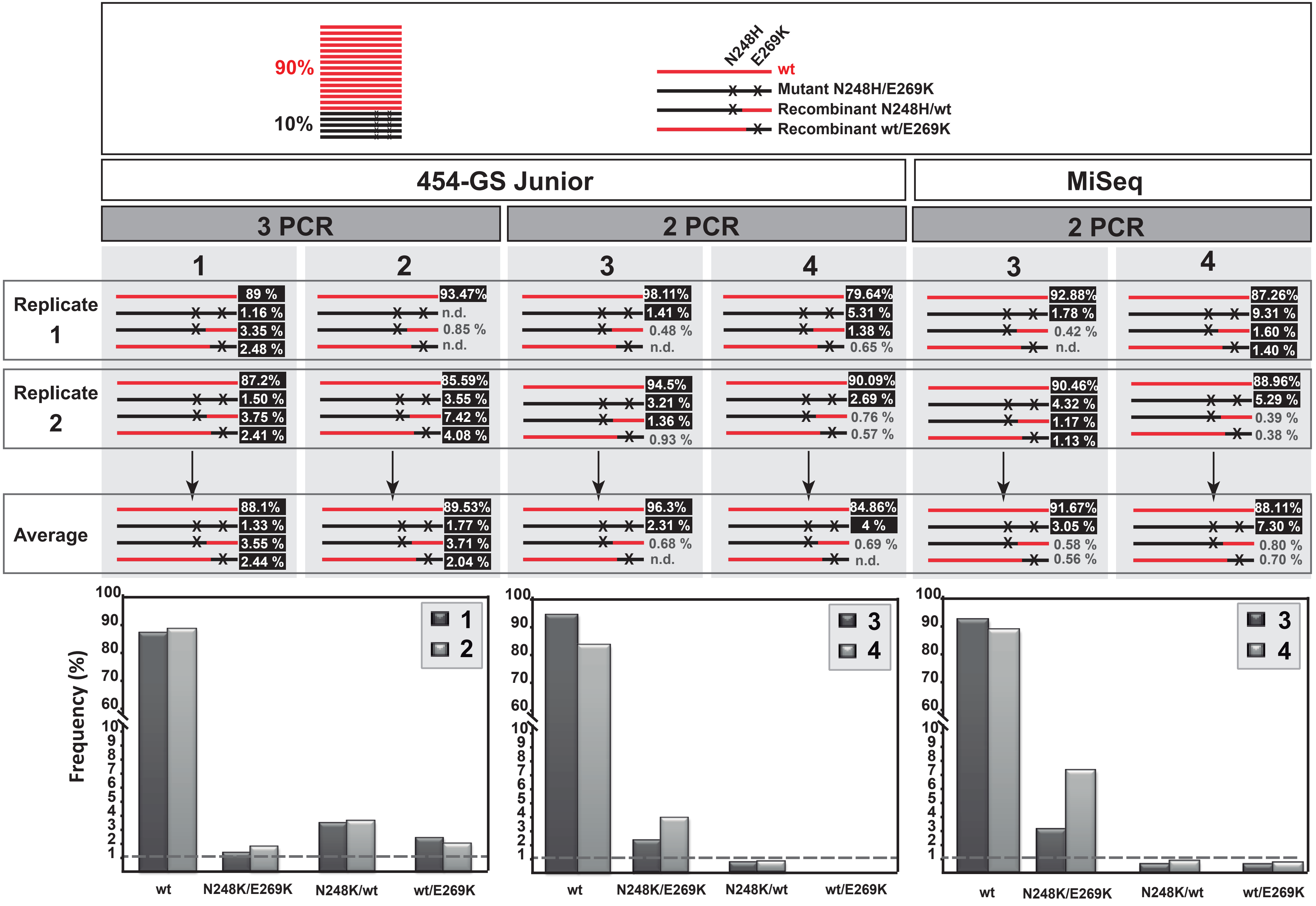

90%
10%
N248H
E269K
wt
Mutant N248H/E269K
Recombinant N248H/wt
Recombinant wt/E269K
454-GS Junior
MiSeq
3 PCR
2 PCR
2 PCR
1
2
3
4
3
4
Replicate 1
89 %
1.16 %
3.35 %
2.48 %
93.47%
n.d.
0.85 %
n.d.
98.11%
1.41 %
0.48 %
n.d.
79.64%
5.31 %
1.38 %
0.65 %
92.88%
1.78 %
0.42 %
n.d.
87.26%
9.31 %
1.60 %
1.40 %
Replicate 2
87.2%
1.50 %
3.75 %
2.41 %
85.59%
3.55 %
7.42 %
4.08 %
94.5%
3.21 %
1.36 %
0.93 %
90.09%
2.69 %
0.76 %
0.57 %
90.46%
4.32 %
1.17 %
1.13 %
88.96%
5.29 %
0.39 %
0.38 %
Average
88.1%
1.33 %
3.55 %
2.44 %
89.53%
1.77 %
3.71 %
2.04 %
96.3%
2.31 %
0.68 %
n.d.
84.86%
4 %
0.69 %
n.d.
91.67%
3.05 %
0.58 %
0.56 %
88.11%
7.30 %
0.80 %
0.70 %
Frequency (%)
wt
N248K/E269K
N248K/wt
wt/E269K

Figure S7. Control of PCR-based recombination. A full-length reference (wt) viral HCV DNA was mixed with HCV DNA with mutations A7010C and G7073A (corresponding to amino acid substitutions N248K and E269K in NS5A) at a 90:10 ratio (depicted on the left of the top box). The total number of DNA molecules was 100,000. The mixture was used as a template to determine the degree of recombination after the amplification and sequencing process, following four different protocols (termed 1 to 4), and two next-generation sequencing platforms (454 GS-Junior and Illumina MiSeq). Protocol conditions are described in Materials and Methods. Top box depicts the four types of expected molecules; wt clone, mutant N248K/E269K, and recombinant molecules N248K/wt and wt/E269K. Each experiment was performed in duplicate (replicates 1 and 2), and the average frequency (%) of both replicates is shown on the right of each molecule; n.d. means not detected. The percentage of reads that include the indicated substitutions is represented for each haplotype and protocol in the three panels at the bottom.
